# Considerations for Patient Privacy of Large Language Models in Health Care: Scoping Review

**DOI:** 10.2196/76571

**Published:** 2025-11-21

**Authors:** Xiaoying Zhong, Siyi Li, Zhao Chen, Long Ge, Dongdong Yu, Shijia Wang, Liangzhen You, Hongcai Shang

**Affiliations:** 1 Dongzhimen Hospital Beijing University of Chinese Medicine Beijing, null China; 2 Key Laboratory of Chinese Internal Medicine of the Ministry of Education Beijing University of Chinese Medicine Beijing, null China; 3 Institute of Basic Research in Clinical Medicine China Academy of Chinese Medical Sciences Beijing China; 4 Department of Health Policy and Health Management School of Public Health Lanzhou University Lanzhou China; 5 Evidence-Based Social Science Research Center School of Public Health Lanzhou University Lanzhou China; 6 WHO Collaborating Center for Guideline Implementation and Knowledge Translation Lanzhou China; 7 Second Clinical College of Guangzhou University of Chinese Medicine Guangzhou China; 8 Dongfang Hospital Beijing University of Chinese Medicine Beijing China

**Keywords:** patient privacy, patient health information, large language models, health care, scoping review

## Abstract

**Background:**

The application of large language models (LLMs) in health care holds significant potential for enhancing patient care and advancing medical research. However, the protection of patient privacy remains a critical issue, especially when handling patient health information (PHI).

**Objective:**

This scoping review aims to evaluate the adequacy of current approaches and identify areas in need of improvement to ensure robust patient privacy protection in the existing studies about PHI-LLMs within the health care domain.

**Methods:**

A search of the literature published from January 1, 2022, to July 20, 2025, was performed on July 20, 2025, using 2 databases (PubMed and Embase). This scoping review focused on the following three research questions: (1) What studies on the development and application of LLMs using PHI currently exist within the health care domain? (2) What patient privacy considerations are addressed in existing PHI-LLMs research, and are these measures sufficient? (3) How can future research on the development and application of LLMs using PHI better protect patient privacy? Studies were included if they focused on the development and application of LLMs within health care using PHI, encompassing activities such as model construction, fine-tuning, optimization, testing, and performance comparison. Eligible literature comprised original research articles written in English. Conversely, studies were excluded if they used publicly available datasets, under the assumption that such data have been adequately deidentified. Additionally, non-English publications, reviews, abstracts, incomplete reports, and preprints were excluded from the review due to the lack of rigorous peer review.

**Results:**

This study systematically identified 9823 studies on PHI-LLM and included 464 studies published between 2022 and 2025. Among the 464 studies, (1) a small number of studies neglected ethical review (n=45, 9.7%) and patient informed consent (n=148, 31.9%) during the research process, (2) more than a third of the studies (n=178, 38.4%) failed to report whether to implement effective measures to protect PHI, and (3) there was a significant lack of transparency and comprehensive detail in anonymization and deidentification methods.

**Conclusions:**

We propose comprehensive recommendations across 3 phases—study design, implementation, and reporting—to strengthen patient privacy protection and transparency in PHI-LLM. This study emphasizes the urgent need for the development of stricter regulatory frameworks and the adoption of advanced privacy protection technologies to effectively safeguard PHI. It is anticipated that future applications of LLMs in the health care field will achieve a balance between innovation and robust patient privacy protection, thereby enhancing ethical standards and scientific credibility.

## Introduction

The application of large language models (LLMs) within the medical domain is undergoing rapid growth [1,2]. Key areas of investigation include assisted diagnosis [3] and the structured representation of electronic health records [4]. These models exhibit considerable potential, with preliminary findings from research and practical implementations showing promising results. LLMs through pretraining and fine-tuning on extensive datasets containing medical literature, clinical records, and biomedical knowledge bases, leverage deep learning methodologies to develop rich linguistic representations and demonstrate robust contextual understanding and knowledge integration [5]. This results in significantly enhanced performance compared with traditional language processing tools across natural language understanding, pattern recognition, and correlation analysis, with notable advantages in processing intricate medical data and facilitating cross-domain knowledge transfer [2]. While the pursuit of enhanced model performance remains crucial, ensuring robust protection of patient privacy and data security remains a paramount concern and a fundamental requirement for the responsible and sustainable advancement of LLM applications in health care. This presents a complex challenge that necessitates both technical and regulatory solutions [6-8].

A substantial portion of current research on LLMs for health care applications uses publicly available resources [9], such as the Medical Information Mart for Intensive Care-IV [10]. However, some studies are conducted using internal patient health information (PHI) repositories, which often contain highly sensitive personally identifiable information (PII) [11], including patient names, medical record numbers, age, zip code, admission date, and so on. This practice necessitates robust data governance frameworks to ensure patient privacy and data security. In conventional approaches to developing computer models using patient data, researchers typically processed and trained data within local or strictly regulated environments. This practice inherently reduced the risk of sensitive data compromise during transmission and storage [12,13]. However, deploying LLMs on the cloud often requires uploading vast amounts of raw medical data directly to remote servers. These servers may be distributed across different regions and are frequently not entirely under the control of health care institutions or researchers [8,14]. Beyond these conventional risks, LLMs introduce additional privacy concerns [15] due to their generative nature; they may inadvertently reproduce sensitive information learned during training, and their large scale and complexity expose them to attacks such as model inversion or prompt injection. Together, these factors make the protection of medical data in LLM deployment especially challenging. Recent studies have highlighted concrete privacy threats associated with LLM use: fine-tuning can significantly increase PII memorization and vulnerability to leakage attacks, especially when tuning specific layers of the model [16]; novel mitigation strategies like “Whispered Tuning,” which combine PII redaction, differential privacy, and output filtering, can markedly reduce leakage while preserving performance [17]; and beyond memorization, pretrained LLMs can infer personal attributes (eg, location, income, and gender) from seemingly innocuous text with high accuracy [18]. Consequently, data transmission and storage processes may encounter heightened risks of security vulnerabilities and unauthorized access. Whether through misuse by internal personnel or external cyberattacks, PHI is at risk of improper use or malicious disclosure [15].

Therefore, reconciling the potential of LLMs to enhance health care quality and efficiency with the imperative to protect patient privacy represents a significant challenge requiring careful consideration. The regulatory oversight of LLMs processing PHI is governed by a complex patchwork of national and international privacy laws, including the General Data Protection Regulation in the European Union and the Health Insurance Portability and Accountability Act (HIPAA) in the United States [6]. Besides, Transparent Reporting of a Multivariable Prediction Model for Individual Prognosis or Diagnosis (TRIPOD)-LLM [19] is an extension of the TRIPOD+AI statement, addressing the unique challenges of LLMs in biomedical and health care applications. There is a growing consensus within the academic community regarding the paramount importance of patient privacy protection in LLM research, with numerous concerns and queries being raised concerning the potential risks to sensitive data [20,21]. A range of privacy-preserving techniques is being widely considered and adopted within the health care domain to ensure data security and regulatory compliance. These include established methods such as deidentification [22,23], differential privacy [24], federated learning [25], and homomorphic encryption [26,27].

This leads us to our core research question: What measures are being used to protect patient privacy in the PHI-LLMs in the health care field, and are these measures sufficient? Although there are some systematic reviews or scope reviews of LLM research in health care, no scoping review has been published on this critical issue. The primary objective of this study is to conduct a scoping review of the existing literature on PHI-LLMs in health care, evaluate the adequacy of current approaches, and identify areas in need of improvement to ensure robust patient privacy protection.

## Methods

### Study Design

This scoping review was guided by the framework for scoping studies by Arksey and O’Malley [28]. Besides, the study reporting followed the Preferred Reporting Items for Systematic Reviews and Meta-Analyses extension for Scoping Reviews (PRISMA-ScR; checklist provided in Multimedia Appendix 1) [29]. We focused on the following three research questions: (1) What studies on the development and application of LLMs using PHI currently exist within the health care domain? (2) What patient privacy considerations are addressed in existing PHI-LLMs research, and are these measures sufficient? (3) How can future research on the development and application of LLMs using PHI better protect patient privacy?

### Eligibility Criteria

The inclusion and exclusion criteria for studies are shown in Textbox 1.

**Inclusion and exclusion criteria.**

**Inclusion criteria**

- Studies were included if they focused on the development and application of large language models within health care using personal health information, encompassing activities such as model construction, fine-tuning, optimization, testing, and performance comparison.
- Eligible literature comprised original research articles written in English.

**Exclusion criteria**

- Studies used publicly available datasets, under the assumption that such data have been adequately deidentified.
- Studies that were not reviews, abstracts, incomplete reports, or preprints were excluded from the review due to the lack of rigorous peer review.

### Data Sources, Search Strategy, and Study Selection

We searched PubMed and Embase for studies published between January 1, 2022, and July 20, 2025. This timeframe was chosen to coincide with the release and rapid adoption of advanced LLMs (eg, GPT-3.5 and ChatGPT) and the subsequent surge of their applications in health care. Earlier studies (published before 2022), which primarily investigated transformer-based or nongenerative models, were excluded as they fell outside the scope of this review. The search strategies were drafted by ZXY and further refined through team discussion. The final search strategy can be found in Multimedia Appendix 2. The final search results were exported into EndNote X9, and duplicates were removed by a library technician.

First, the titles and abstracts of identified studies were independently screened by 2 researchers (ZXY and LSY) based on the inclusion and exclusion criteria. Any disagreements between the reviewers were resolved through group discussions involving at least 2 researchers to ensure consensus and maintain the integrity of the selection process. The full-text review was also conducted by 2 researchers (ZXY and LSY) independently, with conflicts resolved through whole-group discussion.

### Data Extraction

The data extraction form was initially drafted by ZXY based on the study objectives and research questions. Following the draft, the form was refined through group discussions to develop a preliminary extraction template. To ensure consistency in the definitions and extraction criteria, 10 articles were randomly selected for a pilot extraction. Feedback from this pilot phase was used to finalize the extraction form. Subsequently, ZXY and LSY independently extracted data from the included studies. Any conflicts or discrepancies encountered during the extraction process were resolved through comprehensive group discussions involving all researchers to maintain the integrity and consistency of the data extraction.

The extracted data encompassed three main categories:

1. General characteristics of included studies: this included the first author’s name, publication year, country, the name and type of the LLMs used or developed, the disease domain as classified by the World Health Organization, and the type of tasks based on the classification outlined in the previous article [9].
2. General characteristics of clinical design: this section captured the sample size, the number of centers, the type of data used, the data collection method, the reporting statement they followed, and whether the study protocol was registered or not. The name of the LLM used or developed, the research objective, and the deployment type.
3. Patient privacy protection considerations: this part focused on the data source, the purpose of using PHI, whether the study underwent ethical review or approval, the declaration of data availability, whether patient consent was obtained, and the PHI protection techniques used.

All extracts are based on reports from the included study itself.

### Data Analysis

Descriptive analyses were performed to summarize the characteristics of the included studies. Categorical variables were summarized as frequencies and percentages. Sample size was further categorized into predefined ranges and presented as frequencies and percentages, while other continuous or count-based variables were directly summarized as reported. All results were presented in tables or figures according to the main domains of study characteristics, data characteristics, and privacy protection measures.

### Patient and Public Involvement

As this was a scoping review of previously published research, no patients or the public were involved in the design of this study.

## Results

### Selection of Sources of Evidence

**Figure 1:**
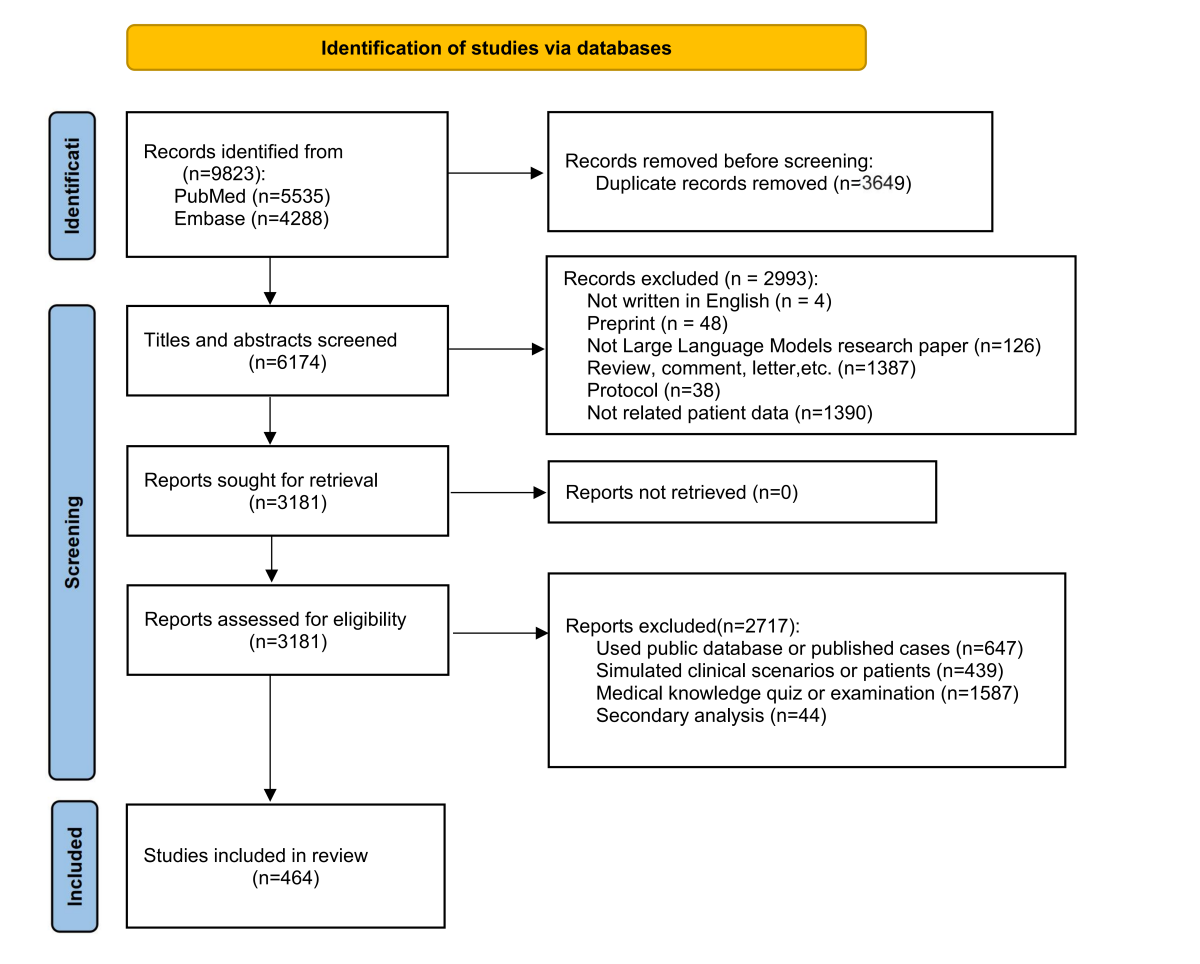
PRISMA (Preferred Reporting Items for Systematic reviews and Meta-Analyses) study selection diagram.

After removing duplicates, a total of 6174 citations were identified through searches of electronic databases and references in review articles. Based on their titles and abstracts, 3181 full-text articles were retrieved and assessed for eligibility. Of these, 2993 were excluded for the following reasons: 4 were not written in English, 48 were preprint papers, 126 were unrelated to LLM research, 1387 were reviews, comments, or letters, 38 were protocols, and 1390 did not involve relevant patient data. Following the eligibility assessment, a total of 2717 records were excluded for the following reasons: 647 used only public databases or previously published cases, 439 simulated clinical scenarios or patients rather than using real-world data, 1587 focused on medical knowledge quizzes or examinations, and 44 represented secondary analyses. Ultimately, 464 studies were deemed eligible for this review (Figure 1) [30]. The specific references included in the analysis are listed in the Multimedia Appendix 3.

### General Characteristics of the Included Studies

To provide an overview of the studies included in this review, we summarize their general characteristics in Table 1. This review encompasses studies published between January 1, 2022, and July 20, 2025. Of the 464 studies included in this review, the majority were published in 2025 (n=256, 55.2%), with a substantial proportion appearing in 2024 (n=188, 40.5%). In contrast, only a small number of studies were published in 2023 (19/464, 4.1%) and 2022 (1/464, 0.2%). This temporal distribution underscores the rapid and recent surge of research on LLM applications in health care, with most evidence emerging within the past 2 years. Based on the institutions of the first authors, the largest proportion of studies originated from the United States (153/464, 33.0%), followed by China (94/464, 20.3%) and Germany (37/464, 8.0%). Moderate contributions were observed from Turkey (31/464, 6.7%), Italy (17/464, 3.7%), Israel (13/464, 2.8%), South Korea (12/464, 2.6%), and the United Kingdom (12/464, 2.6%). Studies from other countries collectively accounted for 20.5% (n=95).

**Table 1:** General characteristics of included studies.

Characteristic				Values
**Publication year, n (%)**				
	2025		256 (55.2)	
	2024		188 (40.5)	
	2023		19 (4.1)	
	2022		1 (0.2)	
**Country, n (%)**				
	United States		153 (33.0)	
	China		94 (20.3)	
	Germany		37 (8.0)	
	Turkey		31 (6.7)	
	Italy		17 (3.7)	
	Israel		13 (2.8)	
	South Korea		12 (2.6)	
	United Kingdom		12 (2.6)	
	Other countries^a^		95 (20.5)	
**Model type, n (%)**				
	Existing model		356 (76.7)	
	Fine-tuning model + existing model		49 (10.6)	
	Fine-tuning model		40 (8.6)	
	Self-developed model		17 (3.7)	
	Self-developed model + existing model		2 (0.4)	
**Location of the large language model, n (%)**				
	Cloud deployment		72 (15.5)	
	Local deployment		70 (15.1)	
	External cloud service		69 (14.9)	
	Others^b^		8 (1.7)	
	Cannot judge		57 (12.3)	
	Not report		188 (40.5)	
**Task type, n (%)**				
	Making diagnoses		134 (28.9)	
	Clinical note-taking		62 (13.4)	
	Making treatment recommendations		45 (9.7)	
	Generating medical reports		43 (9.3)	
	Biomedical data mining		38 (8.2)	
	Prognostic predictive model		22 (4.7)	
	Communicating with patients		20 (4.3)	
	Making diagnoses + Making treatment recommendations		19 (4.1)	
	Other tasks^c^		81 (17.5)	
**Sample size^d^, n (%)**				
	**Patients (n=342, 73.7%)**			
		<100	125 (36.5)	
		100-1000	161 (47.1)	
		1000-10,000	35 (10.2)	
		≥10,000	19 (5.6)	
		Not report	2 (0.6)	
	**Token (n=16, 3.4%)**			
		100-1000	10 (62.5)	
		1000-10,000	3 (18.8)	
		≥10,000	2 (12.5)	
		Not report	1 (6.3)	
	**Notes (n=94, 20.3%)**			
		<100	12 (12.8)	
		100-1000	19 (20.2)	
		1000-10,000	23 (24.5)	
		≥10,000	38 (40.4)	
		Not report	2 (2.1)	
	**Images (n=12, 2.6%)**			
		<100	2 (16.7)	
		100-1000	7 (58.3)	
		1000-10,000	1 (8.3)	
		≥10,000	2 (16.7)	
**Number of centers^e^, n (%)**				
	1		377 (81.3)	
	2		41 (8.8)	
	3		16 (3.4)	
	4		7 (1.5)	
	≥5		20 (4.3)	
	Not report		3 (0.6)	
**Summarize the large language models used in the research, n (%)**				
	ChatGPT		341 (73.5)	
	Llama		74 (15.9)	
	Mistral		27 (5.8)	
	Flan		13 (2.8)	
	Claude		19 (4.1)	
	Gemini		32 (6.9)	
	Gemma		9 (1.9)	
	GLM		9 (1.9)	
	Deepseek		6 (1.3)	
	Qwen		13 (2.8)	
	Fine-tuning		49 (10.6)	
	Others		73 (15.7)	
**Type of data, n (%)**				
	Text		366 (78.9)	
	Text + Image		35 (7.5)	
	Text + Audio		4 (0.9)	
	Image		56 (12.1)	
	Audio		2 (0.4)	
	Text + Image + Audio		1 (0.2)	
**Data collection method, n (%)**				
	Retrospective		409 (88.1)	
	Prospective		53 (11.4)	
	Prospective + retrospective		2 (0.4)	
**Follow the statement, n (%)**				
	Not report		435 (93.8)	
	STROBE^f^		17 (3.7)	
	STARD^g^		3 (0.6)	
	TRIPOD^h^		7 (1.5)	
	CLAIM^i^		2 (0.4)	
**Registered, n (%)**				
	No		451 (97.2)	
	Yes		13 (2.8)	

^a^Other countries: Japan (n=11), Australia (n=10), France (n=9), Spain (n=9), Canada (n=6), Switzerland (n=5), India (n=5), Singapore (n=4), Belgium (n=3), Brazil (n=3), Croatia (n=3), the Netherlands (n=3), Pakistan (n=3), Saudi Arabia (n=3), Ireland (n=2), Mexico (n=2), Portugal (n=2), Romania (n=2), Thailand (n=2), Burkina Faso (n=1), Finland (n=1), Iran (n=1), Jordan (n=1), Poland (n=1), Ukraine (n=1), United Arab Emirates (n=1), and Vietnam (n=1).

^b^Other location of LLMs: local deployment + cloud deployment (n=5); local deployment + external cloud service (n=2); cloud deployment + external cloud service (n=1).

^c^Other tasks: synthesizing data for research (n=15), translation (n=12), triaging patients (n=11), conducting medical research (n=7), making diagnoses + triaging patients + Making treatment recommendations (n=5), making diagnoses + generating medical reports (n=4), generating billing codes (n=3), writing prescriptions (n=3), making diagnoses + triaging patients (n=2), making diagnoses + biomedical data mining (n=2); educating patients (n=2), making treatment recommendations + triaging patients (n=2), communicating with patients + making treatment recommendations (n=2), clinical note-taking + making treatment recommendations (n=2); enhancing medical knowledge (n=1), educating patients + making treatment recommendations + making diagnoses (n=1), communicating with patients + making diagnoses + making treatment recommendations (n=1), triaging patients + prognostic (n=1), generating medical reports + making treatment recommendations (n=1), generating medical reports + prognostic (n=1), clinical note-taking + generating medical reports (n=1), clinical note-taking + prognostic (n=1), and clinical note-taking + translation (n=1).

^d^Sample size was defined according to the primary data modality: number of patients (clinical studies), number of images (imaging studies), or number of clinical notes/documents (text-based studies), number of tokens (referring to the unit of original studies).

^e^Referring to the number of clinical sites contributing patient data.

^f^STROBE: Strengthening the Reporting of Observational Studies in Epidemiology.

^g^STARD: Standards for Reporting of Diagnostic Accuracy.

^h^TRIPOD: Transparent Reporting of a Multivariable Prediction Model for Individual Prognosis or Diagnosis.

^i^CLAIM: Checklist for Artificial Intelligence in Medical Imaging.

With respect to model type, 356 (76.7%) studies used existing models, 49 (10.6%) studies combined fine-tuning with existing models, 40 (8.6%) studies applied fine-tuning alone, 17 (3.7%) studies developed their own models, and 2 (0.4%) studies reported using both self-developed and existing models. Regarding the location of LLM deployment, 72 (15.5%) studies reported cloud deployment, 70 (15.1%) studies reported local deployment, and 69 (14.9%) studies reported the use of external cloud services. Eight (1.7%) studies used other deployment approaches, 57 (12.3%) studies could not be judged from the report, and 188 (40.5%) studies did not provide deployment information.

A total of 134 (28.9%) studies focused on making diagnoses, 62 (13.4%) studies focused on clinical note-taking, 45 (9.7%) studies focused on making treatment recommendations, 43 (9.3%) studies focused on generating medical reports, and 38 (8.2%) studies focused on biomedical data mining. Prognostic predictive model tasks were examined in 22 (4.7%) studies, and communication with patients in 20 (4.3%) studies. In addition, 19 (4.1%) studies combined diagnostic and treatment recommendation tasks, while other tasks were reported in 81 (17.5%) studies.

Regarding sample size, the distribution was varied: 125 (36.5%) studies enrolled fewer than 100 patients, 161 (47.1%) studies included 100-1000 patients, 35 (10.2%) studies included 1000-10,000 patients, 19 (5.6%) studies included more than 10,000 patients, and 2 (0.6%) studies did not report sample size. For token-based datasets (n=16), 10 (62.5%) studies used 100-1000 tokens, 3 (18.8%) studies used 1000-10,000, 2 (12.5%) studies used more than 10,000, and 1 (6.3%) study did not report. Regarding note-based datasets (n=94), 12 (12.8%) studies analyzed fewer than 100 notes, 19 (20.2%) studies used 100-1000, 23 (24.5%) studies used 1000-10,000, 38 (40.4%) studies used more than 10,000, and 2 (2.1%) studies did not report. For image-based datasets (n=12), 2 (16.7%) studies included fewer than 100 images, 7 (58.3%) studies included 100-1000, 1 (8.3%) studies included 1000-10,000, and 2 (16.7%) studies included more than 10,000.

Regarding the number of centers, 377 (81.3%) studies were conducted in a single center, 41 (8.8%) studies were conducted in 2 centers, 16 (3.4%) studies were conducted in 3 centers, and 7 (1.5%) studies were conducted in 4 centers. Twenty (4.3%) studies involved 5 or more centers, while 3 (0.6%) studies did not report this information. As for LLMs used in the research, ChatGPT was the most frequently used model, accounting for 341 (73.5%) studies. This was followed by 74 (15.9%) studies used Llama, 32 (6.9%) studies used Gemini, 27 (5.8%) studies used Mistral, 19 (4.1%) studies used Claude, and 13 (2.8%) studies used Qwen. Additionally, 49 (10.6%) studies applied fine-tuning techniques, while 73 (15.7%) studies reported using other models not specifically listed. Regarding data type, 366 (78.9%) studies used text; 56 (12.1%) studies used image data; 35 (7.5%) studies combined text and image; 4 (0.9%) studies combined text and audio; 2 (0.4%) studies used audio only; and 1 (0.2%) study combined text, image, and audio. With respect to data collection method, 409 studies (88.1%) were retrospective, 53 (11.4%) studies were prospective, and 2 (0.4%) studies combined both prospective and retrospective approaches.

With respect to reporting standards, 435 (93.8%) studies did not specify adherence to any guideline/statement, while 17 (3.7%) studies followed Strengthening the Reporting of Observational Studies in Epidemiology (STROBE) statement, 3 (0.6%) studies followed the Standards for Reporting of Diagnostic Accuracy (STARD) statement, 7 (1.5%) studies followed TRIPOD statement, and 2 (0.4%) studies followed Checklist for Artificial Intelligence in Medical Imaging. Regarding study registration, 451 (97.2%) studies were not registered, and only 13 (2.8%) studies reported registration.

**Figure 2:**
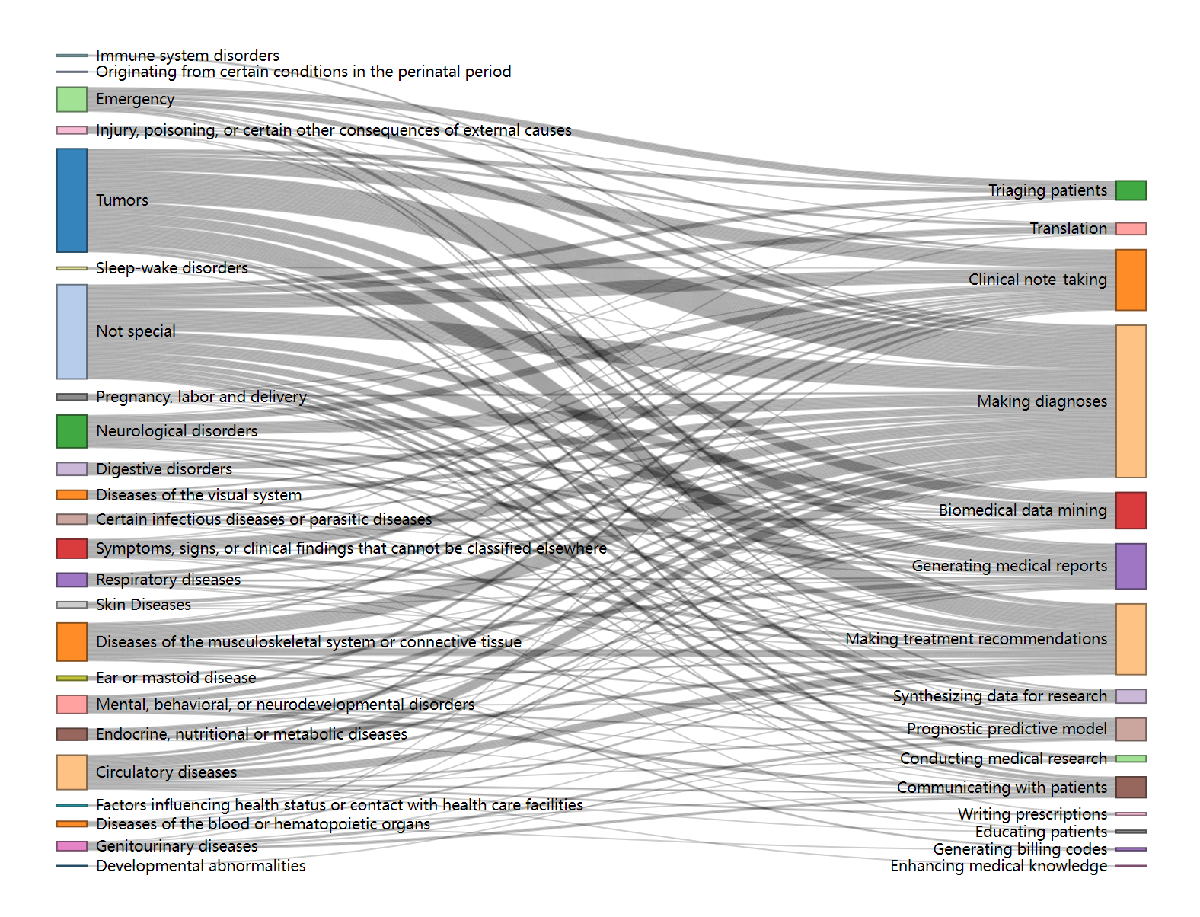
Sankey diagram of disease categories and task types.

The Sankey diagram (Figure 2) shows that the most frequent disease-task pairs included tumors with making diagnoses (n=35), tumors with making treatment recommendations (n=23), and tumors with clinical note-taking (n=16). In addition, studies categorized as “not special” also frequently addressed making diagnoses (n=24) and clinical note-taking (n=15). Other notable pairs included musculoskeletal disorders with making diagnoses (n=17), neurological disorders with making diagnoses (n=13), and circulatory diseases with making diagnoses (n=11). Tasks such as generating medical reports and biomedical data mining were also commonly associated with tumors and “not special” categories. The summary table could be found in Multimedia Appendix 3.

### Characteristics of Privacy Protection

Table 2 outlines the characteristics of privacy protection measures implemented in the included studies. Regarding ethical oversight, 419 (90.3%) studies reported approval from an ethics committee, whereas 45 (9.7%) studies did not. With respect to patient consent, 224 (48.3%) studies reported a waiver of informed consent, 92 (19.8%) studies indicated that informed consent had been obtained, and 148 (31.9%) studies did not report consent information. For data availability, 203 (43.8%) studies did not provide a statement, 160 (34.5%) studies declared that data were available from the corresponding author upon reasonable request, 66 (14.2%) studies stated that data were not available, and 35 (7.5%) studies made data publicly accessible.

**Table 2:** Characteristics of privacy protection. Our categorization of privacy protection methods is based on terminology as reported by the original studies. However, the definitions of “de-identification” and “anonymization” vary across contexts; thus, the risk implications should be interpreted with caution.

Characteristics			Values, n (%)
**Ethical review**			
	Yes		419 (90.3)
	No		45 (9.7)
**Patient consent**			
	Waiver of informed consent		224 (48.3)
	Not report		148 (31.9)
	Informed consent has been obtained		92 (19.8)
**Data availability declaration**			
	Not report		203 (43.8)
	Corresponding author on reasonable request		160 (34.5)
	Not open		66 (14.2)
	Public		35 (7.5)
**Privacy protection technology**			
	Not report		178 (38.4)
	**Deidentification**		158 (34.1)
		Cannot judge from report	116 (73.4)
		Based on manual	17 (10.8)
		Based on rule matching	13 (8.2)
		Others^a^	12 (7.6)
	Anonymization		91 (19.6)
	Deidentification+Anonymization		23 (5.0)
	Others^b^		14 (3.0)
**Is there a statement to remove any personally identifiable information?**			
	No		363 (78.2)
	Yes		101 (21.8)
**Were direct identifiers or indirect identifiers removed?**			
	Direct identifiers		166 (35.8)
	Indirect identifiers		9 (1.9)
	Cannot judge		107 (23.1)
	Not report		182 (39.2)
**Whether the degree of deidentification is assessed?**			
	No		458 (98.7)
	Yes		6 (1.3)
**Reidentification protection technology used?**			
	No		455 (98.1)
	Yes		9 (1.9)
**Declaration of compliance with safety standards**			
	Health Insurance Portability and Accountability Act		44 (9.5)
	General Data Protection Regulation		6 (1.3)
	Both		2 (0.4)
	Not report		412 (88.8)

^a^Based on rule matching + machine learning + deep learning (n=3), based on LLMs (n=2), based on rule matching + manual (n=2), based on rule matching + machine learning (n=1), based on synthetic data (n=1), based on postprocessing (n=1), based on machine learning (n=1), and based on deep learning+ postprocessing (n=1).

^b^Data hosting (n=5), anonymization + data hosting (n=3), federated learning (n=1), anonymization + data hosting + homomorphic encryption (n=1), anonymization + homomorphic encryption (n=1), deidentification + data hosting (n=1), data augmentation (likely referred to synthetic data generation; n=1), and homomorphic encryption (n=1).

When examining privacy protection technologies, 178 (38.4%) studies did not report the methods used, while 158 (34.1%) studies applied deidentification, 91 (19.6%) studies used anonymization, 23 (5.0%) studies reported combining both, and 14 (3.0%) studies used other technologies. Among those providing more detail about deidentification, 17 (10.8%) studies used manual methods, 13 (8.2%) studies applied rule-based matching, 12 (7.6%) studies reported other approaches, and 116 (73.4%) studies cannot be judged from the reports.

Concerning statements on the removal of personally identifiable information, 363 (78.2%) studies did not provide such a statement, while 101 (21.8%) studies explicitly reported it. About the type of identifiers removed, 166 (35.8%) studies specified the removal of direct identifiers, 9 (1.9%) studies reported the removal of indirect identifiers, 107 (23.1%) studies could not be judged from the report, and 182 (39.2%) studies did not provide this information. Regarding assessment of the degree of deidentification, 458 (98.7%) studies did not report such an assessment, while 6 (1.3%) studies did. Concerning reidentification protection technologies, 455 (98.1%) studies did not use them, and 9 (1.9%) studies reported their use. With respect to compliance with safety standards, 44 (9.5%) studies declared adherence to HIPAA, 6 (1.3%) studies to the General Data Protection Regulation, and 2 (0.4%) studies to both, whereas 412 (88.8%) studies did not provide such information.

Table 3 shows the characteristics of privacy protection technology for different data types. Among text-based studies (n=366), 131 (35.8%) studies applied deidentification, 74 (20.2%) studies used anonymization, 15 (4.1%) studies combined both, 4 (1.1%) studies reported data hosting, 2 (0.5%) studies reported anonymization with data hosting, 6 (1.6%) studies used other methods, and 134 (36.6%) studies did not report. For image-based studies (n=56), 12 (21.4%) studies reported deidentification, 10 (17.9%) studies reported anonymization, 5 (8.9%) studies reported both, 1 (1.8%) study reported anonymization with data hosting, 1 (1.8%) study reported data hosting, and 27 (48.2%) studies did not report. For audio-based studies (n=2), one (50.0%) study reported anonymization and one (50.0%) study did not. For combined text and image studies (n=35), 14 (40.0%) studies used deidentification, 6 (17.1%) studies used anonymization, 3 (8.6%) studies used both, and 12 (34.3%) studies did not report. For text and audio studies (n=4), one (25.0%) study reported deidentification, and 3 (75.0%) studies did not. The single study using text, image, and audio data did not report its privacy protection method.

**Table 3:** Characteristics of privacy protection technology for different data types.

Type of data and privacy protection technology^a^		Values, n (%)
**Text (n=366)**		
	Deidentification	131 (35.8)
	Anonymization	74 (20.2)
	Deidentification + anonymization	15 (4.1)
	Data hosting	4 (1.1)
	Anonymization + data hosting	2 (0.5)
	Others^b^	6 (1.6)
	Not reported	134 (36.6)
**Images (n=56)**		
	Deidentification	12 (21.4)
	Anonymization	10 (17.9)
	Deidentification + anonymization	5 (8.9)
	Anonymization + data hosting	1 (1.8)
	Data hosting	1 (1.8)
	Not reported	27 (48.2)
**Audio (n=2)**		
	Anonymization	1 (50.0)
	Not reported	1 (50.0)
**Text**+**Images (n=35)**		
	Deidentification	14 (40.0)
	Anonymization	6 (17.1)
	Deidentification + anonymization	3 (8.6)
	Not reported	12 (34.3)
**Text+Audio (n=4)**		
	Deidentification	1 (25.0)
	Not reported	3 (75.0)
**Text** + **Images** + **Audio (n=1)**		
	Not reported	1 (100.0)

^a^Classify according to the conditions reported in the original articles of the included studies.

^b^Anonymization + data hosting + homomorphic encryption (n=1), anonymization + homomorphic encryption (n=1), federated learning (n=1), data augmentation (n=1), deidentification + data hosting (n=1), and homomorphic encryption (n=1).

## Discussion

### Main Findings

In this scoping review, we identified 464 studies published between 2022 and 2025 that focus on the development and application of LLMs in health care using PHI. Strikingly, 256 (55.2%) of these studies were published in 2025 alone, compared with 188 (40.5%) studies in 2024, 19 (4.1%) studies in 2023, and only one (0.2%) study in 2022. This sharp increase highlights the extremely rapid pace of research in this emerging field and reflects the growing recognition of both the opportunities and challenges associated with LLM deployment in health care. These studies encompass a variety of countries, disease domains, and task types. Overall, the ethical reviews of these studies have been largely satisfactory. The vast majority of studies have reported on the approval of the ethics committee, ensuring that their procedures meet the relevant ethical standards. Nevertheless, there remains a shortfall in reporting on informed consent in certain prospective studies. It is concerning that a small number of LLM research projects using imaging data or retrospective data from electronic medical records fail to adequately report ethical review processes and the consideration of patient-informed consent, including whether consent was obtained or formally waived. Even when research involves only patient imaging data or retrospective data, it must still undergo rigorous ethical review [31,32]. Strict adherence to ethical review processes is essential to ensure the fairness and scientific integrity of medical research and to safeguard patient rights.

According to our findings, more than half of LLMs use cloud deployments, and the generative nature of LLMs can accidentally expose private data learned during training [33], including PII [16]. And the vast parameter counts and extensive training corpora of LLMs enlarge their memorization footprint, leakage vectors, and prompt-manipulation surface, thereby exposing critical vulnerabilities that render these models prime targets for prompt-injection and data-poisoning attacks. Therefore, it is necessary to enhance the privacy protection of PHI. Cloud deployment offers the advantages of cost-effectiveness and scalability, as users are not required to invest in expensive hardware and can dynamically scale computing resources as needed. Additionally, cloud services provide established tools and global accessibility, facilitating rapid iteration and collaboration among distributed teams. However, cloud deployments also face significant data privacy risks, as data must be uploaded to third-party servers, which can lead to risks such as data breaches, unauthorized access, compliance issues, service disruptions, and model exploitation. These risks can be effectively mitigated through the integration of technical countermeasures like zero-trust architecture [34], edge computing [35], data encryption [36], and access control.

Given the inherently high sensitivity of medical data and the significant risk of irreversible harm upon disclosure, the development of privacy-preserving safeguards for LLMs has become imperative. This requirement is not merely a technical prerequisite but also reflects an uncompromising mandate at the ethical and regulatory levels. In current research on the application of LLMs to health care, approximately one-third of the studies fail to mention any techniques for effectively protecting PHI. PHI includes PII such as patient names, addresses, Social Security numbers, and medical record numbers. Once compromised, such information can lead to serious privacy breaches and security risks. Therefore, removing PII is the first crucial step in safeguarding patient privacy [37,38]. By effectively eliminating or obfuscating these direct identifiers, it becomes possible to mitigate the risk of unauthorized access to PHI and thereby reduce the negative consequences of potential data breaches. The failure to prioritize PII protection in the deployment of LLMs within health care poses significant risks that extend beyond immediate privacy concerns. It threatens the integrity of patient-provider relationships, exposes individuals to financial and identity-related crimes, stifles technological and scientific progress, and raises critical ethical issues. Among the included studies, only one mentioned the use of federated learning techniques, and none used homomorphic encryption. Despite this, federated learning and homomorphic encryption are emerging as pivotal techniques for privacy-preserving in LLMs. Federated learning, due to its distributed training and decentralized model architecture, has gained significant traction in health care [39]. Future research should prioritize the development of comprehensive data privacy protection to facilitate the broader adoption of federated learning and homomorphic encryption in health care. Synthetic data generation has emerged as a promising solution to address privacy concerns in health care research and LLMs [40]. This approach uses AI models to create realistic, anonymized patient data that preserves privacy while enabling data access for secondary purposes [41]. While synthetic data offers benefits in promoting privacy, equity, safety, and continual learning, challenges remain, including the potential introduction of flaws and biases. Further research is needed to develop unified quality assessment metrics and address the current deficiency in longitudinal synthetic data generation [40].

In studies involving PHI deidentification techniques or the use of anonymized data, the related descriptions are often very vague. Although these studies mention the removal of PII or the use of anonymized data, they typically do not specify which PII elements were removed or merely use ambiguous phrases such as “removing PII” or “using anonymized data,” lacking detailed technical explanations and transparency. First, the absence of clear technical descriptions undermines the credibility and reproducibility of the research, making it difficult for other researchers to replicate the results under the same conditions and thereby affecting the scientific validity and the effectiveness of subsequent applications. Second, privacy protection may be at risk because the lack of transparency in the deidentification process can result in PII not being fully removed, increasing the risk of data breaches. Finally, ethical issues arise as well. If the data processing for deidentification and anonymization is not sufficiently transparent, it can lead to ethical disputes regarding privacy protection and informed consent, especially when assessing whether there remains a risk of reidentifying individuals from the data. This lack of transparency may result in failing to meet the requirements of ethical reviews. According to the Food and Drug Administration, even deidentified data must retain traceability to meet regulatory requirements. Traceability after deidentification is not only a critical component of privacy protection but also essential for ensuring data availability, compliance, and credibility. It plays an indispensable role in data sharing, research transparency, emergency response, and other areas, providing a robust foundation for data-driven decision-making and innovation. When designing and implementing deidentification schemes, the need for traceability must be carefully considered to achieve a balance between privacy protection and data utility. In addition, the descriptions of “de-identification assessment” provided in current studies often lack transparency, leaving their scope and rigor unclear. For instance, it remains uncertain whether such assessments involved quantitative estimation of reidentification risk, reference to regulatory standards such as HIPAA Safe Harbor, or validation by external independent experts. The absence or ambiguity of these elements makes it difficult to determine the effectiveness and compliance of deidentification practices. Future studies should explicitly report these aspects in both methodology and results to ensure greater rigor and credibility in protecting patient privacy.

The HIPAA establishes standards and practices for deidentifying PHI. According to this rule, there are 2 methods for deidentifying PHI: expert determination, which requires a formal assessment by a qualified expert; and Safe Harbor, which involves the removal of specified identifiers so that covered entities and business associates cannot identify individuals from the remaining information. Although HIPAA does not explicitly use the term “anonymization,” anonymization is often considered an irreversible process that ensures data can no longer identify individuals. Anonymization requirements are more stringent than deidentification, as they guarantee that the data cannot be reidentified under any circumstances. While this study retains the original terminology used in the articles reviewed, most studies are vague in their descriptions of deidentification and anonymization, making it difficult to determine which specific methods were used. Researchers should clearly specify the approaches used to protect data privacy to ensure transparency and accuracy.

Medical big data exhibits unique multimodal characteristics. The term “multimodal” refers to the diverse sources and forms of medical data, which include laboratory data (eg, laboratory results), imaging data (eg, computed tomographic scans, x-rays, ultrasounds, and electrocardiograms), and video data containing audio (eg, fetal ultrasounds). Depending on the specific data type, its confidentiality, integrity, and availability are ensured through various methods. In our research, we found that current medical data privacy protection primarily relies on deidentification and anonymization techniques. However, in the context of multimodal medical data, a single privacy protection method is often insufficient to effectively prevent data leakage, tampering, and misuse. Therefore, designing multimodal data privacy protection technologies represents a critical direction for future research.

### Strengths and Limitations

While existing literature reviews predominantly focus on the applications of LLMs in health care, there remains a notable gap in comprehensive scoping reviews that specifically evaluate privacy protection measures for PHI within LLM implementations (PHI-LLMs). Previous analyses addressing privacy concerns have primarily examined broader contexts rather than focusing specifically on patient information protection in language model applications, resulting in insufficient coverage of both technical safeguards and systemic compliance aspects within health care ecosystems.

A limitation of this study is that the evaluation of privacy protection measures relies solely on the information reported in published papers. Therefore, if certain studies have implemented privacy protection methods but did not disclose them in detail within their articles, we are unable to identify them. This situation may affect the comprehensiveness and accuracy of our evaluation. While we catalogued the adoption of different privacy protection methods, our review did not evaluate their security levels, implementation quality, or practical trade-offs. Future research should systematically assess the effectiveness and applicability of these techniques in health care-specific settings.

### Implications for Research and Practice

Based on the 3 key findings outlined above, we offer the following additional recommendations for protecting patient privacy in health care–related LLM research, structured around 3 phases: study design, implementation, and reporting. When conducting research reports, the key terms can be referred to for a standardized design. The glossary of key terms (Textbox 2) can be found in the Multimedia Appendix 3.

**Glossary of key terms.**

- Protected health information: Individually identifiable health information relating to health status, care, or payment that is protected under the Health Insurance Portability and Accountability Act.
- Personally identifiable information: It refers to any information that can directly or indirectly identify a specific individual, including name, ID number, address, contact information, and data that can identify identity when combined with other information. Personally identifiable information is a broad concept that encompasses all data that can identify an individual.
- Deidentification (Safe Harbor, Health Insurance Portability and Accountability Act): Removal of 18 specified identifiers (eg, name, address, phone number, and dates) such that the data is no longer considered protected health information.
- Deidentification (Ontario Guidance): A risk-based statistical approach that quantifies reidentification risk and determines whether it is acceptably small.
- Anonymization (General Data Protection Regulation): Data is processed in such a way that reidentification is no longer possible by any means “reasonably likely to be used.”
- Rule-based matching: Algorithmic detection and removal of direct identifiers (eg, names, addresses) using predefined rules or dictionaries.
- Federated learning: A decentralized machine learning paradigm where models are trained collaboratively without exchanging raw data.
- Reidentification protection technology: Technical and organizational measures (eg, k-anonymity and differential privacy) that reduce the likelihood of reidentifying individuals, acknowledging that zero risk is unattainable but very low residual risk is acceptable under most regulations.
- Direct identifiers: Variables that can uniquely and directly identify an individual, such as name, social security number, phone number, full address, and medical record number.
- Indirect identifiers (quasi-identifiers): Variables that cannot identify an individual alone but may enable reidentification when combined with other data, such as age, gender, zip code, and admission date.

### Considerations in Research Design

In the design phase of LLM research, patient privacy protection must be prioritized. First, the data minimization principle should be strictly adhered to, meaning only the minimum necessary PHI required to achieve the research objectives should be collected and used [42]. Second, a clear definition of research purpose and usage scope is essential, ensuring that all PII used has a well-defined purpose and is not repurposed for unauthorized studies or commercial applications. Additionally, ethical approval and informed consent are critical components of the design phase. Researchers must submit detailed research plans to an Institutional Review Board or Ethics Committee, outlining how PII will be obtained, used, and protected. Where applicable, obtaining informed consent from patients is necessary to ensure they are aware of how their data will be used and safeguarded.

### Considerations in Research Implementation

In the implementation phase, priority should be given to deploying the LLM locally. During model training, multiple patient privacy protection strategies such as deidentification, anonymization, federated learning, synthetic data, and differential privacy should be used. Due to the potential risk of reidentification, continuous security monitoring and auditing are indispensable. The research team should conduct regular security assessments and vulnerability scans to promptly identify and address potential security vulnerabilities. Postbreach responses also constitute an indispensable part of a comprehensive privacy protection framework. Effective incident response should include rapid detection, containment, patient notification, and remediation strategies, which are increasingly emphasized in health care data governance guidelines.

### Considerations in Research Reporting

Research reports should fully embody the principles of transparency and reproducibility. Researchers should disclose in detail the data sources, ethical approval processes, informed consent procedures, and privacy protection techniques used. Selecting appropriate reporting guidelines (such as STROBE [43], STARD [44], CONSORT-AI [Consolidated Standards of Reporting Trials–Artificial Intelligence] [45], and TRIPOD-LLM [19]) can improve report quality and provide a reference for other researchers.

### Conclusions

Our scoping review sounds an alarm on the inadequately addressed imperative of patient privacy protection in medical research using LLMs. In response, we formulate comprehensive recommendations for the study design, implementation, and reporting phases to fortify PHI protection and foster transparency in PHI-LLM research. Our findings compellingly argue for the urgent development of stricter regulatory frameworks and the integration of advanced privacy-preserving technologies to safeguard PHI. It is anticipated that such measures will enable future health care applications of LLMs to achieve a balance between innovation and rigorous patient privacy protection, thus elevating ethical standards and scientific credibility.

## Data Availability

The data generated during the scoping review are available from the corresponding author on reasonable request. However, the majority of such data have been presented on paper in tables, figures, and text.

## Appendix

Multimedia Appendix 1 PRISMA-ScR checklist.

Multimedia Appendix 2 Search strategy for scoping review.

Multimedia Appendix 3 The specific references included in the analysis.

## Abbreviations

CONSORT-AI: Consolidated Standards of Reporting Trials–Artificial Intelligence
HIPAA: Health Insurance Portability and Accountability Act
LLM: large language model
PHI: patient health information
PII: personally identifiable information
PRISMA-ScR: Preferred Reporting Items for Systematic Reviews and Meta-Analyses Extension for Scoping Reviews
STARD: Standards for Reporting of Diagnostic Accuracy
STROBE: Strengthening the Reporting of Observational Studies in Epidemiology
TRIPOD: Transparent Reporting of a Multivariable Prediction Model for Individual Prognosis or Diagnosis
