# Supplementary material for: Considerations for Patient Privacy of Large Language Models in Health Care: Scoping Review
Source: J Med Internet Res. 2025 Nov 21;27:e76571. doi: 10.2196/76571 (PMC12680930; doi:10.2196/76571)
Supplement: Multimedia Appendix 2 [file jmir_v27i1e76571_app2.docx]

**Multimedia Appendix 1 Search strategy for scoping review.**

**PubMed Search date 2025/07/20**

Results： 5535

#1 ("Generative AI"[Title/Abstract] OR "large language model"[Title/Abstract] OR "large language models"[Title/Abstract] OR "ChatGPT"[Title/Abstract] OR "GPT-4"[Title/Abstract] OR "GPT-3"[Title/Abstract])

#2 ("healthcare"[Title/Abstract] OR "health care"[Title/Abstract] OR "clinical"[Title/Abstract] OR "medicine"[Title/Abstract] OR "medical"[Title/Abstract] OR "diagnosis"[Title/Abstract] OR "treatment"[Title/Abstract] OR "clinical decision support"[Title/Abstract] OR "clinical research"[Title/Abstract] OR "electronic health record"[Title/Abstract] OR "electronic health records"[MeSH Terms])

#3 #1 AND #2

#4 #3 AND (2022/01/01:2025/07/20[Date - Publication] AND "English"[Language])

#5 #4 NOT ("review"[Publication Type] OR "systematic review"[Publication Type] OR "meta-analysis"[Publication Type] OR "comment"[Publication Type] OR "editorial"[Publication Type] OR "letter"[Publication Type] OR "news"[Publication Type] OR "Preprint"[Publication Type])

**Embase Search date 2025/07/20**

Results: 4288

#1 ('generative ai':ti,ab OR 'large language model*':ti,ab OR 'chatgpt':ti,ab OR 'gpt-4':ti,ab OR 'gpt-3':ti,ab)

#2 ('healthcare':ti,ab OR 'health care':ti,ab OR 'clinical':ti,ab OR 'medicine':ti,ab OR 'medical':ti,ab OR 'diagnosis':ti,ab OR 'treatment':ti,ab OR 'clinical decision support':ti,ab OR 'clinical research':ti,ab OR 'electronic health record*':ti,ab OR 'electronic health record'/exp)

#3 #1 AND #2

#4 #3 AND [english]/lim AND [2022-2025]/py

#5 #4 NOT ('review'/it OR 'systematic review'/it OR 'meta analysis'/it OR 'editorial'/it OR 'letter'/it OR 'note'/it OR 'conference abstract'/it OR 'preprint'/it)

.......................
