# Supplementary material for: Considerations for Patient Privacy of Large Language Models in Health Care: Scoping Review
Source: J Med Internet Res. 2025 Nov 21;27:e76571. doi: 10.2196/76571 (PMC12680930; doi:10.2196/76571)
Supplement: Multimedia Appendix 3 [file jmir_v27i1e76571_app3.docx]

**Multimedia Appendix 2**

**1.Summary of the top 10 disease–task pair frequencies**

| Disease | Task | Frequency |
| --- | --- | --- |
| Tumors | Making diagnoses | 35 |
| Not special | Making diagnoses | 24 |
| Tumors | Making treatment recommendations | 23 |
| Diseases of the musculoskeletal system or connective tissue | Making diagnoses | 17 |
| Tumors | Clinical note-taking | 16 |
| Not special | Clinical note-taking | 15 |
| Neurological disorders | Making diagnoses | 13 |
| Not special | Generating medical reports | 13 |
| Symptoms, signs, or clinical findings that cannot be classified elsewhere | Making diagnoses | 12 |
| Tumors | Biomedical data mining | 12 |

**2.Definitions and example of LLMs’ task type**

| Task Type | Definition | Example |
| --- | --- | --- |
| Clinical note-taking | The process of recording detailed information about a patient’s health status, medical history, symptoms, physical examination findings, diagnostic test results, treatment plans, typically documented in the patient’s EMR. | Develop a clinical generative large language model, GatorTronGPT, for biomedical natural language processing, clinical text generation, and healthcare text evaluation[1]. |
| Making diagnoses | The process of identifying the nature or cause of a disease or condition through the examination of symptoms, medical history,  and diagnostic tests. | To develop an interactive dermatology diagnostic system based on multimodal large language models[2]. |
| Triaging patients | Clinical triage is the process of prioritizing patients based on the severity of their condition and the urgency of their need for care. | To assess the efficacy of LLMs and the associated product ChatGPT in ED triage compared to personnel of varying training status and to investigate if the models' responses can enhance the triage proficiency of untrained personnel[3]. |
| Conducting medical research | Medical research generation, including writing papers, refers to the process of conducting original research in medicine or health care and documenting the findings in academic papers. | To evaluating chatgpt's role in supporting military readiness assessment[4]. |
| Biomedical data mining | The process of searching and extracting data regarding a patient’s health. | To develop a comprehensive sepsis knowledge graph by leveraging the capabilities of LLMs, specifically GPT-4.0, in conjunction with multicenter clinical databases[5]. |
| Generating medical reports | An image-captioning task of producing a professional report according to input image data. | Our purpose is to assess the accuracy and reproducibility of ChatGPT, a large language model, in generating structured thyroid ultrasound reports[6]. |
| Synthesizing data for research | Data synthesis refers to the process of combining and analyzing data from multiple sources to generate new insights, draw conclusions, or develop a comprehensive understanding of a topic. | To assess the feasibility and impact of the implementation of ChatGLM for RWD extraction within a Chinese hospital setting[7]. |
| Communicating with patients | The exchange of information between health care clinicians and patients. This could be done via patient messaging platforms or via chatbots integrated into the clinician workflow. | The study used large language models (LLMs) to develop an automated interpretation pipeline for both report generation and medical question-answering (QA) for FFA image[8]. |
| Carrying out a literature review | A literature review is a critical summary and evaluation of existing research or literature on a specific topic. | N/A |
| Making treatment recommendations | The process of providing treatment recommendations for patients to manage or cure their health conditions. | The study aimed to investigate the performance of Generative Pre-trained Transformer 4 (GPT-4), a large language model, for report-based medical decision-making in the context of cardiac MRI for suspected myocarditis[9]. |
| Gererating biling code | Medical billing is the process of submitting and following up on claims with health insurance companies to receive payment for health care services provided to patients. | Gemini, a publicly available large language model chatbot, was queried with  139 de-identified patient encounters from a single surgeon and asked to provide the Current Procedural Terminology code  based on the criteria for different encounter types[10]. |
| Translation | The process of converting medical texts, data, or information from one language to another. | To explore the capabilities of Chat Generative Pre-trained Transformer (ChatGPT) for the purpose of simplifying and translating radiology reports into Spanish, Hindi, and Russian languages, with comparisons to its performance in simplifying to the English language[11]. |
| Enhancing medical knowledge | The process of enhancing the skills, knowledge, and capabilities of health care professionals to meet the evolving needs of health care delivery. | This study explores the use of an advanced artificial intelligence program, the Generative Pre-trained Transformer (GPT), to enter pediatric stroke data into the IPSS[12]. |
| Educating patients | Providing patients with information and resources to help them understand their health conditions and treatment options for more  informed decision-making around their care. | To evaluate the potential of large language  models (LLMs) in health education for patients with  ankylosing spondylitis (AS)/spondyloarthritis (SpA),  focusing on the accuracy of information transmission,  patient acceptance and performance differences between different models[13]. |
| Writing prescription | The process by which a health care clinician, typically a physician or other qualified medical professional, orders medications or treatments for a patient. | The performance of intelligent tools such as ChatGPT 4.0 and Intelligent Health Promotion Systems (IHPS) in issuing exercise prescriptions for patients with hypertension comorbidities remains to be verified[14]. |
| Prognostic predictive model | Predictive of the probable future status or survival of a patient. | This study aimed to examine the performance of LLMs in predicting six postoperative risks using various fine-tuning strategies[15]. |

1. **Glossary of key terms.**
2. PHI (Protected Health Information): Individually identifiable health information relating to health status, care, or payment that is protected under HIPAA.
3. PII (Personally Identifiable Information): It refers to any information that can directly or indirectly identify a specific individual, including name, ID number, address, contact information, and data that can identify identity when combined with other information. PII is a broad concept that encompasses all data that can identify an individual.
4. Deidentification (Safe Harbor, HIPAA): Removal of 18 specified identifiers (eg, name, address, phone number, and dates) such that the data is no longer considered PHI.
5. Deidentification (Ontario Guidance): A risk-based statistical approach that quantifies reidentification risk and determines whether it is acceptably small.
6. Anonymization (GDPR): Data is processed in such a way that reidentification is no longer possible by any means “reasonably likely to be used.”
7. Rule-based matching: Algorithmic detection and removal of direct identifiers (eg, names, addresses) using predefined rules or dictionaries.
8. Federated learning: A decentralized machine learning paradigm where models are trained collaboratively without exchanging raw data.
9. Reidentification protection technology: Technical and organizational measures (eg, k-anonymity and differential privacy) that reduce the likelihood of reidentifying individuals, acknowledging that zero risk is unattainable but very low residual risk is acceptable under most regulations.
10. Direct identifiers: Variables that can uniquely and directly identify an individual, such as name, social security number, phone number, full address, and medical record number.
11. Indirect identifiers (quasi-identifiers): Variables that cannot identify an individual alone but may enable reidentification when combined with other data, such as age, gender, zip code, and admission date.

**4.The list of included studies[1-464]**

1. Peng C, Yang X, Chen A, Smith KE, PourNejatian N, Costa AB, et al. A study of generative large language model for medical research and healthcare. NPJ Digit Med. 2023 Nov 16;6(1):210. PMID: 37973919. doi: 10.1038/s41746-023-00958-w.

2. Zhou J, He X, Sun L, Xu J, Chen X, Chu Y, et al. Pre-trained multimodal large language model enhances dermatological diagnosis using SkinGPT-4. Nat Commun. 2024 Jul 5;15(1):5649. PMID: 38969632. doi: 10.1038/s41467-024-50043-3.

3. Masanneck L, Schmidt L, Seifert A, Kolsche T, Huntemann N, Jansen R, et al. Triage Performance Across Large Language Models, ChatGPT, and Untrained Doctors in Emergency Medicine: Comparative Study. J Med Internet Res. 2024 Jun 14;26:e53297. PMID: 38875696. doi: 10.2196/53297.

4. Tyburski AM, Garin ESP, Fox CJP. Evaluating ChatGPT's Role in Supporting Military Readiness Assessment. Military medicine. 2025 May 5. PMID: 40327326. doi: 10.1093/milmed/usaf161.

5. Yang H, Li J, Zhang C, Sierra AP, Shen B. Large Language Model-Driven Knowledge Graph Construction in Sepsis Care Using Multicenter Clinical Databases: Development and Usability Study. J Med Internet Res. 2025 Mar 27;27:e65537. PMID: 40146985. doi: 10.2196/65537.

6. Jiang H, Xia S, Yang Y, Xu J, Hua Q, Mei Z, et al. Transforming free-text radiology reports into structured reports using ChatGPT: A study on thyroid ultrasonography. Eur J Radiol. 2024 Jun;175:111458. PMID: 38613868. doi: 10.1016/j.ejrad.2024.111458.

7. Wang B, Lai J, Cao H, Jin F, Li Q, Tang M, et al. Enhancing the interoperability and transparency of real-world data extraction in clinical research: evaluating the feasibility and impact of a ChatGLM implementation in Chinese hospital settings. European heart journal Digital health. 2024 Nov;5(6):712-24. PMID: 39563908. doi: 10.1093/ehjdh/ztae066.

8. Chen X, Zhang W, Xu P, Zhao Z, Zheng Y, Shi D, et al. FFA-GPT: an automated pipeline for fundus fluorescein angiography interpretation and question-answer. NPJ Digit Med. 2024 May 3;7(1):111. PMID: 38702471. doi: 10.1038/s41746-024-01101-z.

9. Kaya K, Gietzen C, Hahnfeldt R, Zoubi M, Emrich T, Halfmann MC, et al. Generative Pre-trained Transformer 4 analysis of cardiovascular magnetic resonance reports in suspected myocarditis: A multicenter study. Journal of cardiovascular magnetic resonance : official journal of the Society for Cardiovascular Magnetic Resonance. 2024 Winter;26(2):101068. PMID: 39079602. doi: 10.1016/j.jocmr.2024.101068.

10. Latario LD, Fowler JR. Chatbot Demonstrates Moderate Interrater Reliability in Billing for Hand Surgery Clinic Encounters. Hand (New York, NY). 2024 Nov 16:15589447241295328. PMID: 39548885. doi: 10.1177/15589447241295328.

11. Gulati V, Roy SG, Moawad A, Garcia D, Babu A, Poot JD, et al. Transcending Language Barriers: Can ChatGPT Be the Key to Enhancing Multilingual Accessibility in Health Care? Journal of the American College of Radiology : JACR. 2024 Dec;21(12):1888-95. PMID: 38880289. doi: 10.1016/j.jacr.2024.05.009.

12. Fiedler AK, Zhang K, Lal TS, Jiang X, Fraser SM. Generative Pre-trained Transformer for Pediatric Stroke Research: A Pilot Study. Pediatric neurology. 2024 Nov;160:54-9. PMID: 39191085. doi: 10.1016/j.pediatrneurol.2024.07.001.

13. Ren Y, Kang YN, Cao SY, Meng F, Zhang J, Liao R, et al. Evaluating the performance of large language models in health education for patients with ankylosing spondylitis/spondyloarthritis: a cross-sectional, single-blind study in China. BMJ Open. 2025 Mar 21;15(3):e097528. PMID: 40118477. doi: 10.1136/bmjopen-2024-097528.

14. Xu Y, Liu Q, Pang J, Zeng C, Ma X, Li P, et al. Assessment of Personalized Exercise Prescriptions Issued by ChatGPT 4.0 and Intelligent Health Promotion Systems for Patients with Hypertension Comorbidities Based on the Transtheoretical Model: A Comparative Analysis. Journal of multidisciplinary healthcare. 2024;17:5063-78. PMID: 39539514. doi: 10.2147/JMDH.S477452.

15. Alba C, Xue B, Abraham J, Kannampallil T, Lu C. The foundational capabilities of large language models in predicting postoperative risks using clinical notes. NPJ Digit Med. 2025 Feb 11;8(1):95. PMID: 39934379. doi: 10.1038/s41746-025-01489-2.

16. Glicksberg BS, Timsina P, Patel D, Sawant A, Vaid A, Raut G, et al. Evaluating the accuracy of a state-of-the-art large language model for prediction of admissions from the emergency room. Journal of the American Medical Informatics Association : JAMIA. 2024 Sep 1;31(9):1921-8. PMID: 38771093. doi: 10.1093/jamia/ocae103.

17. Jiang Z, Cai X, Yang L, Gao D, Zhao W, Han J, et al. Learning to Summarize Chinese Radiology Findings With a Pre-Trained Encoder. IEEE transactions on bio-medical engineering. 2023 Dec;70(12):3277-87. PMID: 37314905. doi: 10.1109/TBME.2023.3280987.

18. Siepmann RM, Baldini G, Schmidt CS, Truhn D, Müller-Franzes GA, Dada A, et al. An automated information extraction model for unstructured discharge letters using large language models and GPT-4. Healthcare Analytics. 2025;7. doi: 10.1016/j.health.2024.100378.

19. Atkinson CJ, Seth I, Seifman MA, Rozen WM, Cuomo R. Enhancing Hand Fracture Care: A Prospective Study of Artificial Intelligence Application With ChatGPT. Journal of hand surgery global online. 2024 Jul;6(4):524-8. PMID: 39166196. doi: 10.1016/j.jhsg.2024.03.014.

20. Song X, Wang J, He F, Yin W, Ma W, Wu J. Stroke Diagnosis and Prediction Tool Using ChatGLM: Development and Validation Study. J Med Internet Res. 2025 Feb 26;27:e67010. PMID: 40009850. doi: 10.2196/67010.

21. Naeem A, Khan O, Baqir SM, Jana K, Shankar P, Kaur A, et al. Language Artificial Intelligence Models as Pioneers in Diagnostic Medicine? A Retrospective Analysis on Real-Time Patients. J Clin Med. 2025 Feb 10;14(4). PMID: 40004661. doi: 10.3390/jcm14041131.

22. Larson DB, Koirala A, Cheuy LY, Paschali M, Van Veen D, Na HS, et al. Assessing Completeness of Clinical Histories Accompanying Imaging Orders Using Adapted Open-Source and Closed-Source Large Language Models. Radiology. 2025 Feb;314(2):e241051. PMID: 39998369. doi: 10.1148/radiol.241051.

23. Turan EI, Baydemir AE, Balitatli AB, Sahin AS. Assessing the accuracy of ChatGPT in interpreting blood gas analysis results ChatGPT-4 in blood gas analysis. Journal of clinical anesthesia. 2025 Mar;102:111787. PMID: 39986120. doi: 10.1016/j.jclinane.2025.111787.

24. Hadjiathanasiou A, Goelz L, Muhn F, Heinz R, Kreissl L, Sparenberg P, et al. Artificial intelligence in neurovascular decision-making: a comparative analysis of ChatGPT-4 and multidisciplinary expert recommendations for unruptured intracranial aneurysms. Neurosurgical review. 2025 Feb 21;48(1):261. PMID: 39982556. doi: 10.1007/s10143-025-03341-3.

25. Zeljkovic I, Novak A, Lisicic A, Jordan A, Serman A, Jurin I, et al. Beyond Text: The Impact of Clinical Context on GPT-4's 12-Lead Electrocardiogram Interpretation Accuracy. The Canadian journal of cardiology. 2025 Jul;41(7):1406-14. PMID: 39971004. doi: 10.1016/j.cjca.2025.01.036.

26. Vong T, Rizer N, Jain V, Thompson VL, Dredze M, Klein EY, et al. Automated identification of incidental hepatic steatosis on Emergency Department imaging using large language models. Hepatology communications. 2025 Mar 1;9(3). PMID: 39969431. doi: 10.1097/HC9.0000000000000638.

27. Yang L, Zhou Y, Qi J, Zhen X, Sun L, Shi S, et al. Aligning large language models with radiologists by reinforcement learning from AI feedback for chest CT reports. Eur J Radiol. 2025 Mar;184:111984. PMID: 39954322. doi: 10.1016/j.ejrad.2025.111984.

28. Kavak EE, Dilli I. Progression-Free Survival Prediction Performance of ChatGPT: Analysis With Real Life Data in Early and Locally Advanced Prostate Cancer. The Prostate. 2025 May;85(7):677-83. PMID: 39948824. doi: 10.1002/pros.24871.

29. Zheng Y, Yan Y, Chen S, Cai Y, Ren K, Liu Y, et al. Integrating retrieval-augmented generation for enhanced personalized physician recommendations in web-based medical services: model development study. Front Public Health. 2025;13:1501408. PMID: 39944072. doi: 10.3389/fpubh.2025.1501408.

30. Gumus Akgun G, Altan C, Balci AS, Alagoz N, Cakir I, Yasar T. Using ChatGPT-4 in visual field test assessment. Clinical & experimental optometry. 2025 Feb 12:1-6. PMID: 39938922. doi: 10.1080/08164622.2025.2463518.

31. Hernandez-Flores LA, Lopez-Martinez JB, Rosales-de-la-Rosa JJ, Aillaud-De-Uriarte D, Contreras-Garduno S, Cortes-Gonzalez R. Assessment of Challenging Oncologic Cases: A Comparative Analysis Between ChatGPT, Gemini, and a Multidisciplinary Tumor Board. Journal of surgical oncology. 2025 Jun;131(8):1562-70. PMID: 39936586. doi: 10.1002/jso.28121.

32. Zhang Y, Kohne JG, Webster K, Vartanian R, Wittrup E, Najarian K. AXpert: human expert facilitated privacy-preserving large language models for abdominal X-ray report labeling. JAMIA open. 2025 Feb;8(1):ooaf008. PMID: 39931456. doi: 10.1093/jamiaopen/ooaf008.

33. Ozenbas C, Engin D, Altinok T, Akcay E, Aktas U, Tabanli A. ChatGPT-4o's Performance in Brain Tumor Diagnosis and MRI Findings: A Comparative Analysis with Radiologists. Acad Radiol. 2025 Jun;32(6):3608-17. PMID: 39924377. doi: 10.1016/j.acra.2025.01.033.

34. Perogamvros L, Rochas V, Beau JB, Sterpenich V, Bayer L. The cathartic dream: Using a large language model to study a new type of functional dream in healthy and clinical populations. J Sleep Res. 2025 Feb 9:e70001. PMID: 39924340. doi: 10.1111/jsr.70001.

35. Giannuzzi F, Carla MM, Hu L, Cestrone V, Caputo CG, Sammarco MG, et al. Artificial intelligence with ChatGPT 4: a large language model in support of ocular oncology cases. Int Ophthalmol. 2025 Feb 7;45(1):59. PMID: 39918656. doi: 10.1007/s10792-024-03399-w.

36. Bhayana R, Alwahbi O, Ladak AM, Deng Y, Basso Dias A, Elbanna K, et al. Leveraging Large Language Models to Generate Clinical Histories for Oncologic Imaging Requisitions. Radiology. 2025 Feb;314(2):e242134. PMID: 39903072. doi: 10.1148/radiol.242134.

37. Patel PV, Davis C, Ralbovsky A, Tinoco D, Williams CYK, Slatter S, et al. Large Language Models Outperform Traditional Natural Language Processing Methods in Extracting Patient-Reported Outcomes in Inflammatory Bowel Disease. Gastro hep advances. 2025;4(2):100563. PMID: 39877865. doi: 10.1016/j.gastha.2024.10.003.

38. Saraiva MM, Ribeiro T, Agudo B, Afonso J, Mendes F, Martins M, et al. Evaluating ChatGPT-4 for the Interpretation of Images from Several Diagnostic Techniques in Gastroenterology. J Clin Med. 2025 Jan 17;14(2). PMID: 39860582. doi: 10.3390/jcm14020572.

39. Koyun M, Taskent I. Evaluation of Advanced Artificial Intelligence Algorithms' Diagnostic Efficacy in Acute Ischemic Stroke: A Comparative Analysis of ChatGPT-4o and Claude 3.5 Sonnet Models. J Clin Med. 2025 Jan 17;14(2). PMID: 39860577. doi: 10.3390/jcm14020571.

40. Brigo F, Broggi S, Leuci E, Turcato G, Zaboli A. Can ChatGPT 4.0 Diagnose Epilepsy? A Study on Artificial Intelligence's Diagnostic Capabilities. J Clin Med. 2025 Jan 7;14(2). PMID: 39860325. doi: 10.3390/jcm14020322.

41. Koyun M, Cevval ZK, Reis B, Ece B. Detection of Intracranial Hemorrhage from Computed Tomography Images: Diagnostic Role and Efficacy of ChatGPT-4o. Diagnostics (Basel, Switzerland). 2025 Jan 9;15(2). PMID: 39857027. doi: 10.3390/diagnostics15020143.

42. Roy JM, Self DM, Isch E, Musmar B, Lan M, Keppetipola K, et al. Evaluating Large Language Models for Automated CPT Code Prediction in Endovascular Neurosurgery. Journal of medical systems. 2025 Jan 24;49(1):15. PMID: 39853605. doi: 10.1007/s10916-025-02149-4.

43. Liao C, Chu C, Lien M, Wu Y, Wang T. AI-Enhanced Healthcare: Integrating ChatGPT-4 in ePROs for Improved Oncology Care and Decision-Making: A Pilot Evaluation. Current oncology (Toronto, Ont). 2024 Dec 26;32(1). PMID: 39851923. doi: 10.3390/curroncol32010007.

44. Malik J, Afzal MW, Khan SS, Umer MR, Fakhar B, Mehmoodi A. Role of Artificial Intelligence-assisted Decision Support Tool for Common Rhythm Disturbances: A ChatGPT Proof-of-concept Study. Journal of community hospital internal medicine perspectives. 2024;14(6):5-9. PMID: 39839170. doi: 10.55729/2000-9666.1402.

45. Yelin D, Shirin N, Harris I, Peretz Y, Yahav D, Schwartz E, et al. Performance of ChatGPT-4o in the diagnostic workup of fever among returning travellers requiring hospitalization: a validation study. J Travel Med. 2025 Apr 25;32(4). PMID: 39823287. doi: 10.1093/jtm/taaf005.

46. Hu X, Xu D, Zhang H, Tang M, Gao Q. Comparative diagnostic accuracy of ChatGPT-4 and machine learning in differentiating spinal tuberculosis and spinal tumors. The spine journal : official journal of the North American Spine Society. 2025 Jun;25(6):1196-205. PMID: 39805470. doi: 10.1016/j.spinee.2024.12.035.

47. Burgisser N, Chalot E, Mehouachi S, Buclin CP, Lauper K, Courvoisier DS, et al. Large language models for accurate disease detection in electronic health records: the examples of crystal arthropathies. RMD open. 2024 Dec 20;10(4). PMID: 39794274. doi: 10.1136/rmdopen-2024-005003.

48. Vueghs C, Shakeri H, Renton T, Van der Cruyssen F. Development and Evaluation of a GPT4-Based Orofacial Pain Clinical Decision Support System. Diagnostics (Basel, Switzerland). 2024 Dec 17;14(24). PMID: 39767196. doi: 10.3390/diagnostics14242835.

49. Huang J, Yang R, Huang X, Zeng K, Liu Y, Luo J, et al. Feasibility of large language models for CEUS LI-RADS categorization of small liver nodules in patients at risk for hepatocellular carcinoma. Front Oncol. 2024;14:1513608. PMID: 39744002. doi: 10.3389/fonc.2024.1513608.

50. Arslan B, Nuhoglu C, Satici MO, Altinbilek E. Evaluating LLM-based generative AI tools in emergency triage: A comparative study of ChatGPT Plus, Copilot Pro, and triage nurses. The American journal of emergency medicine. 2025 Mar;89:174-81. PMID: 39731895. doi: 10.1016/j.ajem.2024.12.024.

51. Menezes MCS, Hoffmann AF, Tan ALM, Nalbandyan M, Omenn GS, Mazzotti DR, et al. The potential of Generative Pre-trained Transformer 4 (GPT-4) to analyse medical notes in three different languages: a retrospective model-evaluation study. The Lancet Digital health. 2025 Jan;7(1):e35-e43. PMID: 39722251. doi: 10.1016/S2589-7500(24)00246-2.

52. Carla MM, Crincoli E, Rizzo S. RETINAL IMAGING ANALYSIS PERFORMED BY CHATGPT-4o AND GEMINI ADVANCED: The Turning Point of the Revolution? Retina. 2025 Apr 1;45(4):694-702. PMID: 39715322. doi: 10.1097/IAE.0000000000004351.

53. Cuevas-Nunez M, Silberberg VIA, Arregui M, Jham BC, Ballester-Victoria R, Koptseva I, et al. Diagnostic performance of ChatGPT-4.0 in histopathological description analysis of oral and maxillofacial lesions: a comparative study with pathologists. Oral surgery, oral medicine, oral pathology and oral radiology. 2025 Apr;139(4):453-61. PMID: 39709300. doi: 10.1016/j.oooo.2024.11.087.

54. Boussina A, Krishnamoorthy R, Quintero K, Joshi S, Wardi G, Pour H, et al. Large Language Models for More Efficient Reporting of Hospital Quality Measures. Nejm ai. 2024 Oct 24;1(11). PMID: 39703686. doi: 10.1056/aics2400420.

55. Bannett Y, Gunturkun F, Pillai M, Herrmann JE, Luo I, Huffman LC, et al. Applying Large Language Models to Assess Quality of Care: Monitoring ADHD Medication Side Effects. Pediatrics. 2025 Jan 1;155(1). PMID: 39701141. doi: 10.1542/peds.2024-067223.

56. Flanagan CP, Trang K, Nacario J, Schneider PA, Gasper WJ, Conte MS, et al. Large language models can accurately populate Vascular Quality Initiative procedural databases using narrative operative reports. J Vasc Surg. 2025 Apr;81(4):973-82. PMID: 39694151. doi: 10.1016/j.jvs.2024.12.002.

57. Wang Y, Zhang J, Li M, Miao Z, Wang J, He K, et al. SMART: Development and Application of a Multimodal Multi-organ Trauma Screening Model for Abdominal Injuries in Emergency Settings. Acad Radiol. 2025 May;32(5):2655-66. PMID: 39690074. doi: 10.1016/j.acra.2024.11.056.

58. Feng R, Brennan KA, Azizi Z, Goyal J, Deb B, Chang HJ, et al. Engineering of Generative Artificial Intelligence and Natural Language Processing Models to Accurately Identify Arrhythmia Recurrence. Circulation Arrhythmia and electrophysiology. 2025 Jan;18(1):e013023. PMID: 39676642. doi: 10.1161/CIRCEP.124.013023.

59. Chung D, Sidhom K, Dhillon H, Bal DS, Fidel MG, Jawanda G, et al. Real-world utility of ChatGPT in pre-vasectomy counselling, a safe and efficient practice: a prospective single-centre clinical study. World journal of urology. 2024 Dec 14;43(1):32. PMID: 39673635. doi: 10.1007/s00345-024-05385-4.

60. Yasaka K, Nomura T, Kamohara J, Hirakawa H, Kubo T, Kiryu S, et al. Classification of Interventional Radiology Reports into Technique Categories with a Fine-Tuned Large Language Model. Journal of imaging informatics in medicine. 2024 Dec 13. PMID: 39673010. doi: 10.1007/s10278-024-01370-w.

61. Hoffman H, Sequeiros Chirinos J, Khan N, Nickele C, Inoa V, Elijovich L, et al. Prediction of Symptomatic Intracranial Hemorrhage Before Mechanical Thrombectomy Using Machine Learning in Patients with Anterior Circulation Large Vessel Occlusion. World neurosurgery. 2025 Feb;194:123455. PMID: 39577637. doi: 10.1016/j.wneu.2024.11.038.

62. Zaboli A, Brigo F, Ziller M, Massar M, Parodi M, Magnarelli G, et al. Exploring ChatGPT's potential in ECG interpretation and outcome prediction in emergency department. The American journal of emergency medicine. 2025 Feb;88:7-11. PMID: 39566376. doi: 10.1016/j.ajem.2024.11.023.

63. Dana Z, Nagra H, Kilby K. Role of Synchronous, Moderated, and Anonymous Peer Support Chats on Reducing Momentary Loneliness in Older Adults: Retrospective Observational Study. JMIR formative research. 2024 Oct 25;8:e59501. PMID: 39453688. doi: 10.2196/59501.

64. Tortum F, Kasali K. Exploring the potential of artificial intelligence models for triage in the emergency department. Postgraduate medicine. 2024 Nov;136(8):841-6. PMID: 39420246. doi: 10.1080/00325481.2024.2418806.

65. Far AT, Bastani A, Lee A, Gologorskaya O, Huang CY, Pletcher MJ, et al. Evaluating the positive predictive value of code-based identification of cirrhosis and its complications utilizing GPT-4. Hepatology (Baltimore, Md). 2025 Jun 1;81(6):1753-63. PMID: 39378414. doi: 10.1097/HEP.0000000000001115.

66. Alanzi TM, Alharthi A, Alrumman S, Abanmi S, Jumah A, Alansari H, et al. ChatGPT as a psychotherapist for anxiety disorders: An empirical study with anxiety patients. Nutr Health. 2024 Oct 7:2601060241281906. PMID: 39370914. doi: 10.1177/02601060241281906.

67. Mert M, Vahabi A, Dastan AE, Kuyucu A, Unal YC, Tezgel O, et al. Artificial intelligence's suggestions for level of amputation in diabetic foot ulcers are highly correlated with those of clinicians, only with exception of hindfoot amputations. Int Wound J. 2024 Oct;21(10):e70055. PMID: 39353602. doi: 10.1111/iwj.70055.

68. Lan L, Yang L, Li J, Hou J, Yan Y, Zhang Y. Establishing a novel score system and using it to assess and compare the quality of ChatGPT-4 consultation with physician consultation for obstetrics and gynecology: A pilot study. International journal of gynaecology and obstetrics: the official organ of the International Federation of Gynaecology and Obstetrics. 2025 Mar;168(3):1251-7. PMID: 39340470. doi: 10.1002/ijgo.15934.

69. Zhang D, Ma Z, Gong R, Lian L, Li Y, He Z, et al. Using Natural Language Processing (GPT-4) for Computed Tomography Image Analysis of Cerebral Hemorrhages in Radiology: Retrospective Analysis. J Med Internet Res. 2024 Sep 26;26:e58741. PMID: 39326037. doi: 10.2196/58741.

70. Maniaci A, Chiesa-Estomba CM, Lechien JR. ChatGPT-4 Consistency in Interpreting Laryngeal Clinical Images of Common Lesions and Disorders. Otolaryngology--head and neck surgery : official journal of American Academy of Otolaryngology-Head and Neck Surgery. 2024 Oct;171(4):1106-13. PMID: 39045737. doi: 10.1002/ohn.897.

71. Laohawetwanit T, Apornvirat S, Namboonlue C. Thinking like a pathologist: Morphologic approach to hepatobiliary tumors by ChatGPT. Am J Clin Pathol. 2025 Jan 28;163(1):3-11. PMID: 39030695. doi: 10.1093/ajcp/aqae087.

72. Hoppe JM, Auer MK, Struven A, Massberg S, Stremmel C. ChatGPT With GPT-4 Outperforms Emergency Department Physicians in Diagnostic Accuracy: Retrospective Analysis. J Med Internet Res. 2024 Jul 8;26:e56110. PMID: 38976865. doi: 10.2196/56110.

73. Shapiro J, Baum S, Pavlotzky F, Mordehai YB, Barzilai A, Freud T, et al. Application of a natural language processing artificial intelligence tool in psoriasis: A cross-sectional comparative study on identifying affected areas in patients' data. Clinics in dermatology. 2024 Sep-Oct;42(5):480-6. PMID: 38909857. doi: 10.1016/j.clindermatol.2024.06.018.

74. Schmidl B, Hutten T, Pigorsch S, Stogbauer F, Hoch CC, Hussain T, et al. Assessing the role of advanced artificial intelligence as a tool in multidisciplinary tumor board decision-making for primary head and neck cancer cases. Front Oncol. 2024;14:1353031. PMID: 38854718. doi: 10.3389/fonc.2024.1353031.

75. Lopez-Gonzalez R, Sanchez-Cordero S, Pujol-Gebelli J, Castellvi J. Evaluation of the Impact of ChatGPT on the Selection of Surgical Technique in Bariatric Surgery. Obesity surgery. 2025 Jan;35(1):19-24. PMID: 38760650. doi: 10.1007/s11695-024-07279-1.

76. Cuellar-Barboza A, Brussolo-Marroquin E, Cordero-Martinez FC, Aguilar-Calderon PE, Vazquez-Martinez O, Ocampo-Candiani J. An evaluation of ChatGPT compared with dermatological surgeons' choices of reconstruction for surgical defects after Mohs surgery. Clinical and experimental dermatology. 2024 Oct 24;49(11):1367-71. PMID: 38738492. doi: 10.1093/ced/llae184.

77. Moulson R, Law J, Sacher A, Liu G, Shepherd FA, Bradbury P, et al. Real-World Outcomes of Patients with Advanced Epidermal Growth Factor Receptor-Mutated Non-Small Cell Lung Cancer in Canada Using Data Extracted by Large Language Model-Based Artificial Intelligence. Current oncology (Toronto, Ont). 2024 Apr 2;31(4):1947-60. PMID: 38668049. doi: 10.3390/curroncol31040146.

78. Turan EI, Baydemir AE, Ozcan FG, Sahin AS. Evaluating the accuracy of ChatGPT-4 in predicting ASA scores: A prospective multicentric study ChatGPT-4 in ASA score prediction. Journal of clinical anesthesia. 2024 Sep;96:111475. PMID: 38657530. doi: 10.1016/j.jclinane.2024.111475.

79. Lechien JR, Naunheim MR, Maniaci A, Radulesco T, Saibene AM, Chiesa-Estomba CM, et al. Performance and Consistency of ChatGPT-4 Versus Otolaryngologists: A Clinical Case Series. Otolaryngology--head and neck surgery : official journal of American Academy of Otolaryngology-Head and Neck Surgery. 2024 Jun;170(6):1519-26. PMID: 38591726. doi: 10.1002/ohn.759.

80. Yahagi M, Hiruta R, Miyauchi C, Tanaka S, Taguchi A, Yaguchi Y. Comparison of Conventional Anesthesia Nurse Education and an Artificial Intelligence Chatbot (ChatGPT) Intervention on Preoperative Anxiety: A Randomized Controlled Trial. Journal of perianesthesia nursing : official journal of the American Society of PeriAnesthesia Nurses. 2024 Oct;39(5):767-71. PMID: 38520470. doi: 10.1016/j.jopan.2023.12.005.

81. Haim GB, Braun A, Eden H, Burshtein L, Barash Y, Irony A, et al. AI in the ED: Assessing the efficacy of GPT models vs. physicians in medical score calculation. The American journal of emergency medicine. 2024 May;79:161-6. PMID: 38447503. doi: 10.1016/j.ajem.2024.02.016.

82. Gakuba C, Le Barbey C, Sar A, Bonnet G, Cerasuolo D, Giabicani M, et al. Evaluation of ChatGPT in Predicting 6-Month Outcomes After Traumatic Brain Injury. Crit Care Med. 2024 Jun 1;52(6):942-50. PMID: 38445975. doi: 10.1097/CCM.0000000000006236.

83. Sievert M, Conrad O, Mueller SK, Rupp R, Balk M, Richter D, et al. Risk stratification of thyroid nodules: Assessing the suitability of ChatGPT for text-based analysis. Am J Otolaryngol. 2024 Mar-Apr;45(2):104144. PMID: 38113774. doi: 10.1016/j.amjoto.2023.104144.

84. Lechien JR, Chiesa-Estomba CM, Baudouin R, Hans S. Accuracy of ChatGPT in head and neck oncological board decisions: preliminary findings. Eur Arch Otorhinolaryngol. 2024 Apr;281(4):2105-14. PMID: 37991498. doi: 10.1007/s00405-023-08326-w.

85. Stoneham S, Livesey A, Cooper H, Mitchell C. ChatGPT versus clinician: challenging the diagnostic capabilities of artificial intelligence in dermatology. Clinical and experimental dermatology. 2024 Jun 25;49(7):707-10. PMID: 37979201. doi: 10.1093/ced/llad402.

86. Buzancic I, Belec D, Drzaic M, Kummer I, Brkic J, Fialova D, et al. Clinical decision-making in benzodiazepine deprescribing by healthcare providers vs. AI-assisted approach. Br J Clin Pharmacol. 2024 Mar;90(3):662-74. PMID: 37949663. doi: 10.1111/bcp.15963.

87. Choo JM, Ryu HS, Kim JS, Cheong JY, Baek SJ, Kwak JM, et al. Conversational artificial intelligence (chatGPT) in the management of complex colorectal cancer patients: early experience. ANZ journal of surgery. 2024 Mar;94(3):356-61. PMID: 37905713. doi: 10.1111/ans.18749.

88. Maillard A, Micheli G, Lefevre L, Guyonnet C, Poyart C, Canoui E, et al. Can Chatbot Artificial Intelligence Replace Infectious Diseases Physicians in the Management of Bloodstream Infections? A Prospective Cohort Study. Clinical infectious diseases : an official publication of the Infectious Diseases Society of America. 2024 Apr 10;78(4):825-32. PMID: 37823416. doi: 10.1093/cid/ciad632.

89. Cocci A, Pezzoli M, Lo Re M, Russo GI, Asmundo MG, Fode M, et al. Quality of information and appropriateness of ChatGPT outputs for urology patients. Prostate cancer and prostatic diseases. 2024 Mar;27(1):103-8. PMID: 37516804. doi: 10.1038/s41391-023-00705-y.

90. Gebrael G, Sahu KK, Chigarira B, Tripathi N, Mathew Thomas V, Sayegh N, et al. Enhancing Triage Efficiency and Accuracy in Emergency Rooms for Patients with Metastatic Prostate Cancer: A Retrospective Analysis of Artificial Intelligence-Assisted Triage Using ChatGPT 4.0. Cancers. 2023 Jul 22;15(14). PMID: 37509379. doi: 10.3390/cancers15143717.

91. Haemmerli J, Sveikata L, Nouri A, May A, Egervari K, Freyschlag C, et al. ChatGPT in glioma adjuvant therapy decision making: ready to assume the role of a doctor in the tumour board? BMJ health & care informatics. 2023 Jun;30(1). PMID: 37399360. doi: 10.1136/bmjhci-2023-100775.

92. Bailey DJ, Herget F, Hansen D, Burton F, Pitt G, Harmon T, et al. Generative AI applied to AAC for aphasia: a pilot study of Aphasia-GPT. Aphasiology. 2024:1-16. doi: 10.1080/02687038.2024.2445663.

93. Barzkar F, Zaribaf A, Mirfazeli FS, Keshavarz-Akhlaghi A-A. The Machine as Therapist: Unpacking Transference and Emotional Healing in AI-Assisted Therapy. Journal of Contemporary Psychotherapy. 2025. doi: 10.1007/s10879-025-09677-7.

94. Huang L, Lin A, Li H, Wang Q, Shen J, Jiang A, et al. Unveiling large multimodal models in pulmonary CT: A comparative assessment of generative AI performance in lung cancer diagnostics. View. 2025. doi: 10.1002/viw.20250077.

95. Wang Z, Guo R, Sun P, Qian L, Hu X. Enhancing Diagnostic Accuracy and Efficiency with GPT-4-Generated Structured Reports: A Comprehensive Study. Journal of Medical and Biological Engineering. 2024;44(1):144-53. doi: 10.1007/s40846-024-00849-9.

96. Song M, Wang J, Yu Z, Wang J, Yang L, Lu Y, et al. PneumoLLM: Harnessing the power of large language model for pneumoconiosis diagnosis. Med Image Anal. 2024 Oct;97:103248. PMID: 38941859. doi: 10.1016/j.media.2024.103248.

97. Alsentzer E, Rasmussen MJ, Fontoura R, Cull AL, Beaulieu-Jones B, Gray KJ, et al. Zero-shot interpretable phenotyping of postpartum hemorrhage using large language models. NPJ Digit Med. 2023 Nov 30;6(1):212. PMID: 38036723. doi: 10.1038/s41746-023-00957-x.

98. Williams CYK, Zack T, Miao BY, Sushil M, Wang M, Kornblith AE, et al. Use of a Large Language Model to Assess Clinical Acuity of Adults in the Emergency Department. JAMA Netw Open. 2024 May 1;7(5):e248895. PMID: 38713466. doi: 10.1001/jamanetworkopen.2024.8895.

99. Venerito V, Iannone F. Large language model-driven sentiment analysis for facilitating fibromyalgia diagnosis. RMD open. 2024 Jun 28;10(2). PMID: 38942593. doi: 10.1136/rmdopen-2024-004367.

100. Yuan J, Tang R, Jiang X, Hu X. Large Language Models for Healthcare Data Augmentation: An Example on Patient-Trial Matching. AMIA Annual Symposium proceedings AMIA Symposium. 2023;2023:1324-33. PMID: 38222339.

101. Xie K, Ojemann WKS, Gallagher RS, Shinohara RT, Lucas A, Hill CE, et al. Disparities in seizure outcomes revealed by large language models. Journal of the American Medical Informatics Association : JAMIA. 2024 May 20;31(6):1348-55. PMID: 38481027. doi: 10.1093/jamia/ocae047.

102. Zhu Z, Zhao Q, Li J, Ge Y, Ding X, Gu T, et al. Comparative Analysis of Large Language Models in Chinese Medical Named Entity Recognition. Bioengineering (Basel, Switzerland). 2024 Sep 29;11(10). PMID: 39451358. doi: 10.3390/bioengineering11100982.

103. Frosolini A, Catarzi L, Benedetti S, Latini L, Chisci G, Franz L, et al. The Role of Large Language Models (LLMs) in Providing Triage for Maxillofacial Trauma Cases: A Preliminary Study. Diagnostics (Basel, Switzerland). 2024 Apr 18;14(8). PMID: 38667484. doi: 10.3390/diagnostics14080839.

104. Tang CC, Nagesh S, Fussell DA, Glavis-Bloom J, Mishra N, Li C, et al. Generating colloquial radiology reports with large language models. Journal of the American Medical Informatics Association : JAMIA. 2024 Nov 1;31(11):2660-7. PMID: 39178375. doi: 10.1093/jamia/ocae223.

105. Williams CYK, Miao BY, Kornblith AE, Butte AJ. Evaluating the use of large language models to provide clinical recommendations in the Emergency Department. Nat Commun. 2024 Oct 8;15(1):8236. PMID: 39379357. doi: 10.1038/s41467-024-52415-1.

106. Tie X, Shin M, Pirasteh A, Ibrahim N, Huemann Z, Castellino SM, et al. Personalized Impression Generation for PET Reports Using Large Language Models. Journal of imaging informatics in medicine. 2024 Apr;37(2):471-88. PMID: 38308070. doi: 10.1007/s10278-024-00985-3.

107. Klang E, Apakama D, Abbott EE, Vaid A, Lampert J, Sakhuja A, et al. A strategy for cost-effective large language model use at health system-scale. NPJ Digit Med. 2024 Nov 18;7(1):320. PMID: 39558090. doi: 10.1038/s41746-024-01315-1.

108. Liu S, Wright AP, McCoy AB, Huang SS, Genkins JZ, Peterson JF, et al. Using large language model to guide patients to create efficient and comprehensive clinical care message. Journal of the American Medical Informatics Association : JAMIA. 2024 Aug 1;31(8):1665-70. PMID: 38917441. doi: 10.1093/jamia/ocae142.

109. Sun D, Hadjiiski L, Gormley J, Chan HP, Caoili E, Cohan R, et al. Outcome Prediction Using Multi-Modal Information: Integrating Large Language Model-Extracted Clinical Information and Image Analysis. Cancers. 2024 Jun 29;16(13). PMID: 39001463. doi: 10.3390/cancers16132402.

110. Sushil M, Zack T, Mandair D, Zheng Z, Wali A, Yu YN, et al. A comparative study of large language model-based zero-shot inference and task-specific supervised classification of breast cancer pathology reports. Journal of the American Medical Informatics Association : JAMIA. 2024 Oct 1;31(10):2315-27. PMID: 38900207. doi: 10.1093/jamia/ocae146.

111. Woznicki P, Laqua C, Fiku I, Hekalo A, Truhn D, Engelhardt S, et al. Automatic structuring of radiology reports with on-premise open-source large language models. European radiology. 2025 Apr;35(4):2018-29. PMID: 39390261. doi: 10.1007/s00330-024-11074-y.

112. Liu J, Koopman B, Brown NJ, Chu K, Nguyen A. Generating synthetic clinical text with local large language models to identify misdiagnosed limb fractures in radiology reports. Artif Intell Med. 2025 Jan;159:103027. PMID: 39580897. doi: 10.1016/j.artmed.2024.103027.

113. Haim GB, Saban M, Barash Y, Cirulnik D, Shaham A, Eisenman BZ, et al. Evaluating Large Language Model-Assisted Emergency Triage: A Comparison of Acuity Assessments by GPT-4 and Medical Experts. Journal of clinical nursing. 2024 Nov 28. PMID: 39610042. doi: 10.1111/jocn.17490.

114. Lopez-Ubeda P, Martin-Noguerol T, Diaz-Angulo C, Luna A. Evaluation of large language models performance against humans for summarizing MRI knee radiology reports: A feasibility study. Int J Med Inform. 2024 Jul;187:105443. PMID: 38615509. doi: 10.1016/j.ijmedinf.2024.105443.

115. Luo M, Trivedi S, Kurian AW, Ward K, Keegan THM, Rubin D, et al. Automated Extraction of Patient-Centered Outcomes After Breast Cancer Treatment: An Open-Source Large Language Model-Based Toolkit. JCO clinical cancer informatics. 2024 Aug;8:e2300258. PMID: 39167746. doi: 10.1200/CCI.23.00258.

116. Cheligeer K, Wu G, Laws A, Quan ML, Li A, Brisson AM, et al. Validation of large language models for detecting pathologic complete response in breast cancer using population-based pathology reports. BMC Med Inform Decis Mak. 2024 Oct 3;24(1):283. PMID: 39363322. doi: 10.1186/s12911-024-02677-y.

117. Urquhart E, Ryan J, Hartigan S, Nita C, Hanley C, Moran P, et al. A pilot feasibility study comparing large language models in extracting key information from ICU patient text records from an Irish population. Intensive care medicine experimental. 2024 Aug 16;12(1):71. PMID: 39147878. doi: 10.1186/s40635-024-00656-1.

118. Ralevski A, Taiyab N, Nossal M, Mico L, Piekos S, Hadlock J. Using Large Language Models to Abstract Complex Social Determinants of Health From Original and Deidentified Medical Notes: Development and Validation Study. J Med Internet Res. 2024 Nov 19;26:e63445. PMID: 39561354. doi: 10.2196/63445.

119. Holmes J, Zhang L, Ding Y, Feng H, Liu Z, Liu T, et al. Benchmarking a Foundation Large Language Model on its Ability to Relabel Structure Names in Accordance With the American Association of Physicists in Medicine Task Group-263 Report. Practical radiation oncology. 2024 Nov-Dec;14(6):e515-e21. PMID: 39243241. doi: 10.1016/j.prro.2024.04.017.

120. Eghbali N, Klochko C, Razoky P, Chintalapati P, Jawad E, Mahdi Z, et al. Improving Automating Quality Control in Radiology: Leveraging Large Language Models to Extract Correlative Findings in Radiology and Operative Reports. AMIA Joint Summits on Translational Science proceedings AMIA Joint Summits on Translational Science. 2024;2024:135-44. PMID: 38827099.

121. Patra BG, Lepow LA, Kasi Reddy Jagadeesh Kumar P, Vekaria V, Sharma MM, Adekkanattu P, et al. Extracting social support and social isolation information from clinical psychiatry notes: comparing a rule-based natural language processing system and a large language model. Journal of the American Medical Informatics Association : JAMIA. 2025 Jan 1;32(1):218-26. PMID: 39423850. doi: 10.1093/jamia/ocae260.

122. Fong A, Adams KT, Boxley C, Revoir JA, Krevat S, Ratwani RM. Does one size fit all? Developing an evaluation strategy to assess large language models for patient safety event report analysis. JAMIA open. 2024 Dec;7(4):ooae128. PMID: 39524608. doi: 10.1093/jamiaopen/ooae128.

123. Serapio A, Chaudhari G, Savage C, Lee YJ, Vella M, Sridhar S, et al. An open-source fine-tuned large language model for radiological impression generation: a multi-reader performance study. BMC Med Imaging. 2024 Sep 27;24(1):254. PMID: 39333958. doi: 10.1186/s12880-024-01435-w.

124. Butler JJ, Puleo J, Harrington MC, Dahmen J, Rosenbaum AJ, Kerkhoffs G, et al. From technical to understandable: Artificial Intelligence Large Language Models improve the readability of knee radiology reports. Knee surgery, sports traumatology, arthroscopy : official journal of the ESSKA. 2024 May;32(5):1077-86. PMID: 38488217. doi: 10.1002/ksa.12133.

125. Jaskari J, Sahlsten J, Summanen P, Moilanen J, Lehtola E, Aho M, et al. DR-GPT: A large language model for medical report analysis of diabetic retinopathy patients. PLoS One. 2024;19(10):e0297706. PMID: 39392790. doi: 10.1371/journal.pone.0297706.

126. Liu Y, Melton GB, Zhang R. Exploring Large Language Models for Acronym, Symbol Sense Disambiguation, and Semantic Similarity and Relatedness Assessment. AMIA Joint Summits on Translational Science proceedings AMIA Joint Summits on Translational Science. 2024;2024:324-33. PMID: 38827102.

127. Wang L, Ma Y, Bi W, Lv H, Li Y. An Entity Extraction Pipeline for Medical Text Records Using Large Language Models: Analytical Study. J Med Internet Res. 2024 Mar 29;26:e54580. PMID: 38551633. doi: 10.2196/54580.

128. Oh Y, Park S, Byun HK, Cho Y, Lee IJ, Kim JS, et al. LLM-driven multimodal target volume contouring in radiation oncology. Nat Commun. 2024 Oct 24;15(1):9186. PMID: 39448587. doi: 10.1038/s41467-024-53387-y.

129. Wu SH, Tong WJ, Li MD, Hu HT, Lu XZ, Huang ZR, et al. Collaborative Enhancement of Consistency and Accuracy in US Diagnosis of Thyroid Nodules Using Large Language Models. Radiology. 2024 Mar;310(3):e232255. PMID: 38470237. doi: 10.1148/radiol.232255.

130. Xia S, Hua Q, Mei Z, Xu W, Lai L, Wei M, et al. Clinical application potential of large language model: a study based on thyroid nodules. Endocrine. 2025 Jan;87(1):206-13. PMID: 39080210. doi: 10.1007/s12020-024-03981-3.

131. Truhn D, Weber CD, Braun BJ, Bressem K, Kather JN, Kuhl C, et al. A pilot study on the efficacy of GPT-4 in providing orthopedic treatment recommendations from MRI reports. Sci Rep. 2023 Nov 17;13(1):20159. PMID: 37978240. doi: 10.1038/s41598-023-47500-2.

132. Zheng NS, Keloth VK, You K, Kats D, Li DK, Deshpande O, et al. Detection of Gastrointestinal Bleeding With Large Language Models to Aid Quality Improvement and Appropriate Reimbursement. Gastroenterology. 2025 Jan;168(1):111-20 e4. PMID: 39304088. doi: 10.1053/j.gastro.2024.09.014.

133. McCoy TH, Jr., Perlis RH. Dimensional Measures of Psychopathology in Children and Adolescents Using Large Language Models. Biol Psychiatry. 2024 Dec 15;96(12):940-7. PMID: 38866172. doi: 10.1016/j.biopsych.2024.05.008.

134. Park HJ, Huh JY, Chae G, Choi MG. Extraction of clinical data on major pulmonary diseases from unstructured radiologic reports using a large language model. PLoS One. 2024;19(11):e0314136. PMID: 39585830. doi: 10.1371/journal.pone.0314136.

135. Garcia-Barragan A, Sakor A, Vidal ME, Menasalvas E, Gonzalez JCS, Provencio M, et al. NSSC: a neuro-symbolic AI system for enhancing accuracy of named entity recognition and linking from oncologic clinical notes. Medical & biological engineering & computing. 2025 Mar;63(3):749-72. PMID: 39485651. doi: 10.1007/s11517-024-03227-4.

136. Goh R, Cook B, Stretton B, Booth AE, Satheakeerthy S, Howson S, et al. Large language models can effectively extract stroke and reperfusion audit data from medical free-text discharge summaries. Journal of clinical neuroscience : official journal of the Neurosurgical Society of Australasia. 2024 Nov;129:110847. PMID: 39305548. doi: 10.1016/j.jocn.2024.110847.

137. Chen K, Xu W, Li X. The Potential of Gemini and GPTs for Structured Report Generation based on Free-Text (18)F-FDG PET/CT Breast Cancer Reports. Acad Radiol. 2025 Feb;32(2):624-33. PMID: 39245597. doi: 10.1016/j.acra.2024.08.052.

138. Gu Z, He X, Yu P, Jia W, Yang X, Peng G, et al. Automatic quantitative stroke severity assessment based on Chinese clinical named entity recognition with domain-adaptive pre-trained large language model. Artif Intell Med. 2024 Apr;150:102822. PMID: 38553162. doi: 10.1016/j.artmed.2024.102822.

139. Seth I, Lim B, Phan R, Xie Y, Kenney PS, Bukret WE, et al. Perforator Selection with Computed Tomography Angiography for Unilateral Breast Reconstruction: A Clinical Multicentre Analysis. Medicina (Kaunas, Lithuania). 2024 Sep 14;60(9). PMID: 39336540. doi: 10.3390/medicina60091500.

140. Maroncelli R, Rizzo V, Pasculli M, Cicciarelli F, Macera M, Galati F, et al. Probing clarity: AI-generated simplified breast imaging reports for enhanced patient comprehension powered by ChatGPT-4o. European radiology experimental. 2024 Oct 30;8(1):124. PMID: 39477904. doi: 10.1186/s41747-024-00526-1.

141. Gupta S, Basu A, Nievas M, Thomas J, Wolfrath N, Ramamurthi A, et al. PRISM: Patient Records Interpretation for Semantic clinical trial Matching system using large language models. NPJ Digit Med. 2024 Oct 28;7(1):305. PMID: 39468259. doi: 10.1038/s41746-024-01274-7.

142. Wang L, Bi W, Zhao S, Ma Y, Lv L, Meng C, et al. Investigating the Impact of Prompt Engineering on the Performance of Large Language Models for Standardizing Obstetric Diagnosis Text: Comparative Study. JMIR formative research. 2024 Feb 8;8:e53216. PMID: 38329787. doi: 10.2196/53216.

143. Choi DH, Kim Y, Choi SW, Kim KH, Choi Y, Shin SD. Using Large Language Models to Extract Core Injury Information From Emergency Department Notes. Journal of Korean medical science. 2024 Dec 2;39(46):e291. PMID: 39623965. doi: 10.3346/jkms.2024.39.e291.

144. Lee KH, Lee RW, Kwon YE. Validation of a Deep Learning Chest X-ray Interpretation Model: Integrating Large-Scale AI and Large Language Models for Comparative Analysis with ChatGPT. Diagnostics (Basel, Switzerland). 2023 Dec 30;14(1). PMID: 38201398. doi: 10.3390/diagnostics14010090.

145. Acharya A, Shrestha S, Chen A, Conte J, Avramovic S, Sikdar S, et al. Clinical risk prediction using language models: benefits and considerations. Journal of the American Medical Informatics Association : JAMIA. 2024 Sep 1;31(9):1856-64. PMID: 38412328. doi: 10.1093/jamia/ocae030.

146. Sorin V, Klang E, Sobeh T, Konen E, Shrot S, Livne A, et al. Generative pre-trained transformer (GPT)-4 support for differential diagnosis in neuroradiology. Quantitative imaging in medicine and surgery. 2024 Oct 1;14(10):7551-60. PMID: 39429611. doi: 10.21037/qims-24-200.

147. Chen Z, Chambara N, Wu C, Lo X, Liu SYW, Gunda ST, et al. Assessing the feasibility of ChatGPT-4o and Claude 3-Opus in thyroid nodule classification based on ultrasound images. Endocrine. 2025 Mar;87(3):1041-9. PMID: 39394537. doi: 10.1007/s12020-024-04066-x.

148. Lee C, Mohebbi M, O'Callaghan E, Winsberg M. Large Language Models Versus Expert Clinicians in Crisis Prediction Among Telemental Health Patients: Comparative Study. JMIR mental health. 2024 Aug 2;11:e58129. PMID: 38876484. doi: 10.2196/58129.

149. Lyu Q, Tan J, Zapadka ME, Ponnatapura J, Niu C, Myers KJ, et al. Translating radiology reports into plain language using ChatGPT and GPT-4 with prompt learning: results, limitations, and potential. Visual computing for industry, biomedicine, and art. 2023 May 18;6(1):9. PMID: 37198498. doi: 10.1186/s42492-023-00136-5.

150. Yang X, Chen A, PourNejatian N, Shin HC, Smith KE, Parisien C, et al. A large language model for electronic health records. NPJ Digit Med. 2022 Dec 26;5(1):194. PMID: 36572766. doi: 10.1038/s41746-022-00742-2.

151. Choi HS, Song JY, Shin KH, Chang JH, Jang BS. Developing prompts from large language model for extracting clinical information from pathology and ultrasound reports in breast cancer. Radiation oncology journal. 2023 Sep;41(3):209-16. PMID: 37793630. doi: 10.3857/roj.2023.00633.

152. Wang M, Wei J, Zeng Y, Dai L, Yan B, Zhu Y, et al. Precision Structuring of Free-Text Surgical Record for Enhanced Stroke Management: A Comparative Evaluation of Large Language Models. Journal of multidisciplinary healthcare. 2024;17:5163-75. PMID: 39558925. doi: 10.2147/JMDH.S486449.

153. Le Guellec B, Lefevre A, Geay C, Shorten L, Bruge C, Hacein-Bey L, et al. Performance of an Open-Source Large Language Model in Extracting Information from Free-Text Radiology Reports. Radiology Artificial intelligence. 2024 Jul;6(4):e230364. PMID: 38717292. doi: 10.1148/ryai.230364.

154. Wan P, Huang Z, Tang W, Nie Y, Pei D, Deng S, et al. Outpatient reception via collaboration between nurses and a large language model: a randomized controlled trial. Nat Med. 2024 Oct;30(10):2878-85. PMID: 39009780. doi: 10.1038/s41591-024-03148-7.

155. Li R, Wang X, Yu H. Two Directions for Clinical Data Generation with Large Language Models: Data-to-Label and Label-to-Data. Proceedings of the Conference on Empirical Methods in Natural Language Processing Conference on Empirical Methods in Natural Language Processing. 2023 Dec;2023:7129-43. PMID: 38213944. doi: 10.18653/v1/2023.findings-emnlp.474.

156. Kim J, Lee S, Jeon H, Lee KJ, Bae HJ, Kim B, et al. PhenoFlow: A Human-LLM Driven Visual Analytics System for Exploring Large and Complex Stroke Datasets. IEEE Trans Vis Comput Graph. 2025 Jan;31(1):470-80. PMID: 39316495. doi: 10.1109/TVCG.2024.3456215.

157. Hallinan J, Leow NW, Ong W, Lee A, Low YX, Chan MDZ, et al. MRI spine request form enhancement and auto protocoling using a secure institutional large language model. The spine journal : official journal of the North American Spine Society. 2025 Mar;25(3):505-14. PMID: 39536908. doi: 10.1016/j.spinee.2024.10.021.

158. Hartman V, Zhang X, Poddar R, McCarty M, Fortenko A, Sholle E, et al. Developing and Evaluating Large Language Model-Generated Emergency Medicine Handoff Notes. JAMA Netw Open. 2024 Dec 2;7(12):e2448723. PMID: 39625719. doi: 10.1001/jamanetworkopen.2024.48723.

159. Langenbach MC, Foldyna B, Hadzic I, Langenbach IL, Raghu VK, Lu MT, et al. Automated anonymization of radiology reports: comparison of publicly available natural language processing and large language models. European radiology. 2025 May;35(5):2634-41. PMID: 39480533. doi: 10.1007/s00330-024-11148-x.

160. Mori Y, Izumiyama T, Kanabuchi R, Mori N, Aizawa T. Large language model may assist diagnosis of SAPHO syndrome by bone scintigraphy. Modern rheumatology. 2024 Aug 20;34(5):1043-6. PMID: 38153762. doi: 10.1093/mr/road115.

161. Yan C, Ong HH, Grabowska ME, Krantz MS, Su WC, Dickson AL, et al. Large language models facilitate the generation of electronic health record phenotyping algorithms. Journal of the American Medical Informatics Association : JAMIA. 2024 Sep 1;31(9):1994-2001. PMID: 38613820. doi: 10.1093/jamia/ocae072.

162. Iscoe M, Socrates V, Gilson A, Chi L, Li H, Huang T, et al. Identifying signs and symptoms of urinary tract infection from emergency department clinical notes using large language models. Academic emergency medicine : official journal of the Society for Academic Emergency Medicine. 2024 Jun;31(6):599-610. PMID: 38567658. doi: 10.1111/acem.14883.

163. Zeinali N, Albashayreh A, Fan W, White SG. Symptom-BERT: Enhancing Cancer Symptom Detection in EHR Clinical Notes. J Pain Symptom Manage. 2024 Aug;68(2):190-8 e1. PMID: 38789092. doi: 10.1016/j.jpainsymman.2024.05.015.

164. Attanasio M, Mazza M, Le Donne I, Masedu F, Greco MP, Valenti M. Does ChatGPT have a typical or atypical theory of mind? Front Psychol. 2024;15:1488172. PMID: 39534470. doi: 10.3389/fpsyg.2024.1488172.

165. Brant-Zawadzki G, Klapthor B, Ryba C, Youngquist DC, Burton B, Palatinus H, et al. The Performance of ChatGPT-4 and Gemini Ultra 1.0 for Quality Assurance Review in Emergency Medical Services Chest Pain Calls. Prehospital emergency care. 2025 Jul 22;29(3):210-7. PMID: 38976859. doi: 10.1080/10903127.2024.2376757.

166. Hu D, Liu B, Zhu X, Lu X, Wu N. Zero-shot information extraction from radiological reports using ChatGPT. Int J Med Inform. 2024 Mar;183:105321. PMID: 38157785. doi: 10.1016/j.ijmedinf.2023.105321.

167. Kanemaru N, Yasaka K, Fujita N, Kanzawa J, Abe O. The Fine-Tuned Large Language Model for Extracting the Progressive Bone Metastasis from Unstructured Radiology Reports. Journal of imaging informatics in medicine. 2025 Apr;38(2):865-72. PMID: 39187702. doi: 10.1007/s10278-024-01242-3.

168. Lee KL, Kessler DA, Caglic I, Kuo YH, Shaida N, Barrett T. Assessing the performance of ChatGPT and Bard/Gemini against radiologists for Prostate Imaging-Reporting and Data System classification based on prostate multiparametric MRI text reports. Br J Radiol. 2025 Mar 1;98(1167):368-74. PMID: 39535870. doi: 10.1093/bjr/tqae236.

169. Silbergleit M, Toth A, Chamberlin JH, Hamouda M, Baruah D, Derrick S, et al. ChatGPT vs Gemini: Comparative Accuracy and Efficiency in CAD-RADS Score Assignment from Radiology Reports. Journal of imaging informatics in medicine. 2025 Aug;38(4):2303-11. PMID: 39528887. doi: 10.1007/s10278-024-01328-y.

170. Li S, Dexter P, Ben-Miled Z, Boustani M. Dementia risk prediction using decision-focused content selection from medical notes. Comput Biol Med. 2024 Nov;182:109144. PMID: 39298882. doi: 10.1016/j.compbiomed.2024.109144.

171. Huppertz MS, Siepmann R, Topp D, Nikoubashman O, Yuksel C, Kuhl CK, et al. Revolution or risk?-Assessing the potential and challenges of GPT-4V in radiologic image interpretation. European radiology. 2025 Mar;35(3):1111-21. PMID: 39422726. doi: 10.1007/s00330-024-11115-6.

172. Zhu L, Rong Y, McGee LA, Rwigema JM, Patel SH. Testing and Validation of a Custom Retrained Large Language Model for the Supportive Care of HN Patients with External Knowledge Base. Cancers. 2024 Jun 24;16(13). PMID: 39001375. doi: 10.3390/cancers16132311.

173. Ishida M, Gonoi W, Nyunoya K, Abe H, Shirota G, Okimoto N, et al. Diagnostic Performance of GPT-4o and Claude 3 Opus in Determining Causes of Death From Medical Histories and Postmortem CT Findings. Cureus. 2024 Aug;16(8):e67306. PMID: 39301343. doi: 10.7759/cureus.67306.

174. Meddeb A, Ebert P, Bressem KK, Desser D, Dell'Orco A, Bohner G, et al. Evaluating local open-source large language models for data extraction from unstructured reports on mechanical thrombectomy in patients with ischemic stroke. Journal of neurointerventional surgery. 2025 Aug 13;17(9):986-91. PMID: 39095085. doi: 10.1136/jnis-2024-022078.

175. Zhang L, Liu M, Wang L, Zhang Y, Xu X, Pan Z, et al. Constructing a Large Language Model to Generate Impressions from Findings in Radiology Reports. Radiology. 2024 Sep;312(3):e240885. PMID: 39287525. doi: 10.1148/radiol.240885.

176. Zaretsky J, Kim JM, Baskharoun S, Zhao Y, Austrian J, Aphinyanaphongs Y, et al. Generative Artificial Intelligence to Transform Inpatient Discharge Summaries to Patient-Friendly Language and Format. JAMA Netw Open. 2024 Mar 4;7(3):e240357. PMID: 38466307. doi: 10.1001/jamanetworkopen.2024.0357.

177. Yasaka K, Kanzawa J, Kanemaru N, Koshino S, Abe O. Fine-Tuned Large Language Model for Extracting Patients on Pretreatment for Lung Cancer from a Picture Archiving and Communication System Based on Radiological Reports. Journal of imaging informatics in medicine. 2025 Feb;38(1):327-34. PMID: 38955964. doi: 10.1007/s10278-024-01186-8.

178. Voinea SV, Mamuleanu M, Teica RV, Florescu LM, Selisteanu D, Gheonea IA. GPT-Driven Radiology Report Generation with Fine-Tuned Llama 3. Bioengineering (Basel, Switzerland). 2024 Oct 18;11(10). PMID: 39451418. doi: 10.3390/bioengineering11101043.

179. Matsler N, Pepin L, Banerji S, Hoyte C, Heard K. Use of large language models to optimize poison center charting. Clinical toxicology (Philadelphia, Pa). 2024 Jun;62(6):385-90. PMID: 38864738. doi: 10.1080/15563650.2024.2348107.

180. Yu Z, Peng C, Yang X, Dang C, Adekkanattu P, Gopal Patra B, et al. Identifying social determinants of health from clinical narratives: A study of performance, documentation ratio, and potential bias. J Biomed Inform. 2024 May;153:104642. PMID: 38621641. doi: 10.1016/j.jbi.2024.104642.

181. Cirkovic A, Katz T. Exploring the Potential of ChatGPT-4 in Predicting Refractive Surgery Categorizations: Comparative Study. JMIR formative research. 2023 Dec 28;7:e51798. PMID: 38153777. doi: 10.2196/51798.

182. Garcia-Agundez A, Kay JL, Li J, Gianfrancesco M, Rai B, Hu A, et al. Structuring medication signeturs as a language regression task: comparison of zero- and few-shot GPT with fine-tuned models. JAMIA open. 2024 Jul;7(2):ooae051. PMID: 38915730. doi: 10.1093/jamiaopen/ooae051.

183. Chang PW, Amini MM, Davis RO, Nguyen DD, Dodge JL, Lee H, et al. ChatGPT4 Outperforms Endoscopists for Determination of Postcolonoscopy Rescreening and Surveillance Recommendations. Clin Gastroenterol Hepatol. 2024 Sep;22(9):1917-25 e17. PMID: 38729387. doi: 10.1016/j.cgh.2024.04.022.

184. Li KD, Fernandez AM, Schwartz R, Rios N, Carlisle MN, Amend GM, et al. Comparing GPT-4 and Human Researchers in Health Care Data Analysis: Qualitative Description Study. J Med Internet Res. 2024 Aug 21;26:e56500. PMID: 39167785. doi: 10.2196/56500.

185. Wang B, Deng F, Jiang P. EEGDiR: Electroencephalogram denoising network for temporal information storage and global modeling through Retentive Network. Comput Biol Med. 2024 Jul;177:108626. PMID: 38810475. doi: 10.1016/j.compbiomed.2024.108626.

186. Dagli MM, Ghenbot Y, Ahmad HS, Chauhan D, Turlip R, Wang P, et al. Development and validation of a novel AI framework using NLP with LLM integration for relevant clinical data extraction through automated chart review. Sci Rep. 2024 Nov 5;14(1):26783. PMID: 39500759. doi: 10.1038/s41598-024-77535-y.

187. Fervers P, Hahnfeldt R, Kottlors J, Wagner A, Maintz D, Pinto Dos Santos D, et al. ChatGPT yields low accuracy in determining LI-RADS scores based on free-text and structured radiology reports in German language. Frontiers in radiology. 2024;4:1390774. PMID: 39036542. doi: 10.3389/fradi.2024.1390774.

188. Kim S, Warner BC, Lew D, Lou SS, Kannampallil T. Measuring cognitive effort using tabular transformer-based language models of electronic health record-based audit log action sequences. Journal of the American Medical Informatics Association : JAMIA. 2024 Oct 1;31(10):2228-35. PMID: 39001791. doi: 10.1093/jamia/ocae171.

189. Carl N, Nguyen L, Haggenmuller S, Joachim Hetz M, Theres Winterstein J, Otto Hartung F, et al. Comparing Patient's Confidence in Clinical Capabilities in Urology: Large Language Models Versus Urologists. European urology open science. 2024 Dec;70:91-8. PMID: 39507511. doi: 10.1016/j.euros.2024.10.009.

190. Chien A, Tang H, Jagessar B, Chang KW, Peng N, Nael K, et al. AI-Assisted Summarization of Radiologic Reports: Evaluating GPT3davinci, BARTcnn, LongT5booksum, LEDbooksum, LEDlegal, and LEDclinical. AJNR American journal of neuroradiology. 2024 Feb 7;45(2):244-8. PMID: 38238092. doi: 10.3174/ajnr.A8102.

191. Chen CC, Wei CJ, Tseng TY, Chiu MC, Chang CC. Applying Object Detection and Large Language Model to Establish a Smart Telemedicine Diagnosis System with Chatbot: A Case Study of Pressure Injuries Diagnosis System. Telemedicine journal and e-health : the official journal of the American Telemedicine Association. 2024 Jun;30(6):e1705-e12. PMID: 38512470. doi: 10.1089/tmj.2023.0715.

192. Lee JE, Park KS, Kim YH, Song HC, Park B, Jeong YJ. Lung Cancer Staging Using Chest CT and FDG PET/CT Free-Text Reports: Comparison Among Three ChatGPT Large Language Models and Six Human Readers of Varying Experience. AJR American journal of roentgenology. 2024 Dec;223(6):e2431696. PMID: 39230409. doi: 10.2214/AJR.24.31696.

193. Cho H, Yoo S, Kim B, Jang S, Sunwoo L, Kim S, et al. Extracting lung cancer staging descriptors from pathology reports: A generative language model approach. J Biomed Inform. 2024 Sep;157:104720. PMID: 39233209. doi: 10.1016/j.jbi.2024.104720.

194. Hsueh JY, Nethala D, Singh S, Linehan WM, Ball MW. Investigating the clinical reasoning abilities of large language model GPT-4: an analysis of postoperative complications from renal surgeries. Urologic oncology. 2024 Sep;42(9):292 e1- e7. PMID: 38714380. doi: 10.1016/j.urolonc.2024.04.010.

195. Akhondi-Asl A, Yang Y, Luchette M, Burns JP, Mehta NM, Geva A. Comparing the Quality of Domain-Specific Versus General Language Models for Artificial Intelligence-Generated Differential Diagnoses in PICU Patients. Pediatric critical care medicine : a journal of the Society of Critical Care Medicine and the World Federation of Pediatric Intensive and Critical Care Societies. 2024 Jun 1;25(6):e273-e82. PMID: 38329382. doi: 10.1097/PCC.0000000000003468.

196. Aubreville M, Ganz J, Ammeling J, Rosbach E, Gehrke T, Scherzad A, et al. Prediction of tumor board procedural recommendations using large language models. Eur Arch Otorhinolaryngol. 2025 Mar;282(3):1619-29. PMID: 39266750. doi: 10.1007/s00405-024-08947-9.

197. Siepmann R, Huppertz M, Rastkhiz A, Reen M, Corban E, Schmidt C, et al. The virtual reference radiologist: comprehensive AI assistance for clinical image reading and interpretation. European radiology. 2024 Oct;34(10):6652-66. PMID: 38627289. doi: 10.1007/s00330-024-10727-2.

198. Wang X, Ye S, Feng J, Feng K, Yang H, Li H. Performance of ChatGPT on prehospital acute ischemic stroke and large vessel occlusion (LVO) stroke screening. Digit Health. 2024 Jan-Dec;10:20552076241297127. PMID: 39507012. doi: 10.1177/20552076241297127.

199. Kim H, Jin HM, Jung YB, You SC. Patient-Friendly Discharge Summaries in Korea Based on ChatGPT: Software Development and Validation. Journal of Korean medical science. 2024 Apr 29;39(16):e148. PMID: 38685890. doi: 10.3346/jkms.2024.39.e148.

200. Elek A, Ekizalioglu DD, Guler E. Evaluating Microsoft Bing with ChatGPT-4 for the assessment of abdominal computed tomography and magnetic resonance images. Diagnostic and interventional radiology (Ankara, Turkey). 2025 Apr 28;31(3):196-205. PMID: 39155793. doi: 10.4274/dir.2024.232680.

201. Bhayana R, Nanda B, Dehkharghanian T, Deng Y, Bhambra N, Elias G, et al. Large Language Models for Automated Synoptic Reports and Resectability Categorization in Pancreatic Cancer. Radiology. 2024 Jun;311(3):e233117. PMID: 38888478. doi: 10.1148/radiol.233117.

202. Zaidat B, Lahoti YS, Yu A, Mohamed KS, Cho SK, Kim JS. Artificially Intelligent Billing in Spine Surgery: An Analysis of a Large Language Model. Global spine journal. 2025 Mar;15(2):1113-20. PMID: 38147047. doi: 10.1177/21925682231224753.

203. Tan R, Lin Q, Low GH, Lin R, Goh TC, Chang CCE, et al. Inferring cancer disease response from radiology reports using large language models with data augmentation and prompting. Journal of the American Medical Informatics Association : JAMIA. 2023 Sep 25;30(10):1657-64. PMID: 37451682. doi: 10.1093/jamia/ocad133.

204. Cozzi A, Pinker K, Hidber A, Zhang T, Bonomo L, Lo Gullo R, et al. BI-RADS Category Assignments by GPT-3.5, GPT-4, and Google Bard: A Multilanguage Study. Radiology. 2024 Apr;311(1):e232133. PMID: 38687216. doi: 10.1148/radiol.232133.

205. Li Y, Zheng X, Li J, Dai Q, Wang CD, Chen M. LKAN: LLM-Based Knowledge-Aware Attention Network for Clinical Staging of Liver Cancer. IEEE J Biomed Health Inform. 2025 Apr;29(4):3007-20. PMID: 39392729. doi: 10.1109/JBHI.2024.3478809.

206. Carla MM, Gambini G, Baldascino A, Boselli F, Giannuzzi F, Margollicci F, et al. Large language models as assistance for glaucoma surgical cases: a ChatGPT vs. Google Gemini comparison. Graefe's archive for clinical and experimental ophthalmology = Albrecht von Graefes Archiv fur klinische und experimentelle Ophthalmologie. 2024 Sep;262(9):2945-59. PMID: 38573349. doi: 10.1007/s00417-024-06470-5.

207. Liu X, Wu J, Shao A, Shen W, Ye P, Wang Y, et al. Uncovering Language Disparity of ChatGPT on Retinal Vascular Disease Classification: Cross-Sectional Study. J Med Internet Res. 2024 Jan 22;26:e51926. PMID: 38252483. doi: 10.2196/51926.

208. Uranbey O, Ozbey F, Kaygisiz O, Ayranci F. Assessing ChatGPT's Diagnostic Accuracy and Therapeutic Strategies in Oral Pathologies: A Cross-Sectional Study. Cureus. 2024 Apr;16(4):e58607. PMID: 38770501. doi: 10.7759/cureus.58607.

209. Dronkers EAC, Geneid A, Al Yaghchi C, Lechien JR. Evaluating the Potential of AI Chatbots in Treatment Decision-making for Acquired Bilateral Vocal Fold Paralysis in Adults. Journal of voice : official journal of the Voice Foundation. 2025 Jul;39(4):871-81. PMID: 38584026. doi: 10.1016/j.jvoice.2024.02.020.

210. Chiang CC, Luo M, Dumkrieger G, Trivedi S, Chen YC, Chao CJ, et al. A large language model-based generative natural language processing framework fine-tuned on clinical notes accurately extracts headache frequency from electronic health records. Headache. 2024 Apr;64(4):400-9. PMID: 38525734. doi: 10.1111/head.14702.

211. Lehnen NC, Dorn F, Wiest IC, Zimmermann H, Radbruch A, Kather JN, et al. Data Extraction from Free-Text Reports on Mechanical Thrombectomy in Acute Ischemic Stroke Using ChatGPT: A Retrospective Analysis. Radiology. 2024 Apr;311(1):e232741. PMID: 38625006. doi: 10.1148/radiol.232741.

212. Wang Z, Zhang Z, Traverso A, Dekker A, Qian L, Sun P. Assessing the role of GPT-4 in thyroid ultrasound diagnosis and treatment recommendations: enhancing interpretability with a chain of thought approach. Quantitative imaging in medicine and surgery. 2024 Feb 1;14(2):1602-15. PMID: 38415150. doi: 10.21037/qims-23-1180.

213. Tayebi Arasteh S, Siepmann R, Huppertz M, Lotfinia M, Puladi B, Kuhl C, et al. The Treasure Trove Hidden in Plain Sight: The Utility of GPT-4 in Chest Radiograph Evaluation. Radiology. 2024 Nov;313(2):e233441. PMID: 39530893. doi: 10.1148/radiol.233441.

214. Al Tibi G, Alexander M, Miller S, Chronos N. A Retrospective Comparison of Medication Recommendations Between a Cardiologist and ChatGPT-4 for Hypertension Patients in a Rural Clinic. Cureus. 2024 Mar;16(3):e55789. PMID: 38586651. doi: 10.7759/cureus.55789.

215. Rezaii N, Hochberg D, Quimby M, Wong B, Brickhouse M, Touroutoglou A, et al. Artificial intelligence classifies primary progressive aphasia from connected speech. Brain : a journal of neurology. 2024 Sep 3;147(9):3070-82. PMID: 38912855. doi: 10.1093/brain/awae196.

216. Barash Y, Klang E, Konen E, Sorin V. ChatGPT-4 Assistance in Optimizing Emergency Department Radiology Referrals and Imaging Selection. Journal of the American College of Radiology : JACR. 2023 Oct;20(10):998-1003. PMID: 37423350. doi: 10.1016/j.jacr.2023.06.009.

217. Pagano S, Holzapfel S, Kappenschneider T, Meyer M, Maderbacher G, Grifka J, et al. Arthrosis diagnosis and treatment recommendations in clinical practice: an exploratory investigation with the generative AI model GPT-4. Journal of orthopaedics and traumatology : official journal of the Italian Society of Orthopaedics and Traumatology. 2023 Nov 28;24(1):61. PMID: 38015298. doi: 10.1186/s10195-023-00740-4.

218. Strotzer QD, Nieberle F, Kupke LS, Napodano G, Muertz AK, Meiler S, et al. Toward Foundation Models in Radiology? Quantitative Assessment of GPT-4V's Multimodal and Multianatomic Region Capabilities. Radiology. 2024 Nov;313(2):e240955. PMID: 39589253. doi: 10.1148/radiol.240955.

219. Hasani AM, Singh S, Zahergivar A, Ryan B, Nethala D, Bravomontenegro G, et al. Evaluating the performance of Generative Pre-trained Transformer-4 (GPT-4) in standardizing radiology reports. European radiology. 2024 Jun;34(6):3566-74. PMID: 37938381. doi: 10.1007/s00330-023-10384-x.

220. Scroggins JK, Topaz M, Song J, Zolnoori M. Does synthetic data augmentation improve the performances of machine learning classifiers for identifying health problems in patient-nurse verbal communications in home healthcare settings? Journal of nursing scholarship : an official publication of Sigma Theta Tau International Honor Society of Nursing. 2025 Jan;57(1):47-58. PMID: 38961517. doi: 10.1111/jnu.13004.

221. Baumgartner K, Byczkowski M, Schmid T, Muschko M, Woessner P, Gerlach A, et al. Effectiveness of the Medical Chatbot PROSCA to Inform Patients About Prostate Cancer: Results of a Randomized Controlled Trial. European urology open science. 2024 Nov;69:80-8. PMID: 39329071. doi: 10.1016/j.euros.2024.08.022.

222. Woo KC, Simon GW, Akindutire O, Aphinyanaphongs Y, Austrian JS, Kim JG, et al. Evaluation of GPT-4 ability to identify and generate patient instructions for actionable incidental radiology findings. Journal of the American Medical Informatics Association : JAMIA. 2024 Sep 1;31(9):1983-93. PMID: 38778578. doi: 10.1093/jamia/ocae117.

223. Schmidl B, Hutten T, Pigorsch S, Stogbauer F, Hoch CC, Hussain T, et al. Assessing the use of the novel tool Claude 3 in comparison to ChatGPT 4.0 as an artificial intelligence tool in the diagnosis and therapy of primary head and neck cancer cases. Eur Arch Otorhinolaryngol. 2024 Nov;281(11):6099-109. PMID: 39112556. doi: 10.1007/s00405-024-08828-1.

224. Zhang J, Ma Y, Zhang R, Chen Y, Xu M, Rina S, et al. A comparative study of GPT-4o and human ophthalmologists in glaucoma diagnosis. Sci Rep. 2024 Dec 5;14(1):30385. PMID: 39639068. doi: 10.1038/s41598-024-80917-x.

225. Ali A, Kumar RP, Polavarapu H, Lavadi RS, Mahavadi A, Legarreta AD, et al. Bridging the Gap: Can Large Language Models Match Human Expertise in Writing Neurosurgical Operative Notes? World neurosurgery. 2024 Dec;192:e34-e41. PMID: 39153569. doi: 10.1016/j.wneu.2024.08.062.

226. Abu-Ashour W, Emil S, Poenaru D. Using Artificial Intelligence to Label Free-Text Operative and Ultrasound Reports for Grading Pediatric Appendicitis. Journal of pediatric surgery. 2024 May;59(5):783-90. PMID: 38383177. doi: 10.1016/j.jpedsurg.2024.01.033.

227. Gengatharan D, Saggi SS, Bin Abd Razak HR. Pre-operative Planning of High Tibial Osteotomy With ChatGPT: Are We There Yet? Cureus. 2024 Feb;16(2):e54858. PMID: 38533173. doi: 10.7759/cureus.54858.

228. Lukac S, Dayan D, Fink V, Leinert E, Hartkopf A, Veselinovic K, et al. Evaluating ChatGPT as an adjunct for the multidisciplinary tumor board decision-making in primary breast cancer cases. Archives of gynecology and obstetrics. 2023 Dec;308(6):1831-44. PMID: 37458761. doi: 10.1007/s00404-023-07130-5.

229. Sievert M, Aubreville M, Mueller SK, Eckstein M, Breininger K, Iro H, et al. Diagnosis of malignancy in oropharyngeal confocal laser endomicroscopy using GPT 4.0 with vision. Eur Arch Otorhinolaryngol. 2024 Apr;281(4):2115-22. PMID: 38329525. doi: 10.1007/s00405-024-08476-5.

230. Bhattarai K, Oh IY, Sierra JM, Tang J, Payne PRO, Abrams Z, et al. Leveraging GPT-4 for identifying cancer phenotypes in electronic health records: a performance comparison between GPT-4, GPT-3.5-turbo, Flan-T5, Llama-3-8B, and spaCy's rule-based and machine learning-based methods. JAMIA open. 2024 Oct;7(3):ooae060. PMID: 38962662. doi: 10.1093/jamiaopen/ooae060.

231. Mitsuyama Y, Tatekawa H, Takita H, Sasaki F, Tashiro A, Oue S, et al. Comparative analysis of GPT-4-based ChatGPT's diagnostic performance with radiologists using real-world radiology reports of brain tumors. European radiology. 2025 Apr;35(4):1938-47. PMID: 39198333. doi: 10.1007/s00330-024-11032-8.

232. van Nuland M, Snoep JD, Egberts T, Erdogan A, Wassink R, van der Linden PD. Poor performance of ChatGPT in clinical rule-guided dose interventions in hospitalized patients with renal dysfunction. European journal of clinical pharmacology. 2024 Aug;80(8):1133-40. PMID: 38592470. doi: 10.1007/s00228-024-03687-5.

233. Rosen S, Saban M. Evaluating the reliability of ChatGPT as a tool for imaging test referral: a comparative study with a clinical decision support system. European radiology. 2024 May;34(5):2826-37. PMID: 37828297. doi: 10.1007/s00330-023-10230-0.

234. Marshall C, Forbes J, Seidman MD, Roldan L, Atkins J. Artificial Intelligence for Diagnosis in Otologic Patients: Is It Ready to Be Your Doctor? Otology & neurotology : official publication of the American Otological Society, American Neurotology Society [and] European Academy of Otology and Neurotology. 2024 Sep 1;45(8):863-9. PMID: 39142308. doi: 10.1097/MAO.0000000000004267.

235. Scott M, Muncey W, Seranio N, Belladelli F, Del Giudice F, Li S, et al. Assessing Artificial Intelligence-Generated Responses to Urology Patient In-Basket Messages. Urology practice. 2024 Sep;11(5):793-8. PMID: 39162591. doi: 10.1097/UPJ.0000000000000637.

236. Tastan Eroglu Z, Babayigit O, Ozkan Sen D, Ucan Yarkac F. Performance of ChatGPT in classifying periodontitis according to the 2018 classification of periodontal diseases. Clinical oral investigations. 2024 Jun 29;28(7):407. PMID: 38951256. doi: 10.1007/s00784-024-05799-9.

237. Pedro T, Sousa JM, Fonseca L, Gama MG, Moreira G, Pintalhao M, et al. Exploring the use of ChatGPT in predicting anterior circulation stroke functional outcomes after mechanical thrombectomy: a pilot study. Journal of neurointerventional surgery. 2025 Feb 14;17(3):261-5. PMID: 38453462. doi: 10.1136/jnis-2024-021556.

238. Agaronnik ND, Davis J, Manz CR, Tulsky JA, Lindvall C. Large Language Models to Identify Advance Care Planning in Patients With Advanced Cancer. J Pain Symptom Manage. 2025 Mar;69(3):243-50 e1. PMID: 39586429. doi: 10.1016/j.jpainsymman.2024.11.016.

239. Baxter SL, Longhurst CA, Millen M, Sitapati AM, Tai-Seale M. Generative artificial intelligence responses to patient messages in the electronic health record: early lessons learned. JAMIA open. 2024 Jul;7(2):ooae028. PMID: 38601475. doi: 10.1093/jamiaopen/ooae028.

240. Gupta A, Malhotra H, Garg AK, Rangarajan K. Enhancing Radiological Reporting in Head and Neck Cancer: Converting Free-Text CT Scan Reports to Structured Reports Using Large Language Models. The Indian journal of radiology & imaging. 2025 Jan;35(1):43-9. PMID: 39697521. doi: 10.1055/s-0044-1788589.

241. Gupta A, Rastogi A, Malhotra H, Rangarajan K. Comparative Evaluation of Large Language Models for Translating Radiology Reports into Hindi. The Indian journal of radiology & imaging. 2025 Jan;35(1):88-96. PMID: 39697509. doi: 10.1055/s-0044-1789618.

242. Hooshangnejad H, Huang G, Kelly K, Feng X, Luo Y, Zhang R, et al. EXACT-Net: Framework for EHR-Guided Lung Tumor Auto-Segmentation for Non-Small Cell Lung Cancer Radiotherapy. Cancers. 2024 Dec 6;16(23). PMID: 39682283. doi: 10.3390/cancers16234097.

243. Schwieger A, Angst K, de Bardeci M, Burrer A, Cathomas F, Ferrea S, et al. Large language models can support generation of standardized discharge summaries - A retrospective study utilizing ChatGPT-4 and electronic health records. Int J Med Inform. 2024 Dec;192:105654. PMID: 39437512. doi: 10.1016/j.ijmedinf.2024.105654.

244. Alkhalaf M, Yu P, Yin M, Deng C. Applying generative AI with retrieval augmented generation to summarize and extract key clinical information from electronic health records. J Biomed Inform. 2024 Aug;156:104662. PMID: 38880236. doi: 10.1016/j.jbi.2024.104662.

245. Obuseh M, Singh S, Anton NE, Gardiner R, Stefanidis D, Yu D. Feasibility of large language models for assessing and coaching surgeons' non-technical skills. npj health systems. 2025;2(1):25. PMID: 40678790. doi: 10.1038/s44401-025-00027-2.

246. Kang C, Li J, Yang X, Ren G, Zhang L, Wang W, et al. Performance of large language models in the differential diagnosis of benign and malignant biliary stricture. Front Oncol. 2025;15:1613818. PMID: 40678070. doi: 10.3389/fonc.2025.1613818.

247. Radulesco T, Ebode D, Maniaci A, Gargula S, Saibene AM, Chiesa-Estomba C, et al. Evaluation of Artificial Intelligence Chatbots for Facial Injection Planning: Comparative Performance and Safety Limitations. Aesthetic Plast Surg. 2025 Jul 16. PMID: 40670654. doi: 10.1007/s00266-025-05010-8.

248. Del Monte F, Barolo R, Circhetta M, Delmonaco AG, Castagno E, Pivetta E, et al. Diagnostic efficacy of large language models in the pediatric emergency department: a pilot study. Frontiers in digital health. 2025;7:1624786. PMID: 40666107. doi: 10.3389/fdgth.2025.1624786.

249. Wang P, Liu Z, Li Y, Holmes J, Shu P, Zhang L, et al. Fine-tuning open-source large language models to improve their performance on radiation oncology tasks: A feasibility study to investigate their potential clinical applications in radiation oncology. Med Phys. 2025 Jul;52(7):e17985. PMID: 40665561. doi: 10.1002/mp.17985.

250. Seifen C, Huppertz T, Bahr-Hamm K, Gouveris H, Pordzik J, Eckrich J, et al. Evaluating Locally Run Large Language Models for Obstructive Sleep Apnea Diagnosis and Treatment: A Real-World Polysomnography Study. Nature and science of sleep. 2025;17:1587-99. PMID: 40661856. doi: 10.2147/NSS.S536823.

251. Gao H, Wang K, Yuan Y, Wang Y, Liu Q, Wang Y, et al. A large language model based pipeline for extracting information from patient complaint and anamnesis in clinical notes for severity assessment. Sci Rep. 2025 Jul 14;15(1):25345. PMID: 40659653. doi: 10.1038/s41598-025-07649-4.

252. McCoy TH, Perlis RH. Applying large language models to stratify suicide risk using narrative clinical notes. Journal of mood and anxiety disorders. 2025 Jun;10:100109. PMID: 40657592. doi: 10.1016/j.xjmad.2025.100109.

253. Tassone DM, Hitchcock MM, Rossier CJ, Fletcher D, Ye J, Langford I, et al. Evaluating chain-of-thought prompting in a GPT chatbot for BCID2 interpretation and stewardship: how does AI compare to human experts? Antimicrobial stewardship & healthcare epidemiology : ASHE. 2025;5(1):e154. PMID: 40657035. doi: 10.1017/ash.2025.10059.

254. McCoy TH, Perlis RH. Characterizing research domain criteria symptoms among psychiatric inpatients using large language models. Journal of mood and anxiety disorders. 2024 Dec;8:100079. PMID: 40655912. doi: 10.1016/j.xjmad.2024.100079.

255. Chen Z, Chambara N, Liu SYW, Chow TCM, Lai CMS, Ying MTC. Exploring the Potential of ChatGPT-4o in Thyroid Nodule Diagnosis Using Multi-Modality Ultrasound Imaging: Dual- vs. Triple-Modality Approaches. Cancers. 2025 Jun 20;17(13). PMID: 40647374. doi: 10.3390/cancers17132068.

256. Chan LYT, Chan DZM, Tan YL, Yap QV, Ong W, Lee A, et al. Evaluating the Accuracy of Privacy-Preserving Large Language Models in Calculating the Spinal Instability Neoplastic Score (SINS). Cancers. 2025 Jun 20;17(13). PMID: 40647373. doi: 10.3390/cancers17132073.

257. Fang S, Holgate B, Shek A, Winston JS, McWilliam M, Viana PF, et al. Extracting epilepsy-related information from unstructured clinic letters using large language models. Epilepsia. 2025 Jul 10. PMID: 40637590. doi: 10.1111/epi.18475.

258. Choubey AP, Eguia E, Hollingsworth A, Chatterjee S, D'Angelica MI, Jarnagin WR, et al. Data Extraction and Curation from Radiology Reports for Pancreatic Cyst Surveillance Using Large Language Models. J Am Coll Surg. 2025 Jul 10. PMID: 40637302. doi: 10.1097/XCS.0000000000001478.

259. Kong M, Fernandez A, Bains J, Milisavljevic A, Brooks KC, Shanmugam A, et al. Evaluation of the accuracy and safety of machine translation of patient-specific discharge instructions: a comparative analysis. BMJ quality & safety. 2025 Jul 9. PMID: 40633961. doi: 10.1136/bmjqs-2024-018384.

260. Balch JA, Desaraju SS, Nolan VJ, Vellanki D, Buchanan TR, Brinkley LM, et al. Language Models for Multilabel Document Classification of Surgical Concepts in Exploratory Laparotomy Operative Notes: Algorithm Development Study. JMIR Med Inform. 2025 Jul 9;13:e71176. PMID: 40632815. doi: 10.2196/71176.

261. Ramamurthi A, Neupane B, Deshpande P, Hanson R, Vegesna S, Cray D, et al. Applying Large Language Models for Surgical Case Length Prediction. JAMA surgery. 2025 Aug 1;160(8):894-902. PMID: 40632526. doi: 10.1001/jamasurg.2025.2154.

262. Guler R, Yalcin E. Performance of AI Chatbots in Preliminary Diagnosis of Maxillofacial Pathologies. Medical science monitor : international medical journal of experimental and clinical research. 2025 Jul 9;31:e949076. PMID: 40629684. doi: 10.12659/MSM.949076.

263. Ray M, Kats DJ, Moorkens J, Rai D, Shaar N, Quinones D, et al. Evaluating a Large Language Model in Translating Patient Instructions to Spanish Using a Standardized Framework. JAMA Pediatr. 2025 Jul 7. PMID: 40622720. doi: 10.1001/jamapediatrics.2025.1729.

264. Shi MJ, Wang ZX, Wang SK, Li XH, Zhang YL, Yan Y, et al. Performance of GPT-4 for automated prostate biopsy decision-making based on mpMRI: a multi-center evidence study. Mil Med Res. 2025 Jul 7;12(1):33. PMID: 40619425. doi: 10.1186/s40779-025-00621-3.

265. Yang X, Li L, Wang C, Zhang W, Liu H, Tang W. Leveraging heterogeneous tabular of EHRs with prompt learning for clinical prediction. J Biomed Inform. 2025 Aug;168:104868. PMID: 40619074. doi: 10.1016/j.jbi.2025.104868.

266. Yao G, Zhang W, Zhu Y, Wong UK, Zhang Y, Yang C, et al. Comparing the accuracy of large language models and prompt engineering in diagnosing realworld cases. Int J Med Inform. 2025 Nov;203:106026. PMID: 40617017. doi: 10.1016/j.ijmedinf.2025.106026.

267. Amini M, Chang PW, Davis RO, Nguyen DD, Dodge JL, Phan J, et al. Comparing ChatGPT3.5 and Bard recommendations for colonoscopy intervals: Bridging the gap in healthcare settings. Endoscopy international open. 2025;13:a25865912. PMID: 40611845. doi: 10.1055/a-2586-5912.

268. Sing DC, Shah KS, Pompliano M, Yi PH, Velluto C, Bagheri A, et al. Enhancing Magnetic Resonance Imaging (MRI) Report Comprehension in Spinal Trauma: Readability Analysis of AI-Generated Explanations for Thoracolumbar Fractures. Jmir ai. 2025 Jul 1;4:e69654. PMID: 40611700. doi: 10.2196/69654.

269. Bartels S, Carus J. From text to data: Open-source large language models in extracting cancer related medical attributes from German pathology reports. Int J Med Inform. 2025 Nov;203:106022. PMID: 40609461. doi: 10.1016/j.ijmedinf.2025.106022.

270. Garcia-Carmona AM, Prieto ML, Puertas E, Beunza JJ. Leveraging Large Language Models for Accurate Retrieval of Patient Information From Medical Reports: Systematic Evaluation Study. Jmir ai. 2025 Jul 3;4:e68776. PMID: 40608403. doi: 10.2196/68776.

271. Xu Z, Ye J, Chen J, Zhang X, Wang J. Using ChatGPT to assist in judging the indications for emergency ultrasound: an innovative exploration of optimizing medical resource allocation. Front Med (Lausanne). 2025;12:1567608. PMID: 40606467. doi: 10.3389/fmed.2025.1567608.

272. AlFarabi Ali S, AlDehlawi H, Jazzar A, Ashi H, Esam Abuzinadah N, AlOtaibi M, et al. The Diagnostic Performance of Large Language Models and Oral Medicine Consultants for Identifying Oral Lesions in Text-Based Clinical Scenarios: Prospective Comparative Study. Jmir ai. 2025 Apr 24;4:e70566. PMID: 40605790. doi: 10.2196/70566.

273. Doremus O, Russon D, Contrand B, Guerra-Adames A, Avalos-Fernandez M, Gil-Jardine C, et al. Harnessing Moderate-Sized Language Models for Reliable Patient Data Deidentification in Emergency Department Records: Algorithm Development, Validation, and Implementation Study. Jmir ai. 2025 Apr 1;4:e57828. PMID: 40605780. doi: 10.2196/57828.

274. Zhu M, Lin H, Jiang J, Jinia AJ, Jee J, Pichotta K, et al. Large language model trained on clinical oncology data predicts cancer progression. NPJ Digit Med. 2025 Jul 2;8(1):397. PMID: 40604229. doi: 10.1038/s41746-025-01780-2.

275. Isaradech N, Riedel A, Sirikul W, Kreuzthaler M, Schulz S. Zero- and few-shot Named Entity Recognition and Text Expansion in medication prescriptions using large language models. Artif Intell Med. 2025 Sep;167:103165. PMID: 40602231. doi: 10.1016/j.artmed.2025.103165.

276. Satheakeerthy S, Jesudason D, Bahrami B, Bacchi S, Lee YM, Casson R, et al. Zero-shot LLM-based visual acuity extraction: a pilot study. BMC ophthalmology. 2025 Jul 1;25(1):359. PMID: 40597830. doi: 10.1186/s12886-025-04193-7.

277. Zhang Z, Scroggins JK, Harkins S, Hulchafo, II, Moen H, Tadiello M, et al. Toward equitable documentation: Evaluating ChatGPT's role in identifying and rephrasing stigmatizing language in electronic health records. Nursing outlook. 2025 Jul-Aug;73(4):102472. PMID: 40596800. doi: 10.1016/j.outlook.2025.102472.

278. Kavak EE, Erdat EC, Altundag Derin Z, Dilli I, Kubilay Tolunay P, Oksuzoglu B, et al. Comparison of AI chatbot predicted and realworld survival outcomes in hepatocellular carcinoma. Sci Rep. 2025 Jul 1;15(1):21728. PMID: 40596129. doi: 10.1038/s41598-025-06591-9.

279. Sharabiani M, Mahani A, Bottle A, Srinivasan Y, Issitt R, Stoica S. GenAI exceeds clinical experts in predicting acute kidney injury following paediatric cardiopulmonary bypass. Sci Rep. 2025 Jul 1;15(1):20847. PMID: 40593923. doi: 10.1038/s41598-025-04651-8.

280. Hsu CC, Obermeyer Z, Tan C. A machine learning model using clinical notes to identify physician fatigue. Nat Commun. 2025 Jul 1;16(1):5791. PMID: 40592818. doi: 10.1038/s41467-025-60865-4.

281. Hopkins BS, Dallas J, Yu J, Briggs RG, Chung LK, Cote DJ, et al. The use of generative artificial intelligence-based dictation in a neurosurgical practice: a pilot study. Neurosurgical focus. 2025 Jul 1;59(1):E8. PMID: 40591970. doi: 10.3171/2025.4.FOCUS24834.

282. Tariq A, Sikha M, Kurian AW, Ward K, Keegan THM, Rubin DL, et al. Open-Source Hybrid Large Language Model Integrated System for Extraction of Breast Cancer Treatment Pathway From Free-Text Clinical Notes. JCO clinical cancer informatics. 2025 Jun;9:e2500002. PMID: 40577660. doi: 10.1200/CCI-25-00002.

283. Bootsma-Robroeks C, Workum JD, Schuit SCE, Hoekman A, Mehri T, Doornberg JN, et al. AI-generated draft replies to patient messages: exploring effects of implementation. Frontiers in digital health. 2025;7:1588143. PMID: 40575383. doi: 10.3389/fdgth.2025.1588143.

284. Yildirim A, Cicek O, Genc YS. Can AI-Based ChatGPT Models Accurately Analyze Hand-Wrist Radiographs? A Comparative Study. Diagnostics (Basel, Switzerland). 2025 Jun 14;15(12). PMID: 40564836. doi: 10.3390/diagnostics15121513.

285. Di Palma L, Darvizeh F, Ali M, Fazzini D. Structured Transformation of Unstructured Prostate MRI Reports Using Large Language Models. Tomography (Ann Arbor, Mich). 2025 Jun 17;11(6). PMID: 40560015. doi: 10.3390/tomography11060069.

286. Erdem TE, Kirilmaz A, Kekec AF. Diagnostic Performance of ChatGPT-4o in Detecting Hip Fractures on Pelvic X-rays. Cureus. 2025 Jun;17(6):e86654. PMID: 40557058. doi: 10.7759/cureus.86654.

287. Chang YC, Hsiao SH, Yeh WC, Hsing YC, Wang CC, Chen CY. Extracting critical clinical indicators and survival prediction of lung cancer from pathology reports using large language models. Comput Biol Med. 2025 Sep;195:110621. PMID: 40554977. doi: 10.1016/j.compbiomed.2025.110621.

288. Arzideh K, Schafer H, Allende-Cid H, Baldini G, Hilser T, Idrissi-Yaghir A, et al. From BERT to generative AI - Comparing encoder-only vs. large language models in a cohort of lung cancer patients for named entity recognition in unstructured medical reports. Comput Biol Med. 2025 Sep;195:110665. PMID: 40554973. doi: 10.1016/j.compbiomed.2025.110665.

289. Pan Y, Tian S, Guo J, Cai H, Wan J, Fang C. Clinical feasibility of AI Doctors: Evaluating the replacement potential of large language models in outpatient settings for central nervous system tumors. Int J Med Inform. 2025 Nov;203:106013. PMID: 40554367. doi: 10.1016/j.ijmedinf.2025.106013.

290. Lee T, Kim H, Park SH, Chae S, Yoon SH. Evaluation of Vision-Language Models for Detection and Deidentification of Medical Images with Burned-In Protected Health Information. Radiology. 2025 Jun;315(3):e243664. PMID: 40552998. doi: 10.1148/radiol.243664.

291. Gao X, Zhao L, Zhang Q, Guo Z, Hu W, Tang W, et al. Multimodal Language Model for Jaw Osteonecrosis Diagnosis and Treatment. J Dent Res. 2025 Jun 24:220345251334575. PMID: 40552509. doi: 10.1177/00220345251334575.

292. Sanli DET, Sanli AN, Buyukdereli Atadag Y, Kurt A, Esmerer E. GPT-4o and Specialized AI in Breast Ultrasound Imaging: A comparative Study on Accuracy, Agreement, Limitations, and Diagnostic Potential. Journal of ultrasound in medicine : official journal of the American Institute of Ultrasound in Medicine. 2025 Jun 23. PMID: 40548624. doi: 10.1002/jum.16749.

293. Suarez A, Freire Y, Suarez M, Diaz-Flores Garcia V, Andreu-Vazquez C, Thuissard Vasallo IJ, et al. Diagnostic Performance of Multimodal Large Language Models in the Analysis of Oral Pathology. Oral Dis. 2025 Jun 22. PMID: 40545674. doi: 10.1111/odi.70009.

294. Li Y, Li F, Hong N, Li M, Roberts K, Cui L, et al. A comparative study of recent large language models on generating hospital discharge summaries for lung cancer patients. J Biomed Inform. 2025 Aug;168:104867. PMID: 40544901. doi: 10.1016/j.jbi.2025.104867.

295. Karino M, So C, Jinta T, Tanaka M, Nakamura T, Okafuji K, et al. Exploring the Potential of ChatGPT for the Summarization of Patient Medical Histories: A Pilot Study. Cureus. 2025 May;17(5):e84133. PMID: 40525027. doi: 10.7759/cureus.84133.

296. Bernstein AM, Janeke P, Riggs RV, Burke E, Meyer J, Moyer MF, et al. Artificial Intelligence-Based Hospital Malnutrition Screening: Validation of a Novel Machine Learning Model. Appl Clin Inform. 2025 Jun 16. PMID: 40523638. doi: 10.1055/a-2635-3158.

297. Gui X, Lv H, Wang X, Lv L, Xiao Y, Wang L. Enhancing hepatopathy clinical trial efficiency: a secure, large language model-powered pre-screening pipeline. BioData Min. 2025 Jun 14;18(1):42. PMID: 40517253. doi: 10.1186/s13040-025-00458-5.

298. Wang H, Yang R, Alwakeel M, Kayastha A, Chowdhury A, Biro JM, et al. An evaluation framework for ambient digital scribing tools in clinical applications. NPJ Digit Med. 2025 Jun 13;8(1):358. PMID: 40514413. doi: 10.1038/s41746-025-01622-1.

299. Imaezue GC, Marampelly H. ABCD: A Simulation Method for Accelerating Conversational Agents With Applications in Aphasia Therapy. Journal of speech, language, and hearing research : JSLHR. 2025 Jul 8;68(7):3322-36. PMID: 40512969. doi: 10.1044/2025_JSLHR-25-00003.

300. Li R, Mao S, Zhu C, Yang Y, Tan C, Li L, et al. Enhancing Pulmonary Disease Prediction Using Large Language Models With Feature Summarization and Hybrid Retrieval-Augmented Generation: Multicenter Methodological Study Based on Radiology Report. J Med Internet Res. 2025 Jun 11;27:e72638. PMID: 40499132. doi: 10.2196/72638.

301. Miaojiao S, Xia L, Xian Tao Z, Zhi Liang H, Sheng C, Songsong W. Using a Large Language Model for Breast Imaging Reporting and Data System Classification and Malignancy Prediction to Enhance Breast Ultrasound Diagnosis: Retrospective Study. JMIR Med Inform. 2025 Jun 11;13:e70924. PMID: 40498674. doi: 10.2196/70924.

302. Sato M, Yasaka K, Abe S, Kurashima J, Asari Y, Kiryu S, et al. Efficacy of a large language model in classifying branch-duct intraductal papillary mucinous neoplasms. Abdominal radiology (New York). 2025 Jun 11. PMID: 40498341. doi: 10.1007/s00261-025-05062-z.

303. Attal K, Charalambous L, Di Gangi C, Rozell JC. Using Deep Learning With Few-Shot Learning to Improve Data Capture in Total Hip Arthroplasty Operative Notes. The Journal of arthroplasty. 2025 Jun 6. PMID: 40484056. doi: 10.1016/j.arth.2025.06.008.

304. Verkijk S, Vossen P. Creating, anonymizing and evaluating the first medical language model pre-trained on Dutch Electronic Health Records: MedRoBERTa.nl. Artif Intell Med. 2025 Sep;167:103148. PMID: 40472749. doi: 10.1016/j.artmed.2025.103148.

305. Alyanak B, Cakar I, Dede BT, Yildizgoren MT, Bagcier F. Artificial intelligence vs human expertise: A comparison of plantar fascia thickness measurements through MRI imaging. Int J Med Inform. 2025 Nov;203:105999. PMID: 40472563. doi: 10.1016/j.ijmedinf.2025.105999.

306. Martin-Noguerol T, Lopez-Ubeda P, Luna A, Gomez-Rio M, Gorriz JM. Role of Large Language Models for Suggesting Nerve Involvement in Upper Limbs MRI Reports with Muscle Denervation Signs. Clinical neuroradiology. 2025 Jun 5. PMID: 40471306. doi: 10.1007/s00062-025-01533-4.

307. Bertolli E, Micheletti SB, de Camargo VP, da Silva TV, Bacchi CE, Buzaid AC. Nomograms versus artificial intelligence platforms: which one can better predict sentinel node positivity in melanoma patients? Melanoma Res. 2025 Aug 1;35(4):227-31. PMID: 40465322. doi: 10.1097/CMR.0000000000001047.

308. Egaimi M, Corvo P, Al Houri H, Chang JM, Seo H, Almorraweh A, et al. Development and Technical Validation of an Integrated Risk Calculator for Acute Coronary Syndrome Using ChatGPT-Assisted Coding. Cureus. 2025 May;17(5):e83410. PMID: 40462782. doi: 10.7759/cureus.83410.

309. Chen YH, Ruan SJ, Chen PF. Predicting 30-Day Postoperative Mortality and American Society of Anesthesiologists Physical Status Using Retrieval-Augmented Large Language Models: Development and Validation Study. J Med Internet Res. 2025 Jun 3;27:e75052. PMID: 40460423. doi: 10.2196/75052.

310. Cheng Y, Malekar M, He Y, Bommareddy A, Magdamo C, Singh A, et al. High-Throughput Phenotyping of the Symptoms of Alzheimer Disease and Related Dementias Using Large Language Models: Cross-Sectional Study. Jmir ai. 2025 Jun 3;4:e66926. PMID: 40460418. doi: 10.2196/66926.

311. Yang X, Zhang Y, Jiang J, Chen Z, Bai R, Yuan Z, et al. Harnessing GPT-4 for automated error detection in pathology reports: Implications for oncology diagnostics. Digit Health. 2025 Jan-Dec;11:20552076251346703. PMID: 40453047. doi: 10.1177/20552076251346703.

312. Yang X, Xu J, Ji H, Li J, Yang B, Wang L. Early prediction of colorectal adenoma risk: leveraging large-language model for clinical electronic medical record data. Front Oncol. 2025;15:1508455. PMID: 40444092. doi: 10.3389/fonc.2025.1508455.

313. Wang K, Lin L, Zheng R, Nan S, Lu X, Duan H. Leveraging large language models for preoperative prevention of cardiopulmonary bypass-associated acute kidney injury. Ren Fail. 2025 Dec;47(1):2509786. PMID: 40442891. doi: 10.1080/0886022X.2025.2509786.

314. Rust P, Frings J, Meister S, Fehring L. Evaluation of a large language model to simplify discharge summaries and provide cardiological lifestyle recommendations. Commun Med (Lond). 2025 May 29;5(1):208. PMID: 40442348. doi: 10.1038/s43856-025-00927-2.

315. Chen R, Zhang S, Zheng Y, Yu Q, Wang C. Enhancing treatment decision-making for low back pain: a novel framework integrating large language models with retrieval-augmented generation technology. Front Med (Lausanne). 2025;12:1599241. PMID: 40438365. doi: 10.3389/fmed.2025.1599241.

316. Lu X, Gao X, Wang X, Gong Z, Cheng J, Hu W, et al. Comparison of medical history documentation efficiency and quality based on GPT-4o: a study on the comparison between residents and artificial intelligence. Front Med (Lausanne). 2025;12:1545730. PMID: 40438356. doi: 10.3389/fmed.2025.1545730.

317. Polis B, Zawadzka-Fabijan A, Fabijan R, Kosinska R, Nowoslawska E, Fabijan A. Comparative Evaluation of Large Language and Multimodal Models in Detecting Spinal Stabilization Systems on X-Ray Images. J Clin Med. 2025 May 8;14(10). PMID: 40429276. doi: 10.3390/jcm14103282.

318. Andras D, Ilies RA, Esanu V, Agoston S, Marginean Jumate TF, Dindelegan GC. Artificial Intelligence as a Potential Tool for Predicting Surgical Margin Status in Early Breast Cancer Using Mammographic Specimen Images. Diagnostics (Basel, Switzerland). 2025 May 17;15(10). PMID: 40428269. doi: 10.3390/diagnostics15101276.

319. Kenig N, Monton Echeverria J, Muntaner Vives A. Evaluating Surgical Results in Breast Cancer with Artificial Intelligence. Aesthetic Plast Surg. 2025 May 27. PMID: 40425883. doi: 10.1007/s00266-025-04915-8.

320. Hein D, Christie A, Holcomb M, Xie B, Jain AJ, Vento J, et al. Iterative refinement and goal articulation to optimize large language models for clinical information extraction. NPJ Digit Med. 2025 May 23;8(1):301. PMID: 40410408. doi: 10.1038/s41746-025-01686-z.

321. Campellone TR, Flom M, Montgomery RM, Bullard L, Pirner MC, Pavez A, et al. Safety and User Experience of a Generative Artificial Intelligence Digital Mental Health Intervention: Exploratory Randomized Controlled Trial. J Med Internet Res. 2025 May 23;27:e67365. PMID: 40408143. doi: 10.2196/67365.

322. Lho SK, Park SC, Lee H, Oh DY, Kim H, Jang S, et al. Large Language Models and Text Embeddings for Detecting Depression and Suicide in Patient Narratives. JAMA Netw Open. 2025 May 1;8(5):e2511922. PMID: 40408109. doi: 10.1001/jamanetworkopen.2025.11922.

323. Satheakeerthy S, Stretton B, Tsimiklis J, Booth AE, Howson S, Evans S, et al. Zero-shot large language model application for surgical site infection auditing. Infection, disease & health. 2025 May 21. PMID: 40404536. doi: 10.1016/j.idh.2025.05.001.

324. Goodman KE, Robinson ML, Shams SM, Beccar-Varela P, Fiawoo S, Kwon N, et al. Identification of Long-Term Care Facility Residence From Admission Notes Using Large Language Models. JAMA Netw Open. 2025 May 1;8(5):e2512032. PMID: 40402496. doi: 10.1001/jamanetworkopen.2025.12032.

325. Asar EM, Ipek I, Bi Lge K. Customized GPT-4V(ision) for radiographic diagnosis: can large language model detect supernumerary teeth? BMC oral health. 2025 May 21;25(1):756. PMID: 40399904. doi: 10.1186/s12903-025-06163-3.

326. Liu J, Gu J, Tong M, Yue Y, Qiu Y, Zeng L, et al. Evaluating the Agreement Between ChatGPT-4 and Validated Mental Health Scales in Older Adults: A Cross-Sectional Study. The American journal of geriatric psychiatry : official journal of the American Association for Geriatric Psychiatry. 2025 Oct;33(10):1049-61. PMID: 40393915. doi: 10.1016/j.jagp.2025.04.213.

327. Bayala YLT, Zabsonre/Tiendrebeogo WJS, Ouedraogo DD, Kabore F, Sougue C, Yameogo AR, et al. Performance of the Large Language Models in African rheumatology: a diagnostic test accuracy study of ChatGPT-4, Gemini, Copilot, and Claude artificial intelligence. BMC Rheumatol. 2025 May 16;9(1):54. PMID: 40380276. doi: 10.1186/s41927-025-00512-z.

328. Kanani MM, Monawer A, Brown L, King WE, 3rd, Miller ZD, Venugopal N, et al. High-Performance Prompting for LLM Extraction of Compression Fracture Findings from Radiology Reports. Journal of imaging informatics in medicine. 2025 May 16. PMID: 40379860. doi: 10.1007/s10278-025-01530-6.

329. Shashikumar SP, Mohammadi S, Krishnamoorthy R, Patel A, Wardi G, Ahn JC, et al. Development and prospective implementation of a large language model based system for early sepsis prediction. NPJ Digit Med. 2025 May 17;8(1):290. PMID: 40379845. doi: 10.1038/s41746-025-01689-w.

330. Fan H, Rossetti SC, Thate J, Mugoya R, Lai AM, Yen PY. Semi-automated pipeline to accelerate multi-site flowsheet alignment and concept mapping in electronic health records. Journal of the American Medical Informatics Association : JAMIA. 2025 Jul 1;32(7):1140-8. PMID: 40378254. doi: 10.1093/jamia/ocaf076.

331. Pham HQT, Vo TTL, Nguyen TT, Nguyen NTK, Nguyen PMT, Tran NH, et al. Validity of ChatGPT in Assisting Diagnosis of Periventricular-Intraventricular Hemorrhage via Cranial Ultrasound Imaging in Very Preterm Infants. Cureus. 2025 Apr;17(4):e82300. PMID: 40376373. doi: 10.7759/cureus.82300.

332. Wei S, Hu A, Liang Y, Yang J, Yu L, Li W, et al. Feasibility study of automatic radiotherapy treatment planning for cervical cancer using a large language model. Radiation oncology (London, England). 2025 May 15;20(1):77. PMID: 40375332. doi: 10.1186/s13014-025-02660-5.

333. Zhang J, Zhou J, Zhou L, Ba Z. Extracting Multifaceted Characteristics of Patients With Chronic Disease Comorbidity: Framework Development Using Large Language Models. JMIR Med Inform. 2025 May 15;13:e70096. PMID: 40373298. doi: 10.2196/70096.

334. Thakkar V, Silverman GM, Kc A, Ingraham NE, Jones EK, King S, et al. A comparative analysis of large language models versus traditional information extraction methods for real-world evidence of patient symptomatology in acute and post-acute sequelae of SARS-CoV-2. PLoS One. 2025;20(5):e0323535. PMID: 40373001. doi: 10.1371/journal.pone.0323535.

335. Xu J, Wang J, Li J, Zhu Z, Fu X, Cai W, et al. Predicting Immunotherapy Response in Unresectable Hepatocellular Carcinoma: A Comparative Study of Large Language Models and Human Experts. Journal of medical systems. 2025 May 15;49(1):64. PMID: 40372503. doi: 10.1007/s10916-025-02192-1.

336. Prontera PP, Prusciano FR, Lattarulo M, Tsaturyan A, Addabbo F, Sciorio C, et al. ChatGPT artificial intelligence in clinical data analysis: an example comparing standard vs fusion prostate biopsy outcomes after robotic-assisted radical prostatectomy (RaRP). Archivio italiano di urologia, andrologia : organo ufficiale [di] Societa italiana di ecografia urologica e nefrologica. 2025 Jun 30;97(2):13596. PMID: 40372170. doi: 10.4081/aiua.2025.13596.

337. Mykhalko Y, Dyditska S, Balatska L, Filak F, Rubtsova Y. AI-driven rehabilitation: evaluation of ChatGPT-4o for generating personalized physical rehabilitation plans in comorbid patients. Wiadomosci lekarskie (Warsaw, Poland : 1960). 2025;78(4):753-9. PMID: 40367458. doi: 10.36740/WLek/203850.

338. Xie Y, Hu Z, Tao H, Hu Y, Liang H, Lu X, et al. Large language models for efficient whole-organ MRI score-based reports and categorization in knee osteoarthritis. Insights Imaging. 2025 May 14;16(1):100. PMID: 40366500. doi: 10.1186/s13244-025-01976-w.

339. Vrdoljak J, Boban Z, Males I, Skrabic R, Kumric M, Ottosen A, et al. Evaluating large language and large reasoning models as decision support tools in emergency internal medicine. Comput Biol Med. 2025 Jun;192(Pt B):110351. PMID: 40359675. doi: 10.1016/j.compbiomed.2025.110351.

340. Pamuk E, Bilen YE, Kulekci C, Kuscu O. ChatGPT-4 vs. multi-disciplinary tumor board decisions for the therapeutic management of primary laryngeal cancer. Acta oto-laryngologica. 2025 Aug;145(8):714-9. PMID: 40358250. doi: 10.1080/00016489.2025.2502563.

341. Ganzinger M, Kunz N, Fuchs P, Lyu CK, Loos M, Dugas M, et al. Automated generation of discharge summaries: leveraging large language models with clinical data. Sci Rep. 2025 May 12;15(1):16466. PMID: 40355506. doi: 10.1038/s41598-025-01618-7.

342. Tian W, Huang X, Cheng T, He W, Fang J, Feng R, et al. A Medical Multimodal Large Language Model for Pediatric Pneumonia. IEEE J Biomed Health Inform. 2025 May 12;PP. PMID: 40354198. doi: 10.1109/JBHI.2025.3569361.

343. McCoy TH, Perlis RH. Reasoning language models for more transparent prediction of suicide risk. BMJ mental health. 2025 May 11;28(1). PMID: 40350181. doi: 10.1136/bmjment-2025-301654.

344. Najjar E, Abdelazim Hassan A, Muscogliati R, Salem KM, Quraishi NA. Human versus machine: deciding on high-stakes surgery in possible Cauda Equina syndrome. The spine journal : official journal of the North American Spine Society. 2025 May 8. PMID: 40348281. doi: 10.1016/j.spinee.2025.05.026.

345. Sheng L, Chen Y, Wei H, Che F, Wu Y, Qin Q, et al. Large Language Models for Diagnosing Focal Liver Lesions From CT/MRI Reports: A Comparative Study With Radiologists. Liver international : official journal of the International Association for the Study of the Liver. 2025 Jun;45(6):e70115. PMID: 40347005. doi: 10.1111/liv.70115.

346. Avidan Y, Naoum I, Khoury R, Zahra S, Dov NB, Schliamser JE, et al. Can ChatGPT accurately detect atrial fibrillation using smartwatch ECG? Heart & lung : the journal of critical care. 2025 Sep-Oct;73:90-4. PMID: 40345017. doi: 10.1016/j.hrtlng.2025.04.032.

347. Maia-Lima MP, Carvalho LIM, Araujo EGO, Martins HDD, Machado RA, Sobrinho LMF, et al. Performance of a virtual assistant based on ChatGPT-4 in the diagnosis of syndromes with orofacial manifestations. Oral surgery, oral medicine, oral pathology and oral radiology. 2025 Sep;140(3):322-9. PMID: 40340214. doi: 10.1016/j.oooo.2025.04.002.

348. Islam S. Evaluating the impact of AI-generated educational content on patient understanding and anxiety in endodontics and restorative dentistry: a comparative study. BMC oral health. 2025 May 7;25(1):689. PMID: 40335999. doi: 10.1186/s12903-025-06069-0.

349. Mahmoudi E, Vahdati S, Chao CJ, Khosravi B, Misra A, Lopez-Jimenez F, et al. A comparative analysis of privacy-preserving large language models for automated echocardiography report analysis. Journal of the American Medical Informatics Association : JAMIA. 2025 Jul 1;32(7):1120-9. PMID: 40334045. doi: 10.1093/jamia/ocaf056.

350. Croxford E, Gao Y, Pellegrino N, Wong K, Wills G, First E, et al. Development and validation of the provider documentation summarization quality instrument for large language models. Journal of the American Medical Informatics Association : JAMIA. 2025 Jun 1;32(6):1050-60. PMID: 40323321. doi: 10.1093/jamia/ocaf068.

351. Guan H, Novoa-Laurentiev J, Zhou L. CD-Tron: Leveraging large clinical language model for early detection of cognitive decline from electronic health records. J Biomed Inform. 2025 Jun;166:104830. PMID: 40320101. doi: 10.1016/j.jbi.2025.104830.

352. Yang X, Xiao Y, Liu D, Deng H, Huang J, Zhou Y, et al. Cross language transformation of free text into structured lobectomy surgical records from a multi center study. Sci Rep. 2025 May 2;15(1):15417. PMID: 40316625. doi: 10.1038/s41598-025-97500-7.

353. Vithanage D, Deng C, Wang L, Yin M, Alkhalaf M, Zhang Z, et al. Adapting Generative Large Language Models for Information Extraction from Unstructured Electronic Health Records in Residential Aged Care: A Comparative Analysis of Training Approaches. Journal of healthcare informatics research. 2025 Jun;9(2):191-219. PMID: 40309133. doi: 10.1007/s41666-025-00190-z.

354. De Pellegrin L, Weinzierl A, Kappos EA, Lindenblatt N, Zucal I, Harder Y. Evaluating artificial intelligence in decision-making for surgical treatment of benign breast conditions. Journal of plastic, reconstructive & aesthetic surgery : JPRAS. 2025 Jun;105:189-95. PMID: 40305890. doi: 10.1016/j.bjps.2025.03.057.

355. Yang TT, Zheng HX, Cao S, Jing ML, Hu J, Zuo Y, et al. Harnessing an Artificial Intelligence-Based Large Language Model With Personal Health Record Capability for Personalized Information Support in Postsurgery Myocardial Infarction: Descriptive Qualitative Study. J Med Internet Res. 2025 Apr 30;27:e68762. PMID: 40305084. doi: 10.2196/68762.

356. Panzeri D, Laohawetwanit T, Akpinar R, De Carlo C, Belsito V, Terracciano L, et al. Assessing the diagnostic accuracy of ChatGPT-4 in the histopathological evaluation of liver fibrosis in MASH. Hepatology communications. 2025 May 1;9(5). PMID: 40304570. doi: 10.1097/HC9.0000000000000695.

357. Bhadila GY, Alhomied M, Mahmoud A, Farsi NJ. Accuracy of Artificial Intelligence in Making Diagnoses and Treatment Decisions in Pediatric Dentistry. Pediatric dentistry. 2025 Mar 15;47(2):73-8. PMID: 40296263.

358. Knox S, Aghamoosa S, Heider PM, Cutty M, Wright A, Scherbakov D, et al. AI approaches for phenotyping Alzheimer's disease and related dementias using electronic health records. Alzheimer's & dementia (New York, N Y). 2025 Apr-Jun;11(2):e70089. PMID: 40291122. doi: 10.1002/trc2.70089.

359. Vahabi A, Dastan AE, Gunay H. The role of artificial intelligence in predicting injured structures based on clinical images of lacerations in the volar aspect of the hand and forearm. Journal of hand and microsurgery. 2025 Jul;17(4):100255. PMID: 40290855. doi: 10.1016/j.jham.2025.100255.

360. Berzolla E, Gosnell GG, Chen L, Vonck C, Alaia E, Meislin R. Artificial Intelligence Large Language Models Improve Patient Comprehension of Radiologist Magnetic Resonance Imaging Reports. Arthroscopy : the journal of arthroscopic & related surgery : official publication of the Arthroscopy Association of North America and the International Arthroscopy Association. 2025 Apr 25. PMID: 40288466. doi: 10.1016/j.arthro.2025.04.033.

361. Saban M, Alon Y, Luxenburg O, Singer C, Hierath M, Karoussou Schreiner A, et al. Comparison of CT referral justification using clinical decision support and large language models in a large European cohort. European radiology. 2025 Apr 27. PMID: 40287868. doi: 10.1007/s00330-025-11608-y.

362. Lorenzoni G, Garbin A, Brigiari G, Papappicco CAM, Manfrin V, Gregori D. Large Language Models in Action: Supporting Clinical Evaluation in an Infectious Disease Unit. Healthcare (Basel, Switzerland). 2025 Apr 11;13(8). PMID: 40281830. doi: 10.3390/healthcare13080879.

363. Pasli S, Yadigaroglu M, Kirimli EN, Beser MF, Unutmaz I, Ayhan AO, et al. ChatGPT-supported patient triage with voice commands in the emergency department: A prospective multicenter study. The American journal of emergency medicine. 2025 Aug;94:63-70. PMID: 40273640. doi: 10.1016/j.ajem.2025.04.040.

364. Temel MH, Erden Y, Bagcier F. Evaluating artificial intelligence performance in medical image analysis: Sensitivity, specificity, accuracy, and precision of ChatGPT-4o on Kellgren-Lawrence grading of knee X-ray radiographs. The Knee. 2025 Aug;55:79-84. PMID: 40273525. doi: 10.1016/j.knee.2025.04.008.

365. Kim J, Chen ML, Rezaei SJ, Ramirez-Posada M, Caswell-Jin JL, Kurian AW, et al. Patient-Centered Research Through Artificial Intelligence to Identify Priorities in Cancer Care. JAMA Oncol. 2025 Jun 1;11(6):630-5. PMID: 40272833. doi: 10.1001/jamaoncol.2025.0694.

366. Zou Y, Ye R, Gao Y, Zhou J, Li Y, Chen W, et al. Comparison of triage performance among DRP tool, ChatGPT, and outpatient rehabilitation doctors. Sci Rep. 2025 Apr 23;15(1):14084. PMID: 40269240. doi: 10.1038/s41598-025-99216-0.

367. Hartsock I, Araujo C, Folio L, Rasool G. Improving Radiology Report Conciseness and Structure via Local Large Language Models. Journal of imaging informatics in medicine. 2025 Apr 21. PMID: 40259201. doi: 10.1007/s10278-025-01510-w.

368. Sushil M, Kennedy VE, Mandair D, Miao BY, Zack T, Butte AJ. CORAL: Expert-Curated Oncology Reports to Advance Language Model Inference. Nejm ai. 2024 Apr;1(4). PMID: 40255242. doi: 10.1056/aidbp2300110.

369. Castelli M, Sousa M, Vojtech I, Single M, Amstutz D, Maradan-Gachet ME, et al. Detecting neuropsychiatric fluctuations in Parkinson's Disease using patients' own words: the potential of large language models. NPJ Parkinsons Dis. 2025 Apr 18;11(1):79. PMID: 40251156. doi: 10.1038/s41531-025-00939-8.

370. Han W, Wan C, Shan R, Xu X, Chen G, Zhou W, et al. Evaluation of error detection and treatment recommendations in nucleic acid test reports using ChatGPT models. Clin Chem Lab Med. 2025 Aug 26;63(9):1698-708. PMID: 40249886. doi: 10.1515/cclm-2025-0089.

371. Mondal A, Naskar A, Roy Choudhury B, Chakraborty S, Biswas T, Sinha S, et al. Evaluating the Performance and Safety of Large Language Models in Generating Type 2 Diabetes Mellitus Management Plans: A Comparative Study With Physicians Using Real Patient Records. Cureus. 2025 Mar;17(3):e80737. PMID: 40248538. doi: 10.7759/cureus.80737.

372. Yang X, Xiao Y, Liu D, Shi H, Deng H, Huang J, et al. Enhancing Physician-Patient Communication in Oncology Using GPT-4 Through Simplified Radiology Reports: Multicenter Quantitative Study. J Med Internet Res. 2025 Apr 17;27:e63786. PMID: 40245397. doi: 10.2196/63786.

373. Maniaci A, Hoch CC, Sogalow L, Schmidl B, Lechien JR. AI in clinical decision-making: ChatGPT-4 vs. Llama2 for otolaryngology cases. Eur Arch Otorhinolaryngol. 2025 Jun;282(6):3293-302. PMID: 40220179. doi: 10.1007/s00405-025-09371-3.

374. Alsumait A, Deshmukh S, Wang C, Leffler CT. Triage of Patient Messages Sent to the Eye Clinic via the Electronic Medical Record: A Comparative Study on AI and Human Triage Performance. J Clin Med. 2025 Mar 31;14(7). PMID: 40217845. doi: 10.3390/jcm14072395.

375. Akdogan O, Uyar GC, Yesilbas E, Baskurt K, Malkoc NA, Ozdemir N, et al. Effect of a ChatGPT-based digital counseling intervention on anxiety and depression in patients with cancer: A prospective, randomized trial. European journal of cancer (Oxford, England : 1990). 2025 May 15;221:115408. PMID: 40215593. doi: 10.1016/j.ejca.2025.115408.

376. Isch EL, Guler M, Galantini G, Ottaway J, Chan SHT, Daneshpooy S, et al. Bridging the Coding Gap: Assessing Large Language Models for Accurate Modifier Assignment in Craniofacial Operative Notes. The Journal of craniofacial surgery. 2025 Apr 11. PMID: 40214230. doi: 10.1097/SCS.0000000000011390.

377. Xue J, Wang Z, Chen N, Wu Y, Shen Z, Shao Y, et al. Evaluating multimodal ChatGPT for emergency decision-making of ocular trauma cases. Front Cell Dev Biol. 2025;13:1564054. PMID: 40213397. doi: 10.3389/fcell.2025.1564054.

378. Lilli L, Santoro M, Masiello V, Patarnello S, Tagliaferri L, Marazzi F, et al. MISTIC: a novel approach for metastasis classification in Italian electronic health records using transformers. BMC Med Inform Decis Mak. 2025 Apr 10;25(1):160. PMID: 40211241. doi: 10.1186/s12911-025-02994-w.

379. Salmanpour F, Akpinar M. Performance of Chat Generative Pretrained Transformer-4.0 in determining labiolingual localization of maxillary impacted canine and presence of resorption in incisors through panoramic radiographs: A retrospective study. American journal of orthodontics and dentofacial orthopedics : official publication of the American Association of Orthodontists, its constituent societies, and the American Board of Orthodontics. 2025 Aug;168(2):220-31. PMID: 40208160. doi: 10.1016/j.ajodo.2025.02.017.

380. Mahyoub M, Dougherty K, Shukla A. Extracting Pulmonary Embolism Diagnoses From Radiology Impressions Using GPT-4o: Large Language Model Evaluation Study. JMIR Med Inform. 2025 Apr 9;13:e67706. PMID: 40203306. doi: 10.2196/67706.

381. Yuan Y, Zhang G, Gu Y, Hao S, Huang C, Xie H, et al. Artificial intelligence-assisted machine learning models for predicting lung cancer survival. Asia-Pacific journal of oncology nursing. 2025 Dec;12:100680. PMID: 40201531. doi: 10.1016/j.apjon.2025.100680.

382. Pan J, Lee S, Cheligeer C, Martin EA, Riazi K, Quan H, et al. Integrating large language models with human expertise for disease detection in electronic health records. Comput Biol Med. 2025 Jun;191:110161. PMID: 40198990. doi: 10.1016/j.compbiomed.2025.110161.

383. Omar M, Soffer S, Agbareia R, Bragazzi NL, Apakama DU, Horowitz CR, et al. Sociodemographic biases in medical decision making by large language models. Nat Med. 2025 Jun;31(6):1873-81. PMID: 40195448. doi: 10.1038/s41591-025-03626-6.

384. Lee D, Vaid A, Menon KM, Freeman R, Matteson DS, Marin ML, et al. Using Large Language Models to Automate Data Extraction From Surgical Pathology Reports: Retrospective Cohort Study. JMIR formative research. 2025 Apr 7;9:e64544. PMID: 40194317. doi: 10.2196/64544.

385. McCoy TH, Castro VM, Perlis RH. Estimating depression severity in narrative clinical notes using large language models. Journal of affective disorders. 2025 Jul 15;381:270-4. PMID: 40187432. doi: 10.1016/j.jad.2025.04.014.

386. Palominos C, Kirdun M, Nikzad AH, Spilka MJ, Homan P, Sommer IE, et al. A single composite index of semantic behavior tracks symptoms of psychosis over time. Schizophr Res. 2025 May;279:116-27. PMID: 40187184. doi: 10.1016/j.schres.2025.03.038.

387. Wright DS, Socrates V, Huang T, Safranek CW, Sangal RB, Dilip M, et al. Automated computation of the HEART score with the GPT-4 large language model. The American journal of emergency medicine. 2025 Jul;93:120-5. PMID: 40184662. doi: 10.1016/j.ajem.2025.03.065.

388. Liu Y, Zhang X, Cao W, Cui W, Tan T, Peng Y, et al. Bootstrapping BI-RADS classification using large language models and transformers in breast magnetic resonance imaging reports. Visual computing for industry, biomedicine, and art. 2025 Apr 3;8(1):8. PMID: 40178668. doi: 10.1186/s42492-025-00189-8.

389. Wang Y, Zhu T, Zhou T, Wu B, Tan W, Ma K, et al. Hyper-DREAM, a Multimodal Digital Transformation Hypertension Management Platform Integrating Large Language Model and Digital Phenotyping: Multicenter Development and Initial Validation Study. Journal of medical systems. 2025 Apr 2;49(1):42. PMID: 40172683. doi: 10.1007/s10916-025-02176-1.

390. Zambrano Chaves JM, Huang SC, Xu Y, Xu H, Usuyama N, Zhang S, et al. A clinically accessible small multimodal radiology model and evaluation metric for chest X-ray findings. Nat Commun. 2025 Apr 1;16(1):3108. PMID: 40169573. doi: 10.1038/s41467-025-58344-x.

391. Akbasli IT, Birbilen AZ, Teksam O. Leveraging large language models to mimic domain expert labeling in unstructured text-based electronic healthcare records in non-english languages. BMC Med Inform Decis Mak. 2025 Mar 31;25(1):154. PMID: 40165165. doi: 10.1186/s12911-025-02871-6.

392. Grothey B, Odenkirchen J, Brkic A, Schomig-Markiefka B, Quaas A, Buttner R, et al. Comprehensive testing of large language models for extraction of structured data in pathology. Commun Med (Lond). 2025 Mar 31;5(1):96. PMID: 40164789. doi: 10.1038/s43856-025-00808-8.

393. Cork SC, Hopcroft K. Evaluating ChatGPT for converting clinic letters into patient-friendly language: a quantitative study. BJGP open. 2025 Jul 15. PMID: 40164490. doi: 10.3399/BJGPO.2024.0300.

394. West M, Cheng Y, He Y, Leng Y, Magdamo C, Hyman BT, et al. Unsupervised Deep Learning of Electronic Health Records to Characterize Heterogeneity Across Alzheimer Disease and Related Dementias: Cross-Sectional Study. JMIR aging. 2025 Mar 31;8:e65178. PMID: 40163031. doi: 10.2196/65178.

395. Durmazpinar PM, Ekmekci E. Comparing diagnostic skills in endodontic cases: dental students versus ChatGPT-4o. BMC oral health. 2025 Mar 29;25(1):457. PMID: 40158110. doi: 10.1186/s12903-025-05857-y.

396. Guo L, Zuo Y, Yisha Z, Liu J, Gu A, Yushan R, et al. Diagnostic performance of advanced large language models in cystoscopy: evidence from a retrospective study and clinical cases. BMC urology. 2025 Mar 29;25(1):64. PMID: 40158093. doi: 10.1186/s12894-025-01740-8.

397. Yilihamu EE, Zeng FS, Shang J, Yang JT, Zhong H, Feng SQ. GPT4LFS (generative pretrained transformer 4 omni for lumbar foramina stenosis): enhancing lumbar foraminal stenosis image classification through large multimodal models. The spine journal : official journal of the North American Spine Society. 2025 Sep;25(9):2071-80. PMID: 40157428. doi: 10.1016/j.spinee.2025.03.011.

398. Annen K, Andani S, Bosma G, Abbott D, Arinsburg S, Nguyen F, et al. O blood usage trends in the pediatric population 2015-2019: A multi-institutional analysis. Transfusion. 2025 Apr;65(4):676-83. PMID: 40151072. doi: 10.1111/trf.18225.

399. Chiesa-Estomba CM, Andueza-Guembe M, Maniaci A, Mayo-Yanez M, Betances-Reinoso F, Vaira LA, et al. Accuracy of ChatGPT-4o in Text and Video Analysis of Laryngeal Malignant and Premalignant Diseases. Journal of voice : official journal of the Voice Foundation. 2025 Mar 26. PMID: 40148204. doi: 10.1016/j.jvoice.2025.03.006.

400. Stanley J, Rabot E, Reddy S, Belilovsky E, Mottron L, Bzdok D. Large language models deconstruct the clinical intuition behind diagnosing autism. Cell. 2025 Apr 17;188(8):2235-48 e10. PMID: 40147442. doi: 10.1016/j.cell.2025.02.025.

401. Kreso A, Boban Z, Kabic S, Rada F, Batistic D, Barun I, et al. Using large language models as decision support tools in emergency ophthalmology. Int J Med Inform. 2025 Jul;199:105886. PMID: 40147415. doi: 10.1016/j.ijmedinf.2025.105886.

402. Roshani MA, Zhou X, Qiang Y, Suresh S, Hicks S, Sethuraman U, et al. Generative Large Language Model-Powered Conversational AI App for Personalized Risk Assessment: Case Study in COVID-19. Jmir ai. 2025 Mar 27;4:e67363. PMID: 40146990. doi: 10.2196/67363.

403. Adams MCB, Perkins ML, Hudson C, Madhira V, Akbilgic O, Ma D, et al. Breaking Digital Health Barriers Through a Large Language Model-Based Tool for Automated Observational Medical Outcomes Partnership Mapping: Development and Validation Study. J Med Internet Res. 2025 May 15;27:e69004. PMID: 40146872. doi: 10.2196/69004.

404. Somani S, Kim DD, Perez-Guerrero E, Ngo S, Seto T, Al-Kindi S, et al. Understanding Reasons for Oral Anticoagulation Nonprescription in Atrial Fibrillation Using Large Language Models. J Am Heart Assoc. 2025 Apr;14(7):e040419. PMID: 40145287. doi: 10.1161/JAHA.124.040419.

405. Marcaccini G, Seth I, Xie Y, Susini P, Pozzi M, Cuomo R, et al. Breaking Bones, Breaking Barriers: ChatGPT, DeepSeek, and Gemini in Hand Fracture Management. J Clin Med. 2025 Mar 14;14(6). PMID: 40142791. doi: 10.3390/jcm14061983.

406. Carla MM, Gambini G, Giannuzzi F, Boselli F, De Luca L, Rizzo S. Testing the Reliability of ChatGPT Assistance for Surgical Choices in Challenging Glaucoma Cases. Journal of personalized medicine. 2025 Feb 28;15(3). PMID: 40137413. doi: 10.3390/jpm15030097.

407. Erdat EC, Yalciner M, Oruncu MB, Urun Y, Senler FC. Assessing the accuracy of the GPT-4 model in multidisciplinary tumor board decision prediction. Clinical & translational oncology : official publication of the Federation of Spanish Oncology Societies and of the National Cancer Institute of Mexico. 2025 Mar 25. PMID: 40133589. doi: 10.1007/s12094-025-03905-1.

408. Hao Y, Holmes J, Hobson J, Bennett A, McKone EL, Ebner DK, et al. Retrospective Comparative Analysis of Prostate Cancer In-Basket Messages: Responses From Closed-Domain Large Language Models Versus Clinical Teams. Mayo Clinic proceedings Digital health. 2025 Mar;3(1). PMID: 40130001. doi: 10.1016/j.mcpdig.2025.100198.

409. Zhou J, Li X, Xia Q, Yu L. Innovations in otolaryngology using LLM for early detection of sleep-disordered breathing. SLAS Technol. 2025 Jun;32:100278. PMID: 40122382. doi: 10.1016/j.slast.2025.100278.

410. Zaboli A, Brigo F, Brigiari G, Massar M, Parodi M, Pfeifer N, et al. Chat-GPT in triage: Still far from surpassing human expertise - An observational study. The American journal of emergency medicine. 2025 Jun;92:165-71. PMID: 40120387. doi: 10.1016/j.ajem.2025.03.028.

411. Yuan K, Yoon CH, Gu Q, Munby H, Walker AS, Zhu T, et al. Transformers and large language models are efficient feature extractors for electronic health record studies. Commun Med (Lond). 2025 Mar 21;5(1):83. PMID: 40119150. doi: 10.1038/s43856-025-00790-1.

412. Schaye V, DiTullio D, Guzman BV, Vennemeyer S, Shih H, Reinstein I, et al. Large Language Model-Based Assessment of Clinical Reasoning Documentation in the Electronic Health Record Across Two Institutions: Development and Validation Study. J Med Internet Res. 2025 Mar 21;27:e67967. PMID: 40117575. doi: 10.2196/67967.

413. Mansoor M, Ibrahim AF, Grindem D, Baig A. Large Language Models for Pediatric Differential Diagnoses in Rural Health Care: Multicenter Retrospective Cohort Study Comparing GPT-3 With Pediatrician Performance. JMIRx med. 2025 Mar 19;6:e65263. PMID: 40106452. doi: 10.2196/65263.

414. Agarwal S, Wood D, Murray BAK, Wei Y, Busaidi AA, Kafiabadi S, et al. Impact of hospital-specific domain adaptation on BERT-based models to classify neuroradiology reports. European radiology. 2025 Sep;35(9):5299-313. PMID: 40097844. doi: 10.1007/s00330-025-11500-9.

415. Grinberg N, Whitefield S, Kleinman S, Ianculovici C, Wasserman G, Peleg O. Assessing the performance of an artificial intelligence based chatbot in the differential diagnosis of oral mucosal lesions: clinical validation study. Clinical oral investigations. 2025 Mar 18;29(4):188. PMID: 40097790. doi: 10.1007/s00784-025-06268-7.

416. Alam SF, Thongprayoon C, Miao J, Pham JH, Sheikh MS, Garcia Valencia OA, et al. Advancing personalized medicine in digital health: The role of artificial intelligence in enhancing clinical interpretation of 24-h ambulatory blood pressure monitoring. Digit Health. 2025 Jan-Dec;11:20552076251326014. PMID: 40093710. doi: 10.1177/20552076251326014.

417. Singh R, Hamouda M, Chamberlin JH, Toth A, Munford J, Silbergleit M, et al. ChatGPT vs. Gemini: Comparative accuracy and efficiency in Lung-RADS score assignment from radiology reports. Clin Imaging. 2025 May;121:110455. PMID: 40090067. doi: 10.1016/j.clinimag.2025.110455.

418. Karacay P, Goktas P, Yasar O, Uyanik B, Uzlu S, Coskun K, et al. Investigation of Pressure Injuries With Visual ChatGPT Integration: A Descriptive Cross-Sectional Study. Journal of advanced nursing. 2025 Mar 14. PMID: 40084802. doi: 10.1111/jan.16905.

419. Hanna JJ, Wakene AD, Johnson AO, Lehmann CU, Medford RJ. Assessing Racial and Ethnic Bias in Text Generation by Large Language Models for Health Care-Related Tasks: Cross-Sectional Study. J Med Internet Res. 2025 Mar 13;27:e57257. PMID: 40080818. doi: 10.2196/57257.

420. Shahid F, Hsu MH, Chang YC, Jian WS. Using Generative AI to Extract Structured Information from Free Text Pathology Reports. Journal of medical systems. 2025 Mar 13;49(1):36. PMID: 40080229. doi: 10.1007/s10916-025-02167-2.

421. Ito R, Kato K, Nanataki K, Abe Y, Ogawa H, Minamimoto R, et al. Assessing large language models for Lugano classification of malignant lymphoma in Japanese FDG-PET reports. EJNMMI reports. 2025 Mar 10;9(1):8. PMID: 40059276. doi: 10.1186/s41824-025-00246-8.

422. Bartley M, Huemann Z, Hu J, Tie X, Ross AB, Kennedy T, et al. Artificial Intelligence for Teaching Case Curation: Evaluating Model Performance on Imaging Report Discrepancies. Acad Radiol. 2025 Jun;32(6):3139-46. PMID: 40058990. doi: 10.1016/j.acra.2025.02.011.

423. Atsukawa N, Tatekawa H, Oura T, Matsushita S, Horiuchi D, Takita H, et al. Evaluation of radiology residents' reporting skills using large language models: an observational study. Japanese journal of radiology. 2025 Jul;43(7):1204-12. PMID: 40056344. doi: 10.1007/s11604-025-01764-y.

424. Kim SH, Wihl J, Schramm S, Berberich C, Rosenkranz E, Schmitzer L, et al. Human-AI collaboration in large language model-assisted brain MRI differential diagnosis: a usability study. European radiology. 2025 Sep;35(9):5252-63. PMID: 40055233. doi: 10.1007/s00330-025-11484-6.

425. Zhu J, Jiang Y, Chen D, Lu Y, Huang Y, Lin Y, et al. High identification and positive-negative discrimination but limited detailed grading accuracy of ChatGPT-4o in knee osteoarthritis radiographs. Knee surgery, sports traumatology, arthroscopy : official journal of the ESSKA. 2025 May;33(5):1911-9. PMID: 40053915. doi: 10.1002/ksa.12639.

426. Shmilovitch AH, Katson M, Cohen-Shelly M, Peretz S, Aran D, Shelly S. GPT-4 as a Clinical Decision Support Tool in Ischemic Stroke Management: Evaluation Study. Jmir ai. 2025 Mar 7;4:e60391. PMID: 40053715. doi: 10.2196/60391.

427. Encalada S, Gupta S, Hunt C, Eldrige J, Evans J, 2nd, Mosquera-Moscoso J, et al. Optimizing patient understanding of spine MRI reports using AI: A prospective single center study. Interventional pain medicine. 2025 Mar;4(1):100550. PMID: 40051774. doi: 10.1016/j.inpm.2025.100550.

428. Munzir SI, Hier DB, Carrithers MD. High Throughput Phenotyping of Physician Notes with Large Language and Hybrid NLP Models. Annual International Conference of the IEEE Engineering in Medicine and Biology Society IEEE Engineering in Medicine and Biology Society Annual International Conference. 2024 Jul;2024:1-5. PMID: 40039752. doi: 10.1109/EMBC53108.2024.10782119.

429. MacKay EJ, Goldfinger S, Chan TJ, Grasfield RH, Eswar VJ, Li K, et al. Automated structured data extraction from intraoperative echocardiography reports using large language models. British journal of anaesthesia. 2025 May;134(5):1308-17. PMID: 40037947. doi: 10.1016/j.bja.2025.01.028.

430. Yazla M, Sarcan E. Compliance Evaluation with ChatGPT for Diagnosis and Treatment in Patients Brought to the ED with a Preliminary Diagnosis of Stroke. Prehospital emergency care. 2025;29(3):243-51. PMID: 40036089. doi: 10.1080/10903127.2025.2475513.

431. Farrow L, Anderson L, Zhong M. Managing class imbalance in the training of a large language model to predict patient selection for total knee arthroplasty: Results from the Artificial intelligence to Revolutionise the patient Care pathway in Hip and knEe aRthroplastY (ARCHERY) project. The Knee. 2025 Jun;54:1-8. PMID: 40020253. doi: 10.1016/j.knee.2025.02.007.

432. Noda M, Takahara S, Hayashi S, Inui A, Oe K, Matsushita T. Evaluating ChatGPT's Performance in Classifying Pertrochanteric Fractures Based on Arbeitsgemeinschaft fur Osteosynthesefragen/Orthopedic Trauma Association (AO/OTA) Standards. Cureus. 2025 Jan;17(1):e78068. PMID: 40018458. doi: 10.7759/cureus.78068.

433. Maghsoudi A, Sharafkhaneh A, Azarian M, Ramezani A, Hirshkowitz M, Razjouyan J. A case study on generative artificial intelligence to extract the fundamental sleep parameters from polysomnography notes. Journal of clinical sleep medicine : JCSM : official publication of the American Academy of Sleep Medicine. 2025 Jun 1;21(6):1123-7. PMID: 40012317. doi: 10.5664/jcsm.11594.

434. Kang D, Wu H, Yuan L, Shen W, Feng J, Zhan J, et al. Evaluating the Efficacy of Large Language Models in Guiding Treatment Decisions for Pediatric Refractive Error. Ophthalmology and therapy. 2025 Apr;14(4):705-16. PMID: 39985747. doi: 10.1007/s40123-025-01105-2.

435. Salam B, Stuwe C, Nowak S, Sprinkart AM, Theis M, Kravchenko D, et al. Large language models for error detection in radiology reports: a comparative analysis between closed-source and privacy-compliant open-source models. European radiology. 2025 Aug;35(8):4549-57. PMID: 39979623. doi: 10.1007/s00330-025-11438-y.

436. Koegel LK, Ponder E, Bruzzese T, Wang M, Semnani SJ, Chi N, et al. Using Artificial Intelligence to Improve Empathetic Statements in Autistic Adolescents and Adults: A Randomized Clinical Trial. J Autism Dev Disord. 2025 Feb 15. PMID: 39954218. doi: 10.1007/s10803-025-06734-x.

437. Wu C, Liu W, Mei P, Liu Y, Cai J, Liu L, et al. The large language model diagnoses tuberculous pleural effusion in pleural effusion patients through clinical feature landscapes. Respiratory research. 2025 Feb 12;26(1):52. PMID: 39939874. doi: 10.1186/s12931-025-03130-y.

438. Sorin V, Kapelushnik N, Hecht I, Zloto O, Glicksberg BS, Bufman H, et al. Integrated visual and text-based analysis of ophthalmology clinical cases using a large language model. Sci Rep. 2025 Feb 10;15(1):4999. PMID: 39930078. doi: 10.1038/s41598-025-88948-8.

439. Mahbub M, Dams GM, Srinivasan S, Rizy C, Danciu I, Trafton J, et al. Decoding substance use disorder severity from clinical notes using a large language model. Npj mental health research. 2025 Feb 7;4(1):5. PMID: 39915681. doi: 10.1038/s44184-024-00114-6.

440. Xu X, Jiang R, Zheng S, Wang M, Ju Y, Li J. Classification of Chronic Dizziness Using Large Language Models. Journal of healthcare informatics research. 2025 Mar;9(1):88-102. PMID: 39897102. doi: 10.1007/s41666-024-00178-1.

441. Altalla B, Abdalla S, Altamimi A, Bitar L, Al Omari A, Kardan R, et al. Evaluating GPT models for clinical note de-identification. Sci Rep. 2025 Jan 31;15(1):3852. PMID: 39890969. doi: 10.1038/s41598-025-86890-3.

442. Schmidl B, Hutten T, Pigorsch S, Stogbauer F, Hoch CC, Hussain T, et al. Artificial intelligence for image recognition in diagnosing oral and oropharyngeal cancer and leukoplakia. Sci Rep. 2025 Jan 29;15(1):3625. PMID: 39880876. doi: 10.1038/s41598-025-85920-4.

443. Li KW, Lacson R, Guenette JP, DiPiro PJ, Burk KS, Kapoor N, et al. Use of ChatGPT Large Language Models to Extract Details of Recommendations for Additional Imaging From Free-Text Impressions of Radiology Reports. AJR American journal of roentgenology. 2025 Apr;224(4):e2432341. PMID: 39878409. doi: 10.2214/AJR.24.32341.

444. Mao X, Huang Y, Jin Y, Wang L, Chen X, Liu H, et al. A phenotype-based AI pipeline outperforms human experts in differentially diagnosing rare diseases using EHRs. NPJ Digit Med. 2025 Jan 28;8(1):68. PMID: 39875532. doi: 10.1038/s41746-025-01452-1.

445. Ma R, Cheng Q, Yao J, Peng Z, Yan M, Lu J, et al. Multimodal machine learning enables AI chatbot to diagnose ophthalmic diseases and provide high-quality medical responses. NPJ Digit Med. 2025 Jan 27;8(1):64. PMID: 39870855. doi: 10.1038/s41746-025-01461-0.

446. Yang X, Xiao Y, Liu D, Zhang Y, Deng H, Huang J, et al. Enhancing doctor-patient communication using large language models for pathology report interpretation. BMC Med Inform Decis Mak. 2025 Jan 23;25(1):36. PMID: 39849504. doi: 10.1186/s12911-024-02838-z.

447. Lopez I, Swaminathan A, Vedula K, Narayanan S, Nateghi Haredasht F, Ma SP, et al. Clinical entity augmented retrieval for clinical information extraction. NPJ Digit Med. 2025 Jan 19;8(1):45. PMID: 39828800. doi: 10.1038/s41746-024-01377-1.

448. Abbas S, Iftikhar M, Shah MM, Khan SJ. ChatGPT-Assisted Machine Learning for Chronic Disease Classification and Prediction: A Developmental and Validation Study. Cureus. 2024 Dec;16(12):e75851. PMID: 39822450. doi: 10.7759/cureus.75851.

449. Kelly BS, Duignan S, Mathur P, Dillon H, Lee EH, Yeom KW, et al. Can ChatGPT4-vision identify radiologic progression of multiple sclerosis on brain MRI? European radiology experimental. 2025 Jan 15;9(1):9. PMID: 39812885. doi: 10.1186/s41747-024-00547-w.

450. Basaran AE, Guresir A, Knoch H, Vychopen M, Guresir E, Wach J. Beyond traditional prognostics: integrating RAG-enhanced AtlasGPT and ChatGPT 4.0 into aneurysmal subarachnoid hemorrhage outcome prediction. Neurosurgical review. 2025 Jan 11;48(1):40. PMID: 39794551. doi: 10.1007/s10143-025-03194-w.

451. Sherif IA, Nser SY, Bobo A, Afridi A, Hamed A, Dunbar M, et al. Can Ordinary AI-Powered Tools Replace a Clinician-Led Fracture Clinic Appointment? Cureus. 2024 Dec;16(12):e75440. PMID: 39791069. doi: 10.7759/cureus.75440.

452. Celik E, Turgut MA, Aydogan M, Kilinc M, Toktas I, Akelma H. Comparison of AI applications and anesthesiologist's anesthesia method choices. BMC Anesthesiol. 2025 Jan 3;25(1):2. PMID: 39754097. doi: 10.1186/s12871-024-02882-2.

453. Scherbakov D, Heider PM, Wehbe R, Alekseyenko AV, Lenert LA, Obeid JS. Using large language models for extracting stressful life events to assess their impact on preventive colon cancer screening adherence. BMC Public Health. 2025 Jan 2;25(1):12. PMID: 39748338. doi: 10.1186/s12889-024-21123-2.

454. Shashikumar SP, Nemati S. A Prospective Comparison of Large Language Models for Early Prediction of Sepsis. Pacific Symposium on Biocomputing Pacific Symposium on Biocomputing. 2025;30:109-20. PMID: 39670365. doi: 10.1142/9789819807024_0009.

455. Ebner F, Hartkopf A, Veselinovic K, Schochter F, Janni W, Lukac S, et al. A Comparison of ChatGPT and Multidisciplinary Team Meeting Treatment Recommendations in 10 Consecutive Cervical Cancer Patients. Cureus. 2024 Aug;16(8):e67458. PMID: 39310414. doi: 10.7759/cureus.67458.

456. Sun P, Qian L, Wang Z. Preliminary experiments on interpretable ChatGPT-assisted diagnosis for breast ultrasound radiologists. Quantitative imaging in medicine and surgery. 2024 Sep 1;14(9):6601-12. PMID: 39281130. doi: 10.21037/qims-24-141.

457. Kuzan BN, Mese I, Yasar S, Kuzan TY. A retrospective evaluation of the potential of ChatGPT in the accurate diagnosis of acute stroke. Diagnostic and interventional radiology (Ankara, Turkey). 2025 Apr 28;31(3):187-95. PMID: 39221691. doi: 10.4274/dir.2024.242892.

458. Brin D, Sorin V, Barash Y, Konen E, Glicksberg BS, Nadkarni GN, et al. Assessing GPT-4 multimodal performance in radiological image analysis. European radiology. 2025 Apr;35(4):1959-65. PMID: 39214893. doi: 10.1007/s00330-024-11035-5.

459. Kruckel A, Bruckner L, Psilopatis I, Fasching PA, Beckmann MW, Emons J. Evaluation of ChatGPT's Potential in Tailoring Gynecological Cancer Therapies. In vivo (Athens, Greece). 2024 Jul-Aug;38(4):1649-59. PMID: 38936938. doi: 10.21873/invivo.13614.

460. Hu Y, Hu Z, Liu W, Gao A, Wen S, Liu S, et al. Exploring the potential of ChatGPT as an adjunct for generating diagnosis based on chief complaint and cone beam CT radiologic findings. BMC Med Inform Decis Mak. 2024 Feb 19;24(1):55. PMID: 38374067. doi: 10.1186/s12911-024-02445-y.

461. Dubinski D, Won SY, Trnovec S, Behmanesh B, Baumgarten P, Dinc N, et al. Leveraging artificial intelligence in neurosurgery-unveiling ChatGPT for neurosurgical discharge summaries and operative reports. Acta neurochirurgica. 2024 Jan 26;166(1):38. PMID: 38277081. doi: 10.1007/s00701-024-05908-3.

462. Raghu K, S T, C SD, M S, Rajalakshmi R, Raman R. The Utility of ChatGPT in Diabetic Retinopathy Risk Assessment: A Comparative Study with Clinical Diagnosis. Clinical ophthalmology (Auckland, NZ). 2023;17:4021-31. PMID: 38164506. doi: 10.2147/OPTH.S435052.

463. P PJ, Prasad SS, Manohar N. Genital and Extragenital Lichen Sclerosus et Atrophicus: A Case Series Written Using ChatGPT. Cureus. 2023 May;15(5):e38987. PMID: 37323348. doi: 10.7759/cureus.38987.

464. Ding H, Xia W, Zhou Y, Wei L, Feng Y, Wang Z, et al. Evaluation and practical application of prompt-driven ChatGPTs for EMR generation. NPJ Digit Med. 2025 Feb 2;8(1):77. PMID: 39894840. doi: 10.1038/s41746-025-01472-x.
